# Engineering a robust and autonomous biological funneling for efficient valorization of lignin-related aromatics

**DOI:** 10.1038/s41467-026-74424-y

**Published:** 2026-06-17

**Authors:** Jianping Xu, Jian Zhang, Xiaotong Wang, Tengfei Zhao, Qingsheng Qi, Qian Wang

**Affiliations:** 1 National Glycoengineering Research Center, Shandong University, Qingdao, P. R. China; 2 State Key Laboratory of Microbial Technology, Shandong University, Qingdao, P. R. China

**Keywords:** Metabolic engineering, Metabolic engineering, Synthetic biology, Applied microbiology

## Abstract

Lignin-related aromatics are promising renewable feedstocks, but their microbial conversion into useful compounds is fundamentally constrained by substrate toxicity and metabolic flux imbalance. Here, we report an autonomous microbial system in *Corynebacterium glutamicum*, which integrates evolutionary engineering with multiplexed dynamic control to overcome these constraints. This system features a robust, evolved chassis with reinforced cellular defenses and upgraded aromatic efflux machinery. We engineer a layered, multi-input regulatory network that enables real-time, substrate-responsive and precise control of metabolic pathways. This system achieves 99.89% conversion of diverse lignin-related aromatics to protocatechuic acid and subsequently achieves a titer of 53.40 g L⁻¹. This further supports high-titer production of value-added downstream chemicals, including *cis,cis*-muconic acid and β-ketoadipic acid with titers of 51.90 g L⁻¹ and 38.33 g L⁻¹, respectively. This work establishes a versatile and scalable paradigm for the autonomous bioprocessing of lignin feedstocks into sustainable value-added products.

## Abstract (web summary)

Biological valorization of lignin is hindered by substrate toxicity and metabolism inefficiency. Here, the authors established a platform for lignin-derived aromatics tolerance engineering and multi-node dynamic pathway control in *Corynebacterium glutamicum*.

## Introduction

Lignin, an abundant aromatic polymer that constitutes 15–40% of plant cell walls, represents the largest renewable aromatic source on Earth^1–3^. Although approximately 300 million tons of lignin are generated annually from agricultural residues (e.g., corn stover, rice straw, and sugarcane)^4^, pulp and paper industries, and biorefineries^5^, most of this material is currently burned or discarded, resulting in carbon emissions and leaving it among the least utilized renewable carbon resources^6^. Efficient valorization of lignin is therefore essential for sustainable biorefining^2,7–9^, yet: its structural heterogeneity complicates separation and purification^10,11^, while conventional chemical and enzymatic conversion methods suffer from high costs and operational complexity^12,13^.

In nature, lignin degradation relies on the synergistic actions of wood-decaying fungi^14–16^, which drive extracellular depolymerization, and bacteria, which have evolved efficient metabolic routes for monomer conversion^17,18^. A key feature of bacterial metabolism is the presence of biological funneling pathways, which converge structurally diverse aromatic monomers into a limited set of central intermediates^12,19^, thereby overcoming the inherent chemical complexity of lignin. Phenylpropanoids are ester pendent-linked to lignin and hemicellulose rather than core-integrated to lignin. Often counted as part of the total lignin in corn stover^20^, their valorization is crucial for lignin utilization^21^ because they are efficiently monomerized via alkaline depolymerization^22^. In terms of biological funneling pathways, phenylpropanoids, such as G-type ferulic acid (FA), H-type *p*-coumaric acid (*p*-CA), S-type sinapic acid (SA), and C-type caffeic acid (CaA)—are metabolized via β-oxidation^23^, nonoxidative decarboxylation^24^, or retro-aldol mechanisms^25^, leading to the conversion of these compounds toward central intermediates such as protocatechuic acid (PCA)^4,26,27^. This funneling strategy provides an inherent solution to lignin heterogeneity by enabling the convergence of multiple substrates into unified metabolic routes. However, the efficient operation of such pathways requires precise coordination of metabolic flux across multiple enzymatic steps, particularly when complex mixtures of phenylpropanoids derived from lignocellulose biomass are processed.

A variety of microbial hosts, including *Pseudomonas putida*^21,28–30^*, Rhodococcus jostii*^31–35^, *Escherichia coli*^36^, *Comamonas testosterone*^37^, *Sphingomonas paucimobilis SYK-6*^38,39^, and *Cupriavidus necator*^40^, have been used as platforms for lignin-related aromatic conversion, capitalizing on their species-specific metabolic features. In addition, research on the use of *Corynebacterium glutamicum* for lignin valorization has attracted increasing attention^22,41^. These efforts have expanded the spectrum of value-added products, ranging from pharmaceuticals and food additives^42–45^ to bioplastic precursors such as *cis,cis-muconic acid* (CCM) and β-ketoadipic acid (β-KA)^46–50^. Given their substantial global market value, the production of these chemicals from lignin-derived streams represents a more economical and sustainable alternative. However, most current strategies rely on static pathway engineering and lack the ability to dynamically coordinate flux across heterogeneous substrates. As a result, substrate toxicity, metabolic imbalance, and intermediate accumulation remain major bottlenecks, particularly under industrially relevant conditions.

*C. glutamicum* has emerged as a promising chassis for lignin valorization because of its intrinsic aromatic catabolic capacity, robust metabolism, and industrial applicability. It possesses inherent biological funneling pathways and has been engineered to produce value-added chemicals from lignin-related substrates^6,22,41^, offering several advantages including efficient aromatic ring-cleavage^51,52^, robust redox regeneration, and high biomass accumulation^53–55^. Nevertheless, its potential for the coordinated conversion of diverse lignin-related aromatics remains underexploited. Engineered strains still exhibit limited tolerance to aromatic compounds and insufficient control over metabolic flux distribution^41,56^. Moreover, although several lignin-related aromatic-responsive allosteric transcription factors (aTFs) have been identified^51^, their regulatory mechanisms remain poorly understood. Consequently, there is a lack of integrated, layered, and multi-input biological funneling systems capable of coordinating flux across diverse aromatic substrates, particularly under industrially relevant conditions.

**Fig. 1:**
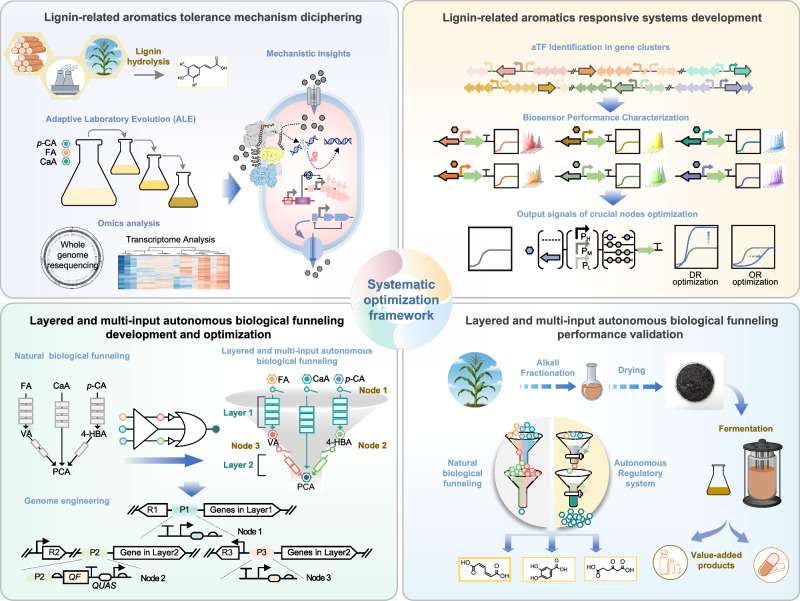
Overview of the pipelined systematic optimization framework based on layered and multi-input autonomous dynamic control system in *C. glutamicum.* This framework encompasses the following components: (1) acquisition of a robust chassis cell via Adaptive Laboratory Evolution (ALE) coupled with elucidation and validation of its tolerance mechanisms; (2) screening, identification, and systematic development of biosensors’ elements for lignin-related aromatic compounds; and (3) assembly of the layered and multi-input autonomous dynamic control system with adjustment, adaptation, and performance validation of three key nodes. The resultant robust *C. glutamicum* platform based on the layered and multi-input autonomous dynamic control system achieved temporal and quantitative control of metabolic pathway genes for lignin monomers, thereby enabling efficient conversion of lignin monomers into protocatechuic acid, *cis,cis*-muconic acid, and β-ketoadipic acid.

In this work, we develop an integrated engineering framework that combines adaptive laboratory evolution, biosensor engineering, and multiplexed dynamic control to elucidate the full potential of *C. glutamicum* for the conversion of lignin-related aromatic compounds (Fig. 1). We enhance the tolerance of *C. glutamicum* to lignin-related aromatics through adaptive laboratory evolution (ALE), identify and validate key genetic targets, and elucidate the underlying mechanisms. We also systematically characterize multiple native aromatic-responsive aTFs and engineer them into biosensors with a high dynamic range and broad operational range. Furthermore, we develop a layered, multi-input autonomous dynamic control system that enables real-time control of biological funneling pathways in response to fluctuating substrate and intermediate concentrations. This autonomous system supports efficient and coordinated conversion of lignin-related phenylpropanoids into central intermediates and downstream value-added products, including PCA, CCM, and β-KA, with a maximum conversion efficiency of 99.98%. The PCA- and CCM-producing strains achieve competitive titers of 53.40 and 51.90 g L^−^¹, respectively, under industrially relevant bioprocessing conditions. These results underscore the potential of our systematic optimization framework based on a layered and multi-input autonomous dynamic control system for lignin-related aromatic valorization in *C. glutamicum*.

## Results

### Metabolic limitations of native biological funneling in *C. glutamicum*

**Fig. 2:**
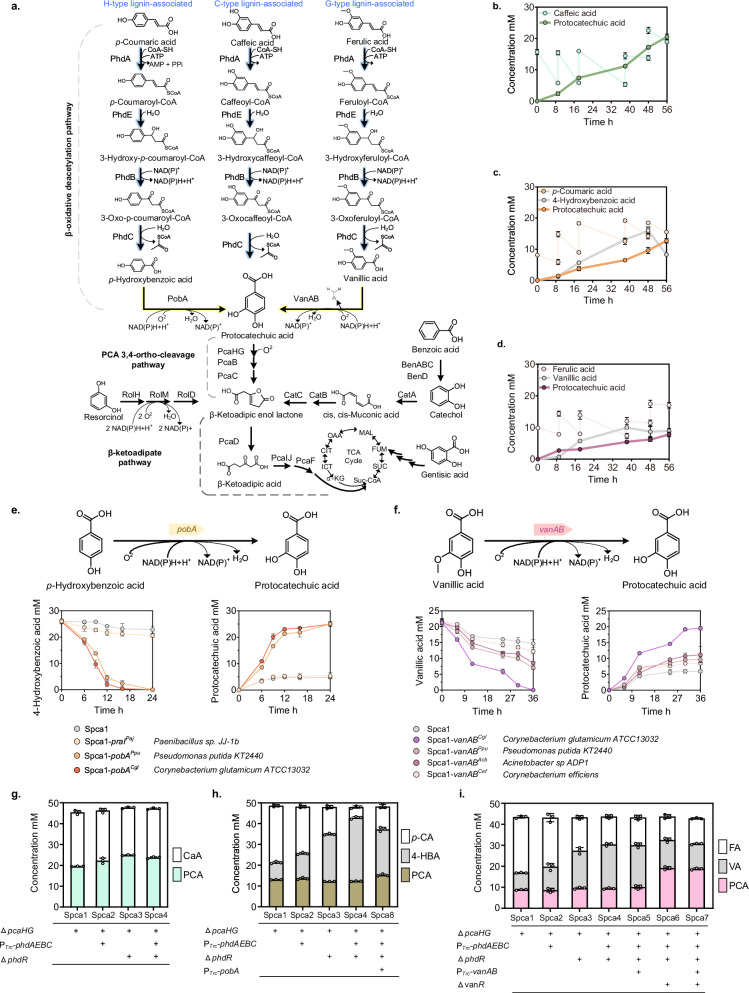
Metabolic engineering of the biological funneling pathway in *C. glutamicum.* **a** Biological funneling pathway in *C. glutamicum*. Blue arrows indicate Layer 1 enzymes: PhdA, acyl-CoA ligase; PhdE, enoyl-CoA hydratase; PhdB, 3-hydroxyacyl-CoA dehydrogenase; PhdC, 3-oxoacyl-CoA ketohydrolase. Yellow arrows indicate Layer 2 enzymes: PobA, 4-hydroxybenzoate-3-hydroxylase; VanAB, vanillate O-demethylase. Black arrows indicate PCA ring-cleavage and other lignin-related aromatics degradation pathways: PcaHG, protocatechuate-3,4-dioxygenase; PcaB, β-carboxy-*cis,cis*-muconate lactonizing enzyme; PcaC, γ-carboxymuconolactone decarboxylase; PcaD, β-ketoadipate enol-lactone hydrolase; PcaIJ, β-ketoadipate succinyl-CoA transferase; PcaF, β-ketoadipyl-CoA thiolase; RolH, resorcinol hydroxylase; RolM, maleylacetate reductase; RolD, hydroxyquinol 1,2-dioxygenase; BenABC, benzoate dioxygenase; BenD, benzoate diol dehydrogenase; CatA, catechol-1,2-dioxygenase; CatB, *cis,cis*-muconate cycloisomerase; CatC, muconolactone isomerase. **b**–**d** CaA, *p*-CA, and FA conversion by Spca1 under five-pulse feeding. **b** CaA consumption (light mint green) and PCA production (dark green) by Spca1 fed with 53.34 mM CaA. **c** *p*-CA consumption (light orange), 4-HBA (grey), and PCA production (dark orange) by Spca1 fed with 41.90 mM *p*-CA. **d** FA consumption (light pink), VA production (grey), and PCA production (dark pink) by Spca1 fed with 40.62 mM FA. **e**, **f** Screening of heterologous 4-hydroxybenzoate-3-hydroxylases (**e**) and vanillate O-demethylases (**f**) from different sources by monitoring 4-HBA or VA consumption and PCA production. Candidate enzymes were plasmid-expressed. **g**–**i** Product distribution of engineered strains under stepwise feeding (56 h, five pulses as in **b**–**d**). Total bar heights represent the sum of products and residual substrates, plus signs indicate engineering strategies. **g** PCA production from CaA by Spca1–Spca4 fed 45.45, 46.35, 47.68, and 47.22 mM CaA. Bar: PCA, mint green; CaA, unfilled. **h** PCA and 4-HBA production from *p*-CA by Spca1–Spca4 and Spca8 fed with 48.65, 48.22, 48.00, 47.98, and 48.27 mM *p*-CA. Bar: PCA, chartreuse; 4-HBA, grey; *p*-CA, unfilled. **i** PCA and VA production from FA by Spca1–Spca7 fed with 43.48, 43.20, 43.35, 43.72, 43.30, 43.72, and 42.82 mM FA. Bar: PCA, pink; VA, grey; FA, unfilled. FA ferulic acid, *p*-CA *p*-coumaric acid, CaA caffeic acid, PCA protocatechuic acid, 4-HBA *p*-hydroxybenzoate, VA vanillic acid. Values are presented as mean ± s.d. (*n* = 3 independent biological replicates). Source data are provided as a Source Data file.

The biological funneling pathways of *C. glutamicum* are generally defined by upper and lower pathways. To elucidate metabolic limitations in the native biological funneling pathway of *C. glutamicum*, in this study, we first delineated the upper biological funneling pathway, which channels phenylpropanoids toward PCA, into two functional layers on the basis of metabolic roles. The first layer (Layer 1) involves β-oxidation of phenylpropanoids, yielding benzoate derivatives and acetyl-CoA, whereas the second layer (Layer 2) comprises the hydroxylation of *p*-hydroxybenzoic acid (4-HBA) and O-demethylation of vanillic acid (VA). These two layers ultimately converge into central carbon metabolism by ring-opening reactions^57,58^ and the β-ketoadipate pathway^59^ (Fig. 2a). To experimentally assess pathway performance, we disrupted *pcaHG* (encoding PCA-3,4-dioxygenase) in wild-type *C. glutamicum* ATCC 13032 to block PCA downstream metabolism, generating strain Spca1 (Table 1). During fed-batch fermentation with three phenylpropanoids CaA, *p*-CA, and FA as substrates, strain Spca1 accumulated PCA at concentrations of 20.52 mM, 12.31 mM, and 7.79 mM, corresponding to molar yields of 38.49, 29.38, and 19.18% (mol/mol), respectively (Fig. 2b–d). Significant accumulation of intermediates (12.3 mM 4-HBA and 8.9 mM VA) was observed during *p*-CA and FA metabolism.

**Table 1:** Selected strains used in this study

Strain	Characteristics	Reference
*C. glutamicum* ATCC 13032	Wild-type strain	Laboratory stock
Spca1	*C. glutamicum* ATCC 13032 with *pcaHG* gene deletion	This study
Spca2	Spca1 carrying pEC-XK99E-P_trc_*-phdA-phdE-phdB-phdC*	This study
Spca3	Spca1 with *phdR* gene deletion	This study
Spca4	Spca2 with *phdR* gene deletion	This study
Spca5	Spca4 carrying pEC-XK99E-P_trc_-*vanAB*^*Cgl*^	This study
Spca6	Spca4 with *vanR* gene deletion	This study
Spca7	Spca6 carrying pEC-XK99E-P_trc_-*vanAB*^*Cgl*^	This study
Spca8	Spca4 carrying pEC-XK99E-P_trc_-*pobA*^*Cgl*^	This study
STALE9	Evolved strain-3 derived from ALE with *p*-CA	This study
SHT1	STALE9 with *pcaHG* gene deletion	This study
SASR1	SHT1 with P_*phdB*_ replcaced by P_bba-23110_-O2-B0034	This study
SASR2	SHT1 with P_*phdB*_ replcaced by P_bba-23106_-O2-B0034	This study
SASR3	SHT1 with P_*phdB*_ replcaced by P_bba-23119_-O2-B0034	This study
SASR4	SASR3 with P_*pobA*_ replaced by P_pobA_-QF-_*QUAS*_-P_bba-23110_	This study
SASR5	SASR3 with P_*pobA*_ replaced by P_pobA_-QF-_*QUAS*_-P_bba-23106_	This study
SASR6	SASR3 with P_*pobA*_ replaced by P_pobA_-QF-_*QUAS*_-P_bba-23119_	This study
SASR7	SASR6 with P_*vanA*_ replaced by P_Bba-23110_-O_van_	This study
SASR8	SASR6 with P_*vanA*_ replaced by P_Bba-23106_-O_van_	This study
SASR9	SASR6 with P_*vanA*_ replaced by P_Bba-23119_-O_van_	This study
SASR7-C1	SASR7 with *catBC* deletion and genomic intergration of P_bba-23119_-*aroY-ecdB-ecdD*	This study
SASR7-K19	SASR7 with *pcaIJ* deletion and genomic intergration of P_Bba-23119_-*pcaHG*	This study

This accumulation indicates that Layer 2 acts as the rate-limiting step in the native funneling pathway, which is governed primarily by 4-hydroxybenzoate-3-hydroxylase (PobA) and vanillate O-demethylase (VanAB). To address this bottleneck, we screened *pobA*^28,60^ and *vanAB*^21,61,62^ from multiple sources and expressed them in Spca1. The native homologous enzymes PobA^*cgl*^ and VanAB^*cgl*^ outperformed their heterologous counterparts in intermediate consumption, significantly reducing 4-HBA and VA accumulation; therefore, these enzymes were selected for subsequent pathway optimization (Fig. 2e, f and Supplementary Table 1).

To further increase flux through Layer 1, we overexpressed the *phd* gene cluster (*phdAEBC*) under the P_trc_ promoter using the plasmid pEC-XK99E, generating strain Spca2. This modification improved the conversion of CaA but led to pronounced accumulation of the intermediates 4-HBA and VA during the metabolism of *p*-CA and FA (Fig. 2g–i), indicating an exacerbation of downstream bottlenecks. To relieve potential transcriptional repression of the *phd* cluster, we deleted the gene *phdR*, which encodes its regulator, generating Spca3 and Spca4. While this strategy increased PCA production from CaA to more than 25 mM (yield >50%), it failed to increase PCA synthesis from *p*-CA or FA. Instead, substantial accumulation of intermediates 4-HBA and VA persisted (e.g., 30.5 mM 4-HBA and 21.0 mM VA in Spca4), confirming that while strengthening Layer 1 improved the upstream flux, Layer 2 remained the dominant rate-limiting step.

To overcome this limitation, we next engineered Layer 2 for improved conversion of 4-HBA and VA to PCA. Overexpression of *pobA*^*Cgl*^ in Spca4 (strain Spca8) enhanced 4-HBA-to-PCA conversion, achieving 15.50 mM PCA with a yield of 32.11% from *p*-CA. For VA-to-PCA conversion, three strategies were evaluated: overexpression of *vanAB*^*Cgl*^ (Spca5), deletion of the gene *vanR* that encodes repressor (Spca6), and their combination (Spca7). All the strategies improved PCA production from FA, with Spca6 showing the highest titer and yield (19.02 mM and 43.50%, respectively) (Fig. 2g–i).

Despite these efforts, conventional metabolic engineering of the native funneling pathway resulted in only modest improvements, with a maximum PCA titer of 25.19 mM and a yield of 52.83% (mol/mol). This limited performance is primarily attributed to two factors: (i) substrate toxicity, as evidenced by the suppressed growth even at substrate concentrations below 20 mM (Supplementary Fig. 1), and (ii) severe intermediate accumulation arising from the persistent imbalance between Layer 1 and Layer 2. These limitations motivated us to pursue a combined strategy integrating adaptive laboratory evolution for tolerance enhancement with biosensor-driven autonomous dynamic metabolic control.

### Adaptive laboratory evolution improves *C. glutamicum*’s tolerance to lignin-related phenylpropanoids

**Fig. 3:**
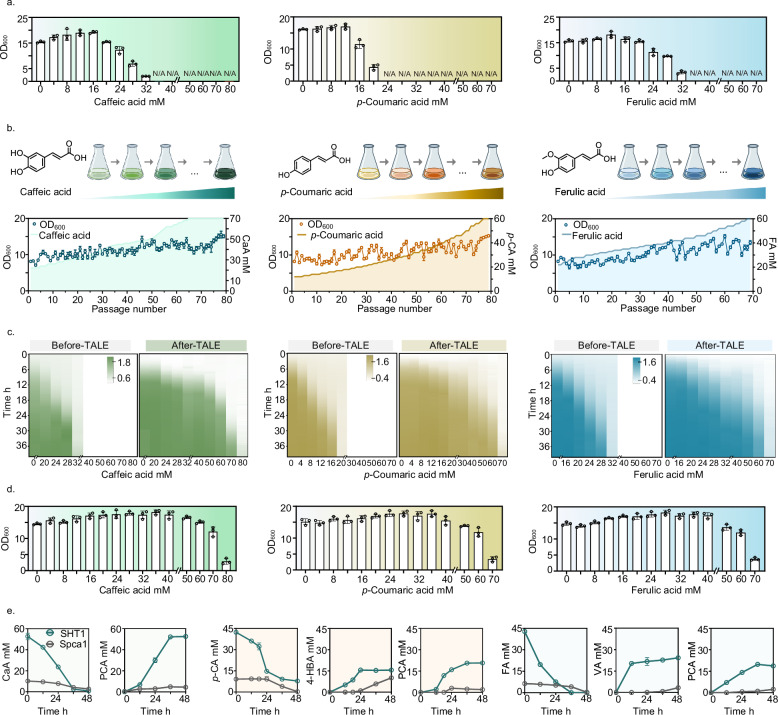
Adaptive laboratory evolution of *C. glutamicum* for enhanced tolerance and utilization of phenylpropanoids. **a** Growth (OD_600_) of native *C. glutamicum* under different concentrations of CaA, *p*-CA, and FA. **b** Adaptive Laboratory Evolution (ALE) process for phenylpropanoids (CaA, *p*-CA, FA). Each circle denotes OD_600_ at subculturing. **c** Growth curves of wild-type *C. glutamicum* and ALE-evolved strains under representative inhibitory concentrations of CaA, p-CA, and FA. **d** Growth (OD_600_) of evolved strains under different concentrations of CaA, *p*-CA, and FA. **e** Comparison of CaA, *p*-CA, and FA degradation abilities of strain SHT1 and Spca1, temporal concentration profiles of substrates, intermediates, and products are shown. FA ferulic acid, *p*-CA *p*-coumaric acid, CaA caffeic acid, PCA protocatechuic acid, 4-HBA *p*-hydroxybenzoate, VA vanillic acid. Values are presented as mean ± s.d. (*n* = 3 independent biological replicates) in (**a**, **b**, **d**, **e**), and as mean ± s.d. (*n* = 2 independent biological replicates) in (**c**). Source data are provided as a Source Data file.

To overcome substrate toxicity and enhance metabolic performance, we applied adaptive laboratory evolution (ALE) to *C. glutamicum*, yielding strains with markedly improved tolerance to lignin-related phenylpropanoids. While the wild-type strain exhibited severe growth inhibition at relatively low substrate concentrations (32 mM for CaA, 20 mM for *p*-CA, and 32 mM for FA) (Fig. 3a), evolved populations tolerated substantially higher levels, up to 80 mM CaA and 70 mM *p*-CA or FA (Fig. 3b–d), and maintained robust growth under conditions that completely inhibited the parental strain.

To characterize evolutionary outcomes, three independent clones were isolated from each ALE lineage, generating strains STALE1–3 (CaA), STALE4–6 (FA), and STALE7–9 (*p*-CA). Whole-genome resequencing revealed a high degree of mutational convergence across strains that evolved on different substrates, with ~86% of coding-region mutations shared (Supplementary Fig. 2 and Supplementary Data 1), suggesting that convergent adaptive mechanisms were driven by the structural similarity of phenylpropanoids. Consistent with this, all evolved strains exhibited cross-tolerance to CaA, *p*-CA, and FA, with STALE9 displaying the highest tolerance, achieving the highest cell density and sustained growth under otherwise inhibitory conditions (Supplementary Fig. 3).

To assess the impact of enhanced tolerance on metabolic performance, we disrupted *pcaHG* in STALE9 to generate strain SHT1. Compared with Spca1, SHT1 exhibited significantly increased catabolic rates for CaA, *p*-CA, and FA (1.10, 0.92, and 1.19 mmol/L/h versus 0.57, 0.39, and 0.28 mmol/L/h, respectively). Similarly, SHT1 produced 52.5, 20.7, and 19.3 mM PCA within 36 h, achieving conversion yields of 99.62, 48.94, and 45.10% from CaA, *p*-CA, and FA, respectively. Both the PCA titer and the conversion efficiency exceeded those of all the previously engineered strains. SHT1 tolerated single-batch substrate loads equivalent to the total five-batch feeding pre-evolution (Fig. 3e), demonstrating enhanced toxicity resistance and metabolic robustness. These findings confirm that ALE is an effective strategy for alleviating toxicity constraints and improving pathway performance.

### Elucidation of synergistic tolerance mechanisms in evolved *C. glutamicum*

**Fig. 4:**
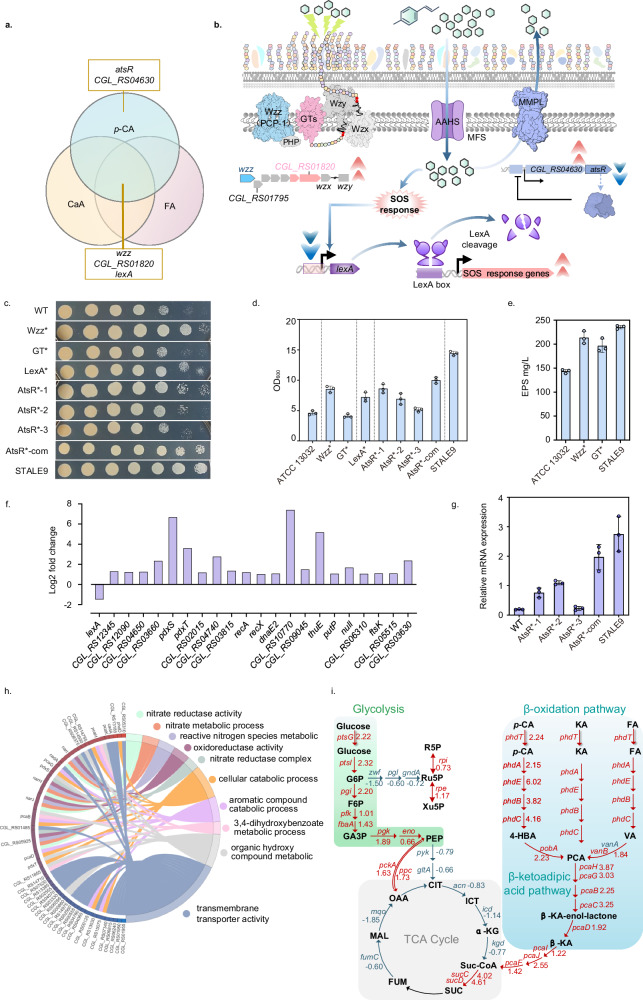
Multi-omics analysis and experimental validation of the mechanisms for improved aromatic tolerance. **a** Shared and *p*-CA-specific mutations in evolved strains. Colored circles indicate mutations in biological replicates evolved with *p*-CA (yellow), FA (lilac), or CaA (blue). **b** Proposed phenylpropanoids tolerance mechanism in *C. glutamicum*. **c**, **d** Functional validation via growth rescue of mutant strains on plates with 20 mM *p*-CA (**c**); and in CGXII liquid medium (**d**). **e** Exopolysaccharide (EPS) quantification of Wzz*, GT*, STALE9, and wild-type *C. glutamicum*. **f** Differential expression of SOS response-related genes in STALE9 versus wild-type under *p*-CA stress. Genes are annotated via Gene Ontology analysis. **g** qRT-PCR analysis of *CGL_RS04630* expression in AtsR*-1, AtsR*-2, AtsR*-3, and AtsR*-com. **h** Top 10 enriched GO terms of differentially expressed genes between STALE9 and native *C. glutamicum*. **i** Transcriptional changes in central carbon metabolism and phenylpropanoid pathways of STALE9 vs. native *C. glutamicum*. |log₂FC| ≥0.6 indicates significant difference. G6P Glucose-6-phosphate, F6P Fructose-6-phosphate, GA3P Glyceraldehyde-3-phosphate, PEP Phosphoenolpyruvate, OAA Oxaloacetate, Acetyl CoA Acetoacetyl coenzyme A, CIT Citrate, ICT Isocitrate, αKG Alpha-ketoglutarate, Suc-CoA Succinyl coenzyme A, SUC Succinic acid, FUM Fumarate, Mal Malate, Xu5P Xylulose-5-phosphate, R5P Ribose-5-phosphate, TCA Tricarboxylic acid cycle, β-KA-enol-lactone β-ketoadipic enol lactone, β-KA β-ketoadipic acid, *ptsG* encodes glucose transporters; *ptsI* encodes phosphoenolpyruvate-protein phosphotransferase; *pgi* encodes phosphoglucose isomerase; *pfk* encodes phosphofructokinase 1; *fbaA* encodes class II fructose-bisphosphate aldolase; *pgk* encodes phosphoglycerate kinase; *eno* encodes phosphopyruvate hydratase; *pyk* encodes pyruvate kinase; *gltA* encodes citrate synthase; *pckA* encodes phosphoenolpyruvate carboxykinase; *acn* encodes aconitate hydratase; *icd* encodes NADP-dependent isocitrate dehydrogenase; *kgd* encodes α-ketoglutarate dehydrogenase; *sucC* encodes succinate-CoA ligase subunit beta; *sucD* encodes succinate-CoA ligase subunit alpha; *fumC* encodes class II fumarate hydratase; *mqo* encodes malate dehydrogenase; *phdT* encodes MHS family transporter; *phdA* encodes acyl-CoA ligase; *phdE* encodes enoyl-CoA hydratase; *phdB* encodes 3-hydroxyacyl-CoA dehydrogenase; *phdC* encodes 3-oxoacyl-CoA ketohydrolase; *pobA* encodes 4-hydroxybenzoate-3-hydroxylase; *vanAB* encodes vanillate O-demethylase; *pcaHG* encodes protocatechuate-3,4-dioxygenase; *pcaB* encodes β-carboxy-*cis,cis*-muconate lactonizing enzyme; *pcaC* encodes γ-carboxymuconolactone decarboxylase; *pcaD* encodes β-ketoadipate enol-lactone hydrolase; *pcaIJ* encodes β-ketoadipate succinyl-CoA transferase; *pcaF* encodes β-ketoadipyl-CoA thiolase. Values are presented as mean ± s.d. (*n* = 3 independent biological replicates). Source data are provided as a Source Data file.

To elucidate the genetic basis of enhanced phenylpropanoid tolerance in STALE9, we combined multi-omics analysis with reverse genetic validation. The identification of convergent mutations across evolved strains revealed three major functional modules underlying tolerance: (i) cell envelope remodeling via polysaccharide biosynthesis, (ii) global stress response activation, and (iii) enhanced detoxification through efflux transport systems (Fig. 4a).

First, mutations affecting polysaccharide biosynthesis were found to improve tolerance via cell envelope reinforcement. Introduction of the STALE9-derived *wzz* mutation gene (Wzz*, E363D) into the wild type significantly increased exopolysaccharide (EPS) secretion^63–65^ and enhanced phenylpropanoid tolerance (Fig. 4b–e), indicating that elevated EPS production contributes to toxin resistance^63,66^. In contrast, mutation of the glycosyltransferase gene *CGL_RS0182051* (GT*) did not result in measurable phenotypic changes, suggesting functional redundancy in polysaccharide biosynthesis pathways (Fig. 4c–e and Supplementary Table 2).

Second, mutations associated with the regulation of the SOS response further contributed to tolerance. Regulated by the master repressor LexA, the SOS system consists of a DNA damage-inducible regulon that mediates repair, homologous recombination, and translesion synthesis in response to environmental stressors^67^. Introduction of the STALE9-derived promoter mutation in *lexA* (LexA*) improved growth under *p*-CA stress (Fig. 4c, d). Transcriptomic analysis confirmed the downregulation of *lexA* and derepression of SOS-response genes involved in DNA repair and stress adaptation^68^ (Fig. 4f), indicating constitutive activation of the SOS regulon and enhanced stress resilience.

Third, mutations enhancing efflux-mediated detoxification were identified in the operon encoding the MMPL-family efflux pump (*CGL_RS04630*) and its repressor AtsR^69^. Functional analysis revealed that mutations in the AtsR binding site (AtsR*-1) and promoter region (AtsR*-2) significantly increased transporter expression and improved growth under phenylpropanoid stress, whereas a coding-region insertion in AtsR (AtsR*-3) had no effect. The combined mutant (AtsR*-com) exhibited synergistic increases in both growth and transporter expression (Fig. 4c, d, g), demonstrating that derepression of this efflux system facilitates the detoxification of phenylpropanoids.

To further link these genetic changes to global metabolic adaptation, transcriptomic analysis of STALE9 under *p*-CA stress revealed widespread transcriptional reprogramming, with 917 genes differentially expressed relative to those of the wild type (Supplementary Fig. 4a). Genes involved in aromatic compound transport (*CGL_RS05310*, *pcaK*, *CGL_RS01485*, and *CGL_RS06375*) and degradation (including *phdD*, *pcaH*, *pcaG*, *pcaB*, and *pcaD*), along with key glycolytic genes, were significantly upregulated (Fig. 4h, i). In contrast to the repression observed in the wild type (Supplementary Fig. 4b), these pathways remained active in STALE9, indicating sustained substrate uptake, degradation, and central carbon metabolism under stress conditions.

Collectively, these results demonstrate that ALE enables synergistic enhancement of tolerance through coordinated activation of polysaccharide-mediated protection, stress response systems, and efflux-driven detoxification, thereby supporting robust growth and metabolic activity under high phenylpropanoid concentrations.

### Development of lignin-related aromatic-responsive systems in *C. glutamicum*

While ALE significantly enhances the cellular tolerance and metabolic capacity for lignin monomers, it does not inherently enable precise control over metabolic flux toward desired products. As a result, evolved strains still exhibit imbalanced flux distribution and intermediate accumulation during phenylpropanoid metabolism, necessitating dynamic regulatory strategies to improve product yield and carbon efficiency.

To address this limitation, we developed a layered, multi-input autonomous dynamic control system to enable coordinated regulation of the biological funneling pathway. Central to this system is a panel of metabolite-responsive biosensors derived from native aromatic regulatory networks in *C. glutamicum*. We engineered six key aTF-based biosensors that respond to different pathway metabolites, including PhdR (phenylpropanoids), PcaO (4-HBA), GenR (gentisate), BenR (benzoate), RolR (resorcinol), and IhtR (alternative resorcinol pathway), thereby establishing a modular sensing framework covering multiple nodes of the funneling pathway.

**Fig. 5:**
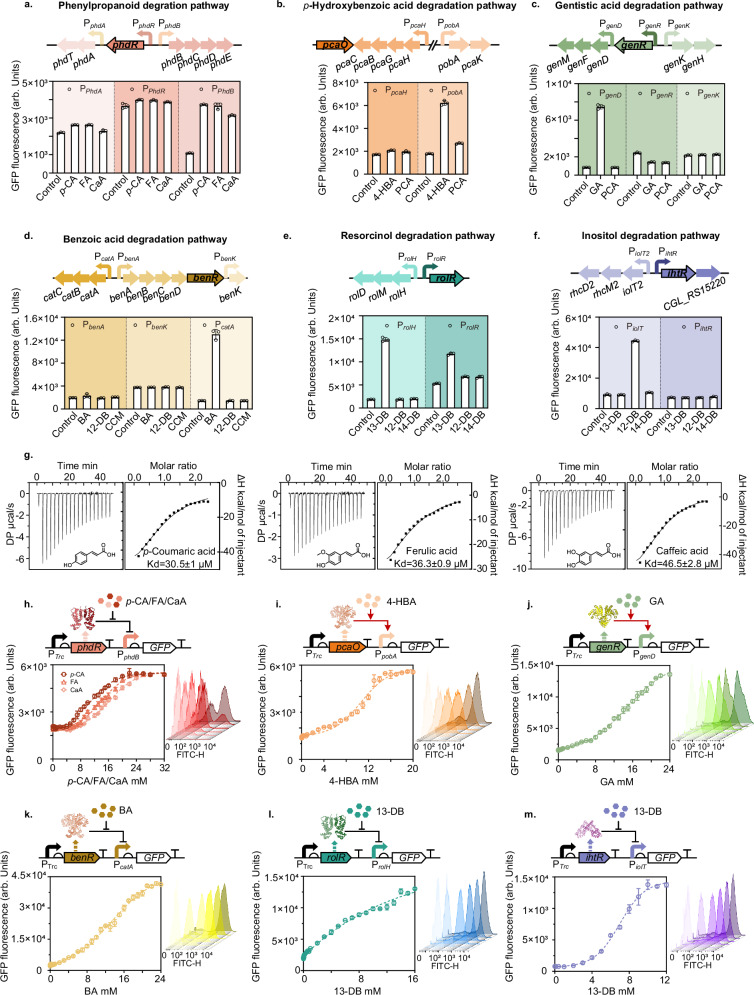
Development of lignin-related aromatics-responsive biosensors based on aTF-binding promoter and ligand identification. Identification of aTF-binding promoter regions (each transcriptional unit within gene clusters) and optimal binding ligands (selected from pathway substrates, intermediates, and structurally analogous compounds) for the phenylpropanoid catabolic pathway (**a**), 4-hydroxybenzoate catabolic pathway (**b**), gentisate pathway (**c**), benzoate catabolic pathway (**d**), and resorcinol metabolic pathways (**e**, **f**). *p*-CA *p*-coumaric acid, FA ferulic acid, CaA caffeic acid, 4-HBA *p*-Hydroxybenzoate, PCA protocatechuic acid, GA gentisic acid, BA benzoic acid, 12-DB catechol, 13-DB resorcinol; 14-DB, hydroquinone, CCM *cis,cis*-muconic acid. Dynamic range refers to the fold change of maximum fluorescence intensity divided by the basal fluorescence intensity. **g** PhdR affinity measurements for *p*-CA, FA, and CaA. 1.05 g L^−1^ PhdR was used for titration with 0.125 g L^−1^ *p*-CA, 0.77 g L^−1^ PhdR was used for titration with 0.115 g L^−1^ FA, and 1.72 g L^−1^ PhdR was used for titration with 0.23 g L^−1^ CaA. Binding isotherms was fitted using the One Set of Sites model. Dose-dependent fluorescence response curves and flow cytometry analysis of PhdR-*p*-CA-P_phdB_ (**h**), PcaO-4-HBA-P_pobA_ (**i**), GenR-GA-P_genD_ (**j**), BenR- BA-P_catA_ (**k**), RolR−1,3-DB-P_rolH_ (**l**), and IhtR-1,3-DB-P_iolT_ (**m**). 1 × 10^5^ cells were detected during flow cytometry analysis. Operational range refers to the interval where the output signal induced by ligand concentration exceeds the background threshold until it reaches saturation. Values are presented as mean ± s.d. (*n* = 3 independent biological replicates). Source data are provided as a Source Data file.

To identify the optimal responsive promoter, we systematically screened combinations of candidate ligands (substrates, intermediates, and structural analogs) and cognate promoters using GFP as a reporter. This enabled the identification of optimal aTF–ligand–promoter pairs for each system. For example, the PhdR-based biosensor exhibited the strongest response with the promoter P_*phdB*_, particularly in the presence of *p*-CA, which induced the greatest activation fold change (Fig. 5a). Similarly, optimal regulatory combinations were identified for the remaining five systems, including PcaO-4-HBA-P_*pobA*_, GenR-gentisic acid (GA)-P_*genD*_, BenR-benzoate (BA)-P_*catA*_, RolR-resorcinol (1,3-DB)-P_*rolH*_, and IhtR- 1,3-DB-P_*iolT*_ (Fig. 5b–f).

Biophysical characterization using isothermal titration calorimetry (ITC) further confirmed that PhdR functions as a central regulator of the upper funnel pathway, with binding affinities (*K*_d_) of 30.5 ± 1 μM (*p*-CA), 36.3 ± 0.9 μM (FA), and 46.5 ± 2.8 μM (CaA) (Fig. 5g and Supplementary Fig. 5). The dynamic range and operational range of the ligand dose-dependent induction were validated at single-cell resolution by flow cytometry (Fig. 5h–m). Functional characterization in *E. coli* revealed that PhdR, BenR, RolR, and IhtR act as repressors, whereas PcaO and GenR function as activators (Supplementary Fig. 6). For the regulators PhdR and IhtR (the other were derived from the literature^70–73^), DNase I footprinting identified their operator binding sites, further elucidating their regulatory mechanisms (Supplementary Fig. 7 and Supplementary Table 3). Together, these results establish versatile and modular biosensor toolkits that enable multi-input sensing and lay the foundation for the dynamic, autonomous control of lignin-related aromatic metabolism.

### Design and optimization of the layered and multi-input system

**Fig. 6:**
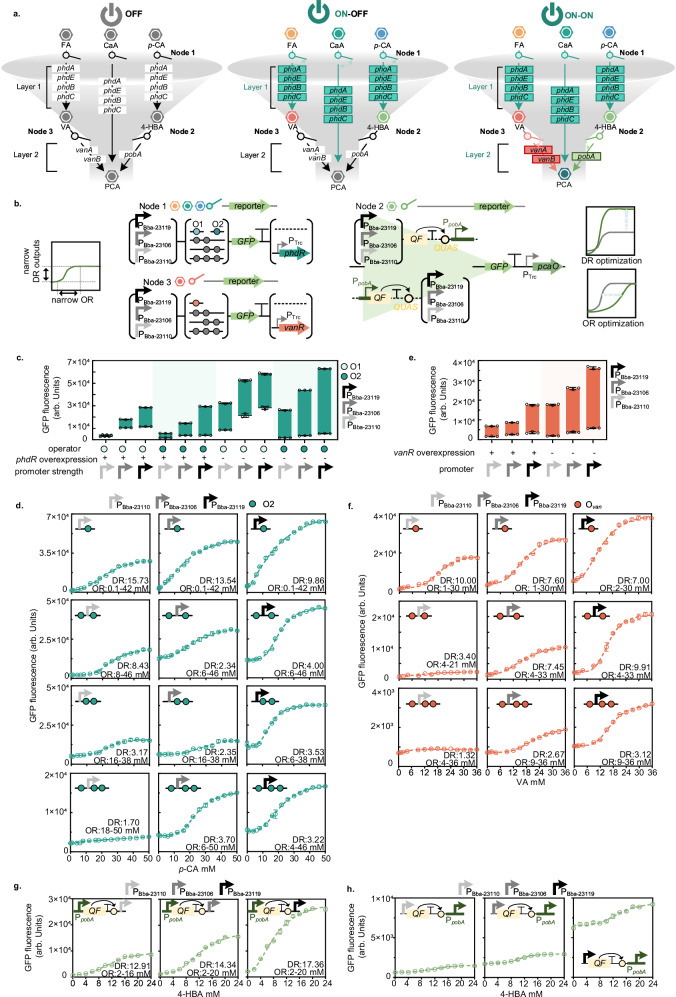
Engineering and optimization of regulatory elements for the layered and multi-input autonomous biological funneling system. **a** Schematic of the layered and multi-input autonomous dynamic control system. This system comprising dual-layer pathways and three nodes. In the absence of substrates, system remains in the “OFF” state. Upon substrates introduction, Layer 1 pathway activates (ON state), while insufficient intermediate accumulation keeps Layer 2 pathway inactive (OFF state), resulting in an ON-OFF hybrid state. Subsequent intermediate accumulation triggers Layer 2 activation, achieving full ON-ON state. **b** Signal modulation optimization framework for biosensors, comprising synthetic promoter library construction and signal amplification circuitry. **c** The regulatory effects of operators (O1, O2) and *phdR* overexpression. Bar charts show biosensor dynamic range, with lower and upper bounds representing OD₆₀₀-normalized fluorescence without or with 30 mM *p*-CA, respectively. Light and dark circles indicate O1 and O2; “+” and “−” indicate plasmid-based *phdR* overexpression or genomic *phdR* expression; arrows denote promoters of different strengths (P_Bba−23110_, P_Bba−23106_, and P_Bba−23119_). **d** Dose-response curve of the PhdR-controlled hybrid promoter library. **e** The regulatory effects of *vanR* over-expression. Bar charts show biosensor dynamic range, with lower and upper bounds representing OD₆₀₀-normalized fluorescence without or with 25 mM VA. “+” and “−” indicate plasmid-based *vanR* overexpression or genomic *vanR* expression. Arrows with increasing shading intensity represent promoters of varying strengths (P_Bba−23110_, P_Bba−23106_, and P_Bba−23119_). **f** Dose-response curve of the VanR-controlled hybrid promoter library. **g**, **h** QF-mediated amplification circuit for PcaO. Operational range (OR) refers to the interval where the output signal induced by ligand concentration exceeds the background threshold until it reaches saturation. Dynamic range (DR) refers to the fold change of maximum fluorescence intensity divided by the basal fluorescence intensity. FA ferulic acid, *p*-CA *p*-coumaric acid, CaA caffeic acid, PCA protocatechuic acid, 4-HBA *p*-hydroxybenzoate, VA vanillic acid. Values are presented as mean ± s.d. (*n* = 3 independent biological replicates). Source data are provided as a Source Data file.

To enable precise and adaptive regulation of the biological funneling pathway, we designed a layered, multi-input autonomous dynamic control system based on a two-layer cascade architecture. In this system, phenylpropanoid substrates (CaA, *p*-CA, and FA) activate Layer 1 gene expression, generating intermediates such as 4-HBA and VA, which subsequently induce the expression of Layer 2 genes (*pobA* and *vanAB*). This hierarchical, demand-driven regulation establishes a positive feedback loop that balances metabolic flux and minimizes intermediate accumulation (Fig. 6a).

The system is governed by three core regulatory nodes—PhdR, PcaO, and VanR—corresponding to substrate and intermediate sensing modules (PhdR-phenylpropanoids -P_*phdB*_, PcaO-4-HBA-P_*pobA*_, and VanR-VA-P_*vanA*_, respectively). Initial characterization revealed that these nodes exhibited limited dynamic ranges (2.9, 4.1, and 2.0, respectively) and narrow operational ranges (5–22, 1–15, and 4–20 mM), limiting their effectiveness for metabolic control (Fig. 5h, i and Supplementary Fig. 8a). Its three core nodes initially suffer from limited responsiveness. To enhance their performance, we developed a signal modulation optimization framework based on engineered hybrid promoters with effective operators (Fig. 6b).

For PhdR, analysis of operator architecture (O1, O2) revealed that positioning the O2 operator downstream of the −10 region significantly enhanced responsiveness (Fig. 6c). Genomic expression of *phdR* provided sufficient repression to maintain low basal levels; thus, plasmid overexpression was not needed. On the basis of this insight, we constructed a combinatorial hybrid promoter library by varying the promoter strength and operator copy number. Compared with the wild-type P_*phdB*_, the optimal promoter variants increased the dynamic range to a maximum of 15.73 and expanded the operational range to 0.1–42 mM (Fig. 6d). Increasing the O2 operator copy number leads to decreased expression strength ranges, providing a tunable output control strategy. These optimized promoters maintain responsiveness across multiple substrates, including *p*-CA, FA, and CaA (Supplementary Fig. 8b, c).

For VanR, a similar hybrid promoter engineering strategy was applied. Optimization of the promoter architecture increased the dynamic range to 10.00 and expanded the operational range to 3–30 mM (Fig. 6e, f), indicating improved responsiveness of the VA-sensing module.

In contrast, the PcaO node exhibited limited activation capacity and strict operator positioning constraints, rendering promoter engineering insufficient (Supplementary Fig. 8d). To address this, we implemented a QF-mediated transcriptional amplification circuit based on the orthogonal QF/QUAS system, where QF, a transcriptional activator derived from *Neurospora crassa*, enables signal amplification by driving downstream gene expression^74^. By coupling P_*pobA*_-driven QF expression with QUAS site-containing promoters, we significantly enhanced the system’s responsiveness, achieving a dynamic range of 17.36 and an operational range of 2–20 mM (Fig. 6g, h). This systematic optimization rendered all nodes suitable for precise metabolic control.

### Biological funneling reprogramming using a layered multi-input autonomous system

**Fig. 7:**
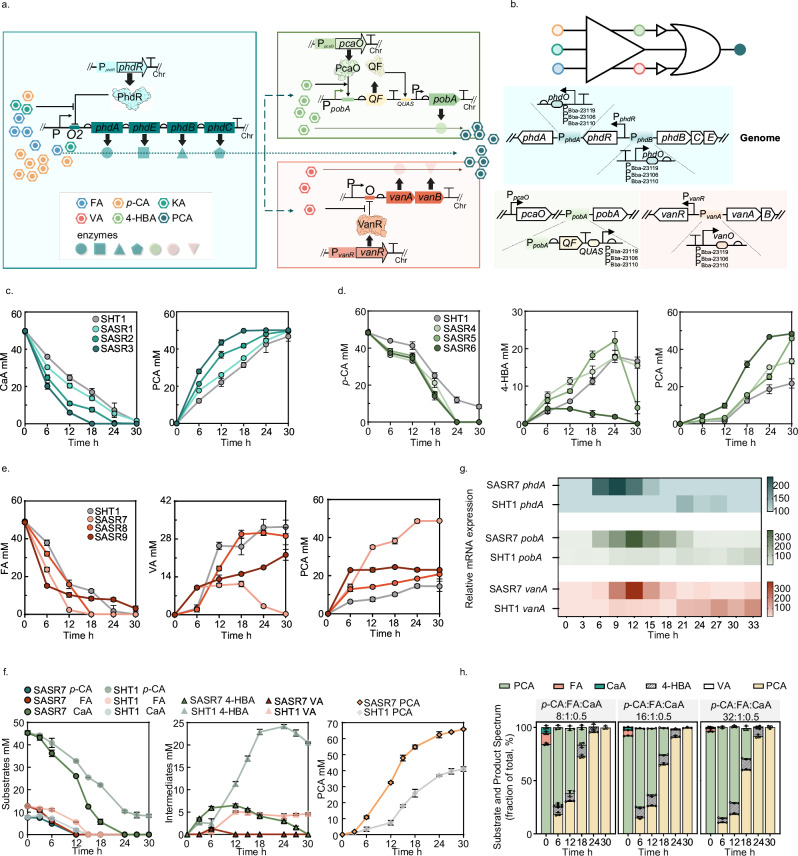
Construction, fine-tuning, and fermentation performance of the layered and multi-input autonomous biological funneling system. **a** Regulatory schematic of the layered and multi-input autonomous biological funneling where solid lines indicate functional regulations of small-molecule with regulators and dashed lines depict compound metabolic fluxes. **b** Logic circuit diagram and genomic engineering strategy for the layered and multi-input autonomous biological funneling pathway. **c**–**e** Fermentation of strains engineered through key-node modifications and fine-turning of Node1 (**c**), Node2 (**d**), and Node3 (**e**). **f** Mixed phenylpropanoid fermentation performance using the fine-tuned layered and multi-input autonomous biological funneling, with dark traces representing the engineered SASR7 strain and light traces indicating control strain SHT1. **g** Time-course RT-qPCR analysis of SASR7 during fermentation with complex lignin-related mixtures. **h** Fermentation profiles of strain SASA7 under varying phenylpropanoid substrate ratios. Stacked bars show normalized fractions of substrates (*p*-CA, FA, CaA), intermediates (4-HBA, VA), and product (PCA) at indicated time points (all concentrations normalized to total substrate input; sum = 100%). Three feeding conditions (total mM): 63.21 (53.21 *p*-CA, 6.57 FA, 3.42 CaA), 62.69 (57.71 *p*-CA, 3.42 FA, 1.56 CaA), and 62.00 (59.44 *p*-CA, 1.82 FA, 0.73 CaA). Colors are defined in the legend. FA ferulic acid, *p*-CA *p*-coumaric acid, CaA caffeic acid, PCA protocatechuic acid, 4-HBA *p*-hydroxybenzoate, VA vanillic acid. Values are presented as mean ± s.d. (*n* = 3 independent biological replicates). Source data are provided as a Source Data file.

To evaluate the performance of the layered and multi-input autonomous dynamic control system, we employed PCA as a model output, given its central role in aromatic metabolism and pharmacological values^75,76^ (Fig. 7a).

We first implemented hierarchical tuning of the three regulatory nodes to achieve precise and adaptive control of the funneling pathway. For Layer 1, the native promoters of the *phdAEBC* cluster were replaced with synthetic PhdR-regulated variants of graded strength (P_Bba-23110_-O2, P_Bba-23106_-O2, and P_Bba-23119_-O2), generating strains SASR1–3. This enabled substrate-responsive modulation of the upstream flux to varying degrees. Among them, the strongest variant, SASR3 exhibited optimal performance, achieving near-complete conversion of 50 mM CaA to PCA (99.96% yield) within 18 h (Fig. 7b, c). We next tuned Layer 2 by introducing PcaO-based transcriptional cascade amplification circuits of increasing strength (P_Bba-23110_, P_Bba-23106_, and P_Bba-23119_) to regulate *pobA* expression (SASR4–6). The strongest variant, SASR6, efficiently converted 48.38 mM *p*-CA to 48.37 mM PCA within 30 h, achieving a yield of 99.99% while maintaining intermediate 4-HBA levels below 5 mM (Fig. 7b, d), indicating effective suppression of metabolic imbalance.

Finally, the downstream node in Layer 2 controlling *vanAB* expression was optimized using VanR-regulated promoters of graded strength, yielding strains SASR7–9. The optimized strain SASR7 completely consumed 48.67 mM FA within 18 h and converted it to 48.66 mM PCA by 24 h, achieving a yield of 99.98% (Fig. 7b, e). Additionally, control experiments using glucose as the sole carbon source revealed negligible PCA production, confirming that the observed product formation originated from lignin-related aromatics rather than background synthesis from glucose (Supplementary Fig. 9).

Having established a fully optimized biological funneling system, we evaluated its performance using mixed lignin-related phenylpropanoids. A substrate mixture ((*p*-CA:FA:CaA = 4:1:0.5; total 65.93 mM), mimicking lignin-rich liquor derived from the alkaline pretreatment of corn stover, was completely converted to PCA by SASR7 within 30 h. The strain produced 65.86 mM PCA at a yield of 99.89%, with intermediate levels remaining below 5 mM, significantly outperforming the control strain SHT1 (62.04% yield) (Fig. 7f). Time-course RT‒qPCR analysis revealed coordinated, dynamic transcriptional regulation underlying this effect. The upstream *phdAEBC* cluster was rapidly induced, peaking at 9 h and declining to baseline by 18 h as substrates were depleted, whereas the downstream genes *pobA* and *vanAB* exhibited delayed and sustained activation, peaking at 12 h. We observed that *vanAB* expression decreased (after 18 h) earlier than *pobA* expression (remaining active until 21 h), which is consistent with the lower FA abundance in the substrate mixture (Fig. 7g). This temporal regulation confirms that the system autonomously balances metabolic flux and conserves cellular resources through feedback-controlled gene expression.

Layered, multi-input autonomous biological funneling in SASR7 markedly outperformed native funneling in SHT1 during phenylpropanoid conversion. To further benchmark this multiplexed dynamic control strategy against conventional approaches, we constructed strain SHTCE, in which all the funneling genes (*phdAEBC*, *vanAB*, and *pobA*) were constitutively overexpressed. Although SHTCE retained the baseline conversion capacity of SHT1, its overall performance remained inferior to that of SASR7 (Supplementary Fig. 10), demonstrating that static overexpression is insufficient to achieve efficient flux coordination. These results highlight the advantage of the layered and multi-input autonomous dynamic control system in achieving balanced and efficient metabolic conversion.

Given that biomass-derived feedstocks are inherently complex, variable, and heterogeneous, we next evaluated the compositional adaptability of SASR7. Specifically, we tested its performance across a range of *p*-CA/FA/CaA ratios to simulate varying substrate compositions. SASR7 consistently maintained the conversion efficiency across all the tested conditions (Fig. 7h), demonstrating its strong robustness and ability to adapt to compositional fluctuations in lignin-derived feedstocks.

### Establishment of a robust platform for bioplastic precursor production

**Fig. 8:**
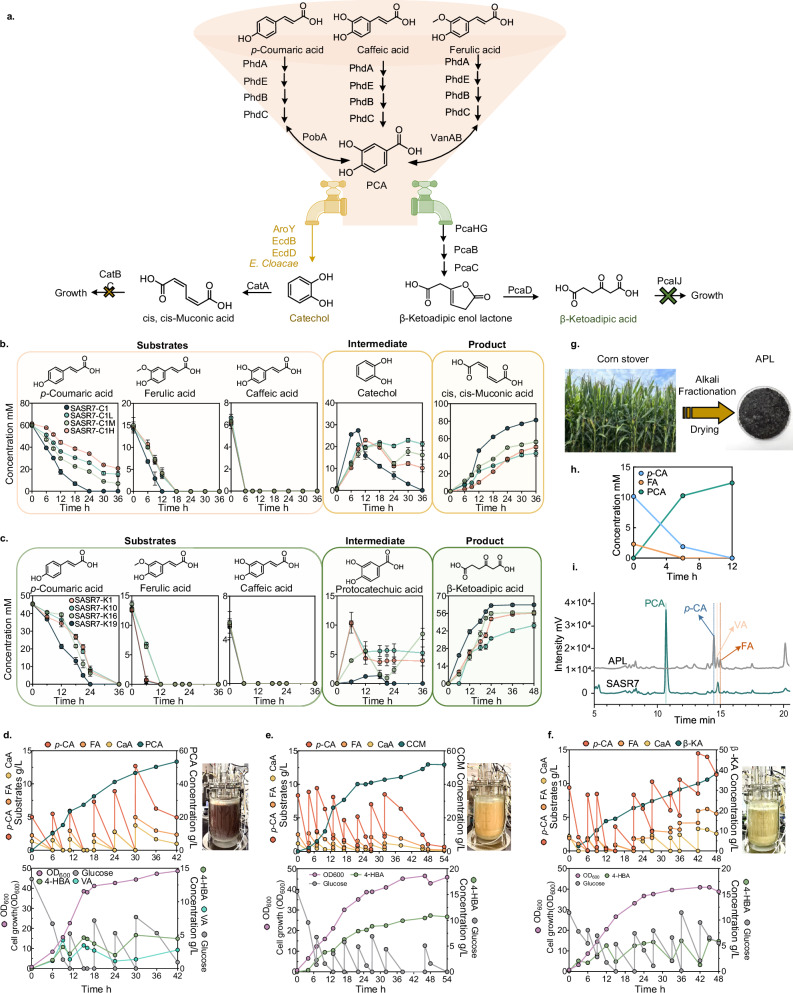
Versatility and scaled-up application of the autonomous biological funneling system for diverse high-value chemicals and lignin-derived feedstocks. **a** Pathways for producing high-value chemicals PCA, CCM, and β-KA based on the layered and multi-input autonomous biological funneling. **b** Fermentation performance of the CCM-producing strains using lignin-related mixtures with enhancement of downstream key gene *catA*, in which intermediates undetectable during fermentation are not shown. **c** Fermentation performance of the CCM-producing strain using lignin-related mixtures with enhancement of downstream key gene *pcaHG*, in which intermediates undetectable during fermentation are not shown. **d**–**f** Productions alongside strain growth/glucose consumption in 5 L bioreactors demonstrate SASR7’s capability for scaled-up production of PCA (**d**), CCM (**e**), and β-KA (**f**). **g** Preparation workflow for APL. **h**, **i** Fermentation of SASR7 using APL as substrate, PCA production (**h**) and HPLC chromatograms with annotated peaks of cultures before and after fermentation (**i**). FA ferulic acid, *p*-CA *p*-coumaric acid, CaA caffeic acid, PCA protocatechuic acid, 4-HBA *p*-hydroxybenzoate, CCM *cis,cis*-muconic acid, β-KA β-Ketoadipic acid. Values are presented as mean ± s.d. (*n* = 3 independent biological replicates) in (**b**, **c**, **h**). Source data are provided as a Source Data file.

To evaluate the industrial scalability of the layered and multi-input autonomous biological funneling system, we first performed fed-batch fermentation using strain SASR7 in a 5-L bioreactor. The initial total feed contained 49.38 mM lignin-related phenylpropanoids (*p*-CA:FA:CaA = 4:1:0.5) and approximately 14 g L^−1^ glucose. Substrate consumption was monitored to guide intermittent feeding, maintaining a 1:1 molar ratio between phenylpropanoids and glucose. After 42 h of fermentation with six feeding pulses, a total of 388.80 mM (67.20 g L^−1^) phenylpropanoids were consumed, resulting in the production of 346.48 mM (53.40 g L^−1^) PCA at a productivity of 1.27 g L^−1^/h (Fig. 8d). The high titer and productivity achieved demonstrate efficient PCA production from phenylpropanoids and highlight the industrial potential of this system. Given that PCA serves as a central metabolic hub in *C. glutamicum*, its efficient production provides a versatile platform for the downstream synthesis of other value-added chemicals.

We next extended this platform toward the production of CCM, a key precursor for nylon-6,6 and other commercial polymers. CCM was produced from PCA via decarboxylation to catechol (AroY) followed by ring cleavage (CatA) (Fig. 8a). While *C. glutamicum* has been engineered for CCM production from simple aromatics such as catechol^50^, recent efforts using lignin-related phenylpropanoids have faced challenges with low titers and insufficient regulatory control^22^. To redirect flux, we engineered SASR7 by integrating the *aroY* cassette from *Enterobacter cloacae* with the chaperones *ecdB/ecdD* and deleting the competitive *catB* gene, resulting in strain SASR7-C1. Further tuning of *catA* expression with constitutive promoters of three varying strengths yielded the strains SASR7-C1L, C1M, and C1H. Among these compounds, SASR7-C1 nearly completely converted mixed phenylpropanoids to CCM (81.27 mM, 99.98% yield) within 36 h of flask fermentation (Fig. 8b). Control experiments with glucose as the sole carbon source produced negligible amounts of CCM (Supplementary Fig. 11), confirming that the reported 99.98% conversion is attributable to the supplied phenylpropanoid substrates. Scale-up fed-batch fermentation of SASR7-C1 in a 5-L bioreactor yielded 372.25 mM (51.90 g L^−1^) CCM with a productivity of 1.08 g L^−1^ h^−1^ over 48 h with eight feeding cycles, demonstrating a high CCM titer from phenylpropanoids. (Fig. 8e).

We further evaluated β-KA, a promising precursor for advanced nylon materials with superior moisture barrier properties^77^. In *C. glutamicum*, β-KA is synthesized from PCA through four enzymatic steps catalyzed by PcaHG, PcaB, PcaC, and PcaD (Fig. 8a). We evaluated the feasibility of its biosynthesis using layered and multi-input autonomous biological funneling. To enable β-KA accumulation, we deleted *pcaIJ* in SASR7, generating SASR7-K1. This strain converted phenylpropanoids to β-KA with a 79% efficiency but accumulated 4.07 mM PCA, indicating a metabolic bottleneck. To overcome this limitation, we tuned the expression of *pcaHGB* and *pcaC* using constitutive promoters of varying strength, generating the strain SASR7-K10/16/19. The optimized strain SASR7-K19 converted 62.94 mM phenylpropanoids to 62.34 mM β-KA within 24 h at a yield of 99.05% (Fig. 8c). No β-KA was detected when glucose was used as the sole carbon source (Supplementary Fig. 12), confirming the complete dependence on phenylpropanoid substrates. During 5-L fed-batch fermentation, SASR7-K19 produced 239.38 mM (38.33 g L^−1^) β-KA at a productivity of 0.80 g L^−1^ h^−1^ (Fig. 8f).

Finally, to assess the performance on real lignocellulosic feedstocks, we evaluated the system using a lignin-rich liquor derived from the alkaline pretreatment of corn stover [alkaline pretreated liquor (APL)], a lignin-rich stream containing *p*-CA and FA. Without any prior detoxification or washing steps, both substrates were completely consumed within 12 h, accompanied by concomitant PCA production. This process resulted in a PCA titer of 12.38 mM for APL (31.83 mg g^−1^ soluble lignin-related aromatics), corresponding to a molar yield of 99.44% for corn stover-derived phenylpropanoids (Fig. 8g–i). These findings demonstrate the robustness and practical applicability of this platform for the valorization of lignin-related aromatics. Collectively, these results establish a layered and multi-input autonomous biological funneling system as a scalable, versatile, and robust platform for converting lignin-related aromatics into high-value compounds, bridging the gap between laboratory engineering and industrial bioprocessing.

## Discussion

This work establishes a synthetic biological framework for overcoming toxicity and efficiency barriers in the microbial valorization of lignin-related aromatics. Conceptually, our strategy is based on three core principles: (i) strengthening chassis robustness through tolerance engineering, (ii) leveraging native regulatory systems for dynamic pathway control, and (iii) implementing energy-efficient pathways with on-demand gene expression. Together, these elements enable the construction of an autonomous system that coordinates complex metabolic processes with minimal external intervention.

The development of robust microbial platforms for a sustainable lignin-based industry currently represents one of the most promising frontiers in lignin valorization^4^. Previous efforts, particularly in *C. glutamicum*, have shown that reinforcing native biological funneling pathways can significantly improve aromatic conversion. For example, overexpression of the key enzyme CatA enables the production of CCM to reach 85 g L^−1^ when the direct precursor catechol is used^6^. The derepression of transcriptional regulators, such as PhdR and VanR, enhances the flux of the β-oxidation and O-demethylation pathways^22,41^. However, despite these advances, product titers derived from phenylpropanoids remain significantly lower than those achieved using direct feeding of the precursor catechol. This disparity reflects not only the inherent complexity and metabolic constraints of the phenylpropanoid catabolic pathway but also a critical physiological constraint: the toxicity of phenylpropanoids and their metabolic intermediates^41^, which compromises cellular robustness and limits process scalability^50^.

To mitigate the toxicity of aromatic substrates, existing strategies focus primarily on two aspects: bioprocess optimization and enhancing the robustness of the chassis strain. Previous study has implemented precise fed-batch strategies to maintain substrate concentrations below inhibitory thresholds, achieving a β-KA titer of 44.5 g L^−1^ in *P. putida*^21^. While such approaches are effective, they introduce significant operational complexity and cost at the industrial scale. Alternatively, enhancing the endogenous tolerance of microbial hosts offers a more fundamental and scalable solution. In this study, we employed ALE to increase *C. glutamicum* tolerance to phenylpropanoids, enabling robust growth under high substrate concentrations and establishing a solid foundation for high-efficiency conversion. Mechanistically, multiomics and reverse genetics revealed three synergistic tolerance mechanisms: (i) enhanced EPS biosynthesis (Wzz), (ii) efflux pump upregulation (AtsR/MMPL), and (iii) SOS response reprogramming (LexA) to increase genomic stability. These findings not only deepen our mechanistic understanding of how microorganisms adapt to lignin-related inhibitors but also provide a fundamental reference for expanding the aromatic tolerance repertoire in a wide array of industrial microbes. Simply introducing 1–2 mutations can increase the growth capacity of industrial strains under high concentrations of lignin-related inhibitors. This advancement not only promotes the valorization of lignin-related aromatics but also has potential implications for simplifying the costly detoxification steps in traditional lignocellulose biorefining^78^, thereby improving both economic viability and sustainability^9,79,80^.

Beyond toxicity, the structural heterogeneity and compositional variability of lignin-derived feedstocks present additional challenges for efficient bioconversion. Although native bacteria possess biological funneling pathways capable of channeling diverse aromatic compounds into central intermediates^4^, the efficiency of these natural routes remains suboptimal for industrial requirements^81^; these pathways are inherently optimized for survival rather than industrial productivity. Conventional metabolic engineering approaches, which rely on static pathway reinforcement, are poorly suited for handling dynamic fluctuations in substrate composition and metabolic demand^82^. This limitation underscores the need for regulatory systems that can adaptively redistribute metabolic flux in response to real-time substrate inputs. In this context, endogenous aromatic-responsive transcription factors represent a largely underexploited resource for multiplexed dynamic control. In this study, we systematically characterized six native regulators, namely, PhdR, PcaO, BenR, GenR, RolR, and IhtR. In accordance with the design principles of synthetic genetic circuits, we performed a mechanical analysis of the operator site for the pivotal regulator PhdR and validated its ligand affinity. Our results revealed an in vitro-in vivo discrepancy, as the dissociation constant of PhdR measured in vitro ranged from approximately 30–46 μM, whereas the effective response range characterized by in vivo fluorescence assays shifted to the millimolar scale. This phenomenon, which is typically observed in biosensor systems^83^, likely arises from limitations in transmembrane transport that constrain the availability of intracellular ligands^84^ and transcription factor expression levels. By precisely deconstructing these regulatory components, we optimized their outputs to overcome intrinsic limitations, thereby enabling reliable and scalable dynamic control over complex metabolic pathways.

Capitalizing on this foundation, we constructed a layered, multi-input autonomous biological funneling system that couples enzyme expression directly to metabolite availability through a positive closed-loop regulatory architecture. In contrast to constitutive expression systems, this design dynamically aligns pathway flux with substrate input and intermediate accumulation, thereby minimizing metabolic burden and preventing the buildup of toxic intermediates. As a result, the system achieves 99.89% molar conversion of mixed phenylpropanoids to PCA and competitive titers in 5-L bioreactors for PCA (53.40 g L^−1^), CCM (51.90 g L^−1^), and β-KA (38.33 g L^−1^). The simultaneous high-yield production of PCA, CCM, and β-KA underscores the versatility of our layered and multi-input autonomous biological funneling. Our experimental results confirm that the bioconversion of phenylpropanoids is effectively decoupled from glucose-supported cell growth. This finding aligns with recent ^13^C-labeling studies that revealed that growth and phenylpropanoid-to-CCM conversion in engineered *C. glutamicum* occur through a coordinated division of labor^22^. A major challenge in utilizing biomass-derived feedstocks lies in their complex, variable, and heterogeneous composition. The robustness of this design is further highlighted by its ability to maintain high performance across varying substrate compositions, demonstrating strong adaptability to the inherent variability of lignin feedstocks. This adaptability highlights its potential to overcome the compositional variability of biomass feedstocks.

Currently, engineered *C. glutamicum* has demonstrated exceptional performance in the de novo synthesis of aromatic compounds. This microbial platform has achieved titers of 82.7 g L^−1^ PCA^85^ and 88.2 g L^−1^ CCM^86^ from glucose. It produced 85 g L^−1^ CCM from catechol, representing a high CCM titer from lignin-related aromatics^6^. Given its established research foundation and superior metabolic plasticity, the application of *C. glutamicum* to biomanufacturing oriented toward low-cost, renewable substrates, such as lignocellulose-related aromatic mixtures or other inexpensive carbon sources, is highly promising for enhancing the economic viability and environmental sustainability of the entire process^12,87,88^. In this study, we efficiently converted diverse lignin-related phenylpropanoids into products with high conversion rates, yielding notable PCA and CCM titers from phenylpropanoids. However, maintaining high conversion rates proved challenging during scale-up to bioreactor cultivation. This phenomenon aligns with classical scaling up effects in fermentation engineering caused by many factors^89,90^. Despite these challenges, process optimization remains a clear and viable trajectory for further titer enhancement. This is exemplified by pioneering research in which, through the optimization of feeding strategies, the current record for β-KA production from alkaline-pretreated corn stover-derived aromatics was established in *P. putida*^21^. Such advancements provide valuable benchmarks and strategic directions for the subsequent development of our fermentation processes.

The widespread availability of lignin-rich agricultural residues positions them as attractive feedstocks for sustainable biomanufacturing. Although phenylpropanoids constitute only a minor fraction of the lignocellulosic complex, typically 3–4% by weight, their biological significance is strongly amplified by their susceptibility to release via alkaline depolymerization^22,91,92^. Unlike the recalcitrant core lignin polymer, these pendant groups are readily dissociable^20,93^, thereby providing accessible substrate flux for engineered microbial platforms. However, industrial lignin valorization necessitates a heightened level of metabolic plasticity. A strategic imperative for achieving this goal involves further expanding the substrate repertoire of autonomous biological funneling in *C. glutamicum*. This would enable the host to precisely sense and respond to diverse aromatic profiles and fluctuating concentrations. Such a strategy ensures that engineered strains maintain robust performance across varying depolymerization regimes.

## Methods

### Strains and media

This study employed *C. glutamicum* ATCC 13032 as the parental strain. All bacterial strains used are listed in Supplementary Data 2 with their genotypes. All plasmids were constructed in *E. coli Trans5α* (Beijing TransGen Biotech Co., Ltd., Beijing) cultured in Luria-Bertani (LB) medium supplemented with 50 μg mL^−1^ kanamycin at 37 °C. Engineered *C. glutamicum* were all constructed via electroporation, with single colonies cultivated on fresh BHIS agar plates. All liquid culture medium of *C. glutamicum* were defined CGXII (with 10 g L⁻¹ glucose and remove PCA). For *C. glutamicum* strains carrying plasmids, kanamycin was added to the liquid medium at 25 μg mL^−1^. *p*-CA, FA, CaA, VA, 4-HBA, PCA, CCM, β-KA, GA, BA, 13-DB, and 14-DB were purchased from Shanghai Aladdin Biochemical Technology Co., Ltd. (Shanghai, China).

All aromatics were prepared as stock solutions and added to the culture medium at designated concentrations (including during batch feeding). Stock solutions of *p*-CA, FA, and 4-HBA were prepared at 3 M in DMSO, while GA, BA, 13-DB, 14-DB, 12-DB, CaA, and VA stocks were formulated at 2 M in DMSO.

### Plasmid construction

Plasmids were constructed using Gibson assembly and sequence-verified. All engineered plasmids and their information were listed in Supplementary Data 3. Primers were designed using SnapGene 6.0.2, and their sequences were provided in Supplementary Data 4. Sequences for *pobA*^*ppu*^, *pobA*^*paj*^, *vanAB*^*acb*^, *vanAB*^*cef*,^ *vanAB*^*ppu*^, and *GFP* were sourced from the National Center for Biotechnology Information. All sequences were codon-optimized and synthesized by Beijing Qingke Biotechnology Co., Ltd. (Beijing,China), and their amino acid sequences were provided in Supplementary Table 1. Gene *phdA*, *phdE*, *phdB*, *phdC*, *pobA*^*cgl*^, and *vanAB*^*cgl*^ were amplified from the genome of *C. glutamicum* ATCC 13032. Similarly, the transcription factors and promoter fragments for six biosensors were also amplified from the genome of *C. glutamicum* ATCC 13032.

### Fermentation strategy using lignin-related aromatics

Seed cultures were first grown overnight in 5 mL BHIS in test tubes at 30 °C, 220 rpm. These pre-cultures were then inoculated into 300 mL baffled flasks containing 25 mL of defined CGXII (removed PCA and with glucose concentration of 10 g L^−1^) at an initial OD_600_ of 0.15. *p*-CA, FA, and CaA (or their proportionate mixture) were supplemented to specified concentrations at fermentation initiation. All cultivations were maintained at 30 °C, 220 rpm. Metabolite sampling and analysis were conducted at predetermined time intervals throughout the fermentation process.

Fed-batch fermentations for PCA, CCM, and β-KA were conducted in 4 L working volume Biostat B bioreactors. Secondary seed cultures (OD₆₀₀ = 15) were inoculated to initial OD₆₀₀ = 1.5. Fermentation medium was defined CGXII medium contained 14 g L^−1^ glucose and 50 mM phenylpropanoids mixture (4: 1: 0.5 *p*-CA: FA: CaA). Conditions maintained at 30 °C, 30% DO (agitation/aeration cascade), and pH 7.0 (NH₄OH/H₂SO₄ control). Substrate consumption was monitored to guide intermittent feeding, maintaining a 1:1 molar ratio between phenylpropanoids and glucose. Periodic sampling quantified metabolites and substrates.

### Quantification of metabolites

PCA, *p*-CA, FA, CaA, 4-HBA, VA, and CCM were analyzed using a Shimadzu HPLC system (Japan) equipped with an SPD-M20A detector. Separation of PCA, *p*-CA, FA, CaA, 4-HBA, and VA was achieved using a C18 column (150 × 4.6 mm, 3 μm; Shimadzu) maintained at 40 °C. Chromatographic separation employed mobile phase A (0.1% formic acid) and mobile phase B (acetonitrile) at a constant flow rate of 1.0 mL/min, with the following gradient program: 0–15 min (0–50% B), 15–20 min (50% B constant), 20–21 min (50–0% B), and 21–25 min (0% B constant). CCM analysis utilized an Aminex HPX-87H organic acid column (300 × 7.8 mm; Bio-Rad) at 40 °C, with 5 mM sulfuric acid at a constant flow rate of 0.5 mL/min for 45 min. A detection wavelength of 254 nm was applied for all analytes except CCM, which was monitored at 262 nm. β-KA quantification followed established protocols: β-KA was converted to levulinic acid (LA) by adding 10 μL sulfuric acid (5 M) per 1 mL sample, followed by heating at 50 °C for ≥8 h. LA was subsequently analyzed using the Shimadzu HPLC equipped with an RID-10A detector and Aminex HPX-87H organic acid column (300 × 7.8 mm; Bio-Rad) using 5 mM sulfuric acid as mobile phase under constant flow rate of 0.6 mL min^−1^ at 50 °C for 20 min.

### Growth assay and adaptive laboratory evolution of *C. glutamicum*

In growth experiments with *C. glutamicum* ATCC 13032, endpoint OD_600_ measurements were conducted in 300 mL baffled flasks. Strains were inoculated at an initial OD_600_ of 0.15 into 25 mL defined CGXII medium and cultivated at 30 °C with 220 rpm agitation for 36 h, after which final OD_600_ were determined spectrophotometrically. Growth curve analysis was conducted in 24-well plates by inoculating cultures with an initial OD_600_ of 0.15 into 1 mM defined CGXII medium. The plates were incubated at 30 °C, with OD_600_ recorded at 20-min intervals. Continuous orbital shaking was applied for 16 min every 4 min throughout the incubation period.

Laboratory adaptive evolution of strains for *p*-CA, FA and CaA tolerance was performed in triplicate. Each evolution was cultivated in 300 mL baffled flasks containing 25 mL defined CGXII medium supplemented with initial substrate concentrations of 12 mM *p*-CA, 22 mM FA, or 22 mM CaA. Initial inoculation OD_600_ was 0.2 and subculture was performed when OD_600_ exceeded 6.0. Substrate concentrations were increased by 2 mM increments after 3–4 passages under constant substrate concentration, with sequential passaging continued accordingly.

### Whole genome resequencing

Cultures of 9 evolved strains (STELA1-STALE9) were harvested at the exponential growth phase (OD600 ≈ 8.0) in 50 mL defined CGⅩⅡ medium. NGS libraries were prepared from 200 μg Covaris-sheared genomic DNA (300–350 bp fragments) using manufacturer protocols. Fragments underwent end-repair/adapter ligation, bead-based size selection, and 8-cycle PCR amplification with indexed P5/P7 primers for flow-cell binding and multiplexing. Libraries were validated (Agilent 2100 Bioanalyzer) and PE150 sequenced on Illumina Hiseq Xten/Novaseq/MGI2000 platforms. The reads were QC and then assembled using velvet, gapfilled with SSPACE and GapFiller^94^. The Prodigal gene finding software has been used for finding coding genes in genome. Transfer RNAs were predicted by tRNAscan-SE under default parameters, with rRNAs identified using Barrnap and other non-coding RNAs annotated via Rfam. Protein-coding gene functions were assigned through Diamond searches against the NCBI nr database (*E* < 1 × 10^−5^), followed by Gene Ontology (GO) annotation and KEGG pathway mapping. Proteins were phylogenetically classified using Clusters of Orthologous Groups), and functional domains were characterized by Diamond screening against CAZy, SwissProt, Pfam, CARD, VFDB, and DFVF databases applying a stringent *E*-value threshold of <1 × 10^−5^ The genome resequencing was performed by Azenta Life Sciences (Suzhou, China).

### Transcriptome analysis

Biological triplicates of the evolved strain STALE9 and wild-type *C. glutamicum* ATCC 13032 were harvested at the exponential growth phase (OD_600_ ≈ 8.0) in 50 mL defined CGXII medium supplemented with 16 mM *p*-CA. Biological triplicates of the unevolved *C. glutamicum* ATCC 13032 were collected at the same growth phase in 50 mL defined CGXII medium without substrate supplementation. Total RNA was extracted via CTAB with DNA removal, followed by ribosomal RNA depletion (RiboCop Kit). Strand-specific mRNA libraries were prepared using Illumina® Stranded mRNA Prep: fragmentation (~ 300 bp), dUTP-based strand marking, end-repair/A-tailing, and Y-adapter ligation. Libraries were sequenced on Illumina NovaSeq X Plus (PE150). Bioinformatics analysis (Majorbio Cloud) included expression quantification (RSEM v1.3.3), DEG identification with DESeq2 (v1.40.2; |log₂*FC*| ≥0.6 & *p*-FDR < 0.05), and functional enrichment via GOATools (v1.3.6) and KEGG pathways using Fisher’s exact test with FDR correction (*p* < 0.05).

### Real-time qPCR analysis

RT-qPCR was employed to quantify transcriptional levels of *CGL_RS04630* in AtsR mutant strains versus control strains, along with *phdB*, *pobA*, and *vanAB* gene expressions in SASR7. Cells of evolved strain STALE9, *C. glutamicum* ATCC 13032, and four mutant strains (AtsR*-1, AtsR*-2, AtsR*-3, AtsR*-com) were harvested during the early logarithmic growth phase (OD_600_ ≈ 5). For SASR7, samples were collected at 3-h intervals over 0–33 h. Total RNA was isolated using the RNAprep Pure Bacterial RNA Extraction Kit (TIANGEN, China) and reverse-transcribed into cDNA with the ReverTra Ace qPCR RT Kit (TOYOBO, Japan). Quantitative PCR (qPCR) amplification was performed on a StepOnePlus® Real-Time PCR System (Applied Biosystems, USA) using SYBR® Select Master Mix (2×) (Applied Biosystems, America), with triplicate technical replicates per sample. Relative quantification analysis utilized the ${2}^{-\Delta \Delta {{{\rm{CT}}}}}$ method, normalizing against 16S rRNA as an endogenous reference. All RT-PCR procedures were conducted by Qingdao Weilai Biotechnology Co., Ltd. (Qingdao, China).

### Extracellular polysaccharides (EPS) quantification

After 16-h cultivation, cultures of wild-type *C. glutamicum*, the evolved strain STALE9, and constructed mutant strains (Wzz* and GT*) were centrifuged at 10,625 × *g* for 10 min. The supernatant was collected and diluted tenfold for EPS quantification using an Extracellular Polysaccharides ELISA Kit (Baililai Biology, Shanghai). The procedure was performed as follows: 50 μL of standard solution were loaded into standard wells. Samples were dispensed at the well bottom without touching the walls and gently agitated. The plate was sealed and incubated at 37 °C for 30 min. After removing the sealing film, wells were emptied and blotted dry. Each well was filled with wash buffer, incubated for 30 s, and emptied; this wash cycle was repeated five times followed by thorough blotting. 50 μL of enzyme conjugate reagent were added to all wells except blanks. The plate was resealed and incubated at 37 °C for 30 min. Washing was repeated as described above. 50 μL of chromogen substrate A and then 50 μL of substrate B were added to each well. After gentle shaking, the plate was incubated at 37 °C for 10 min in darkness. The reaction was terminated by adding 50 μL of stop solution per well. Absorbance at 450 nm was measured within 15 min after termination, using blank wells for zero calibration.

### Protein expression and purification

The PhdR protein was overexpressed in *E. coli* BL21(DE3) using the pET-28a vector. Seed cultures were inoculated into LB medium supplemented with 100 μg mL^−1^ kanamycin and incubated at 37 °C with 220 rpm. When the OD_600_ reached 0.2, 2 mM IPTG was added to induce expression. The culture conditions were adjusted to 25 °C and 150 rpm for overnight incubation (IhtR protein expression: 16 °C, 150 rpm). Cells were harvested by centrifugation at 4 °C, resuspended in lysis buffer (300 mM NaCl, 50 mM Tris-HCl, DNase I), and lysed using a ultra-high-pressure homogenizer (900 bar, 5 min). The lysate was centrifuged at 4 °C for 10 min. The clarified supernatant was loaded onto a Ni²⁺-charged HisTrap FF column (GE Healthcare, USA) pre-equilibrated with binding buffer. After washing by 50 mM imidazole, bound proteins were eluted using buffer containing 250 mM imidazole. Following elution from Ni-NTA affinity chromatography, PhdR was further purified by anion-exchange chromatography using a Mono Q 10/100 column (GE Healthcare), and subsequently polished by size-exclusion chromatography on a Superdex™ 200 Increase 10/300 GL column (Cytiva) equilibrated with buffer containing 50 mM NaCl, 20 mM Tris-HCl, and 10% glycerol. PhdR purity was confirmed by SDS–PAGE (Supplementary Fig. 13a). Purified protein was aliquoted, flash-frozen in liquid nitrogen, and stored at −80 °C until use.

### Isothermal titration calorimetry

ITC assays were performed using a MicroCal PEAQ-ITC system (Malvern Panalytical Ltd, UK). Ligands were dissolved in protein equilibration buffer, with the pH adjusted to 7.0. After iterative optimization of titration conditions, the final concentrations of the ligands were determined as follows: 0.125 g L^−1^ for *p*-CA, 0.115 g L^−1^ for FA, and 0.23 g L^−1^ for CaA. The concentration of PhdR protein was quantified using a BCA Protein Assay Kit (Beyotime Biotechnology, China; see Supplementary Fig. 13b for the standard curve) and subsequently diluted to the desired concentrations: 1.05 g L^−1^ for titration with *p*-CA, 1.72 g L^−1^ for CaA, and 0.77 g L^−1^ for FA. For each experiment, the sample cell was loaded with the protein solution, while the reference cell was filled with dd-H₂O. The corresponding ligand solution was loaded into the injection syringe. A titration protocol consisting of 20 injections was applied: an initial injection of 0.4 μL of ligand solution was followed by 19 subsequent injections of 2 μL each. Control experiments were conducted by titrating each ligand solution into the protein equilibration buffer alone. Data were analyzed using the integrated MicroCal PEAQ-ITC analysis software, with the binding isotherms fitted using the One Set of Sites model. Raw data and fitting parameters for ITC were provided in Supplementary Data 5–7.

### DNase I footprinting assay

DNase I footprinting was performed by Sangon Biotech (Shanghai, China). FAM-labeled probes were generated by amplifying target promoter regions from *C. glutamicum* ATCC 13032 genomic DNA with 5′-FAM-modified primers. Probes were purified using Wizard® SV Gel and PCR Clean-Up System (Promega, USA) and quantified by NanoDrop 2000C spectrophotometry (Thermo Fisher, USA). Reaction mixtures containing 337 ng FAM-pPhdR probe or 423 ng FAM-pIHTR probe were incubated with purified Dark protein (0–8 μg) in binding buffer for 30 min at 25 °C in 40 μL total volume. Enzymatic digestion was initiated by adding 10 μL solution containing 0.015 units DNase I (Promega) and 100 nmol CaCl₂, followed by 37 °C incubation for 1 min. Reactions were terminated with 140 μL stop solution (200 mM sodium acetate, 30 mM EDTA, 0.15% SDS). Samples were extracted with phenol/chloroform, ethanol-precipitated, and pellets dissolved in 30 μL MiniQ water. DNA ladders were prepared using the fmol DNA Cycle Sequencing System (Promega). Digested DNA fragments and sequencing products were mixed with HiDi formamide and GeneScan-LIZ600 size standard (Applied Biosystems), and analyzed using a 3130xl DNA Analyzer (Applied Biosystems).

### Fluorescence assay and flow cytometry assay

*C. glutamicum* strains harboring the fluorescent reporter plasmid were inoculated into 24-well plates with an initial OD_600_ of 0.1. Lignin-related aromatics stock solutions were supplemented to achieve target final concentrations during inoculation. After 36 h cultivation, cells were harvested by centrifugation, washed twice with 1× PBS buffer, and resuspended in equal volume of 1× PBS buffer. Green fluorescence intensity (excitation: 485 nm; emission: 528 nm) and OD_600_ were measured using a SYNERGY HTX multi-mode reader (BioTek, USA). For dose-response fluorescence characterization experiments, identical culture conditions, pretreatment protocols, and detection methods were employed, except cells were harvested at 12 h.

In flow cytometry assays, harvested cells were washed twice with 1× PBS buffer, resuspended in 100 volum of 1× PBS buffer, filtered through a 100-mesh nylon sieve, and analyzed using a BD FACSCalibur flow cytometer equipped with a 488 nm excitation laser and FL1 detector (530/30 nm bandpass filter), 1 × 10^5^ individual cells were analyzed for each presented fluorescence distribution plot. FlowJo v10 was used to analyze data from Flow cytometry assay.

### Spot growth assay

Colonies were picked from fresh BHIS agar plates and inoculated into liquid BHIS medium for overnight incubation. The seed culture was then transferred to fresh BHIS medium with an initial OD_600_ of 0.1. Upon reaching an OD_600_ of 3, cultures were serially diluted to OD_600_ values of 1, 0.1, 0.01, 0.001, 0.0001, 0.00001, and 0.000001. Subsequently, 1 μL aliquots of each dilution were spot-inoculated onto BHIS agar plates supplemented with 16 mM *p*-CA, followed by incubation at 30 °C for 2–3 days to monitor bacterial growth.

### Preparation of alkaline pretreated liquor (APL)

Corn stover was pulverized and sieved to collect particles sized 20–80 mesh. The biomass fraction was subjected to alkaline pretreatment employing 1 wt% NaOH solution at a solid-to-liquid ratio of 10% w/v, pretreatment was conducted at 130 °C for 30 min. The resultant mixture was filtered through a 200-mesh filter bag to obtain APL. APL was subsequently centrifugated to remove insoluble particulates and concentrated by drying at 50 °C. The resulting solid was ground into powder. For fermentation media preparation, the required mass was calculated based on target monomer concentrations, weighed, and dissolved in defined CGXII medium. The medium was sterilized by autoclaving at 115 °C for 15 min prior to use as APL fermentation broth.

### Statistical analysis

All data were presented as mean values ± SD from three independent biological replicates (*n* = 3).

### Reporting summary

Further information on research design is available in the Nature Portfolio Reporting Summary linked to this article.

## Supplementary information

Supplementary Information

Description of Additional Supplementary Files

Supplementary Data 1

Supplementary Data 2

Supplementary Data 3

Supplementary Data 4

Supplementary Data 5

Supplementary Data 6

Supplementary Data 7

Reporting Summary

Peer Review file

## Source data

Source Data

## Data Availability

The whole genome resequencing and transcriptome sequencing data generated in this study have been deposited in the NCBI Sequence Read Archive under accession PRJNA1374442. Source data are provided with this paper.
