# Supplementary material for: Engineering a robust and autonomous biological funneling for efficient valorization of lignin-related aromatics
Source: Nat Commun. 2026 Jun 17;17:7659. doi: 10.1038/s41467-026-74424-y (PMC13434420; doi:10.1038/s41467-026-74424-y)
Supplement: Supplementary file 1 [file 41467_2026_74424_MOESM10_ESM.pdf]

nature portfolio

Corresponding author(s): Qian Wang, Qingsheng Qi

Last updated by author(s): May 29, 2026

Reporting Summary

Nature Portfolio wishes to improve the reproducibility of the work that we publish. This form provides structure for consistency and transparency in reporting. For further information on Nature Portfolio policies, see our Editorial Policies and the Editorial Policy Checklist.

Statistics

For all statistical analyses, confirm that the following items are present in the figure legend, table legend, main text, or Methods section.

n/a	Confirmed	
☐	☒	The exact sample size (*n*) for each experimental group/condition, given as a discrete number and unit of measurement
☐	☒	A statement on whether measurements were taken from distinct samples or whether the same sample was measured repeatedly
☐	☒	The statistical test(s) used AND whether they are one- or two-sided<br>*Only common tests should be described solely by name; describe more complex techniques in the Methods section.*
☒	☐	A description of all covariates tested
☒	☐	A description of any assumptions or corrections, such as tests of normality and adjustment for multiple comparisons
☐	☒	A full description of the statistical parameters including central tendency (e.g. means) or other basic estimates (e.g. regression coefficient) AND variation (e.g. standard deviation) or associated estimates of uncertainty (e.g. confidence intervals)
☒	☐	For null hypothesis testing, the test statistic (e.g. *F*, *t*, *r*) with confidence intervals, effect sizes, degrees of freedom and *P* value noted<br>*Give P values as exact values whenever suitable.*
☒	☐	For Bayesian analysis, information on the choice of priors and Markov chain Monte Carlo settings
☒	☐	For hierarchical and complex designs, identification of the appropriate level for tests and full reporting of outcomes
☒	☐	Estimates of effect sizes (e.g. Cohen's *d*, Pearson's *r*), indicating how they were calculated

Our web collection on statistics for biologists contains articles on many of the points above.

Software and code

Policy information about availability of computer code

Data collection	High Performance Liquid Chromatography (Shimadzu), Spectrometer (Shimadzu), SYNERGY HTX multi-mode reader (Biotek), StepOnePlus® Real-Time PCR System (Applied Biosystems), MicroCal PEAQ-ITC system (Malvern Panalytical), FACSCalibur flow cytometer (BD Biosciences).
Data analysis	The following commercial software was used for data analysis: OriginPro 8.5, GraphPad Prism 8.3.0, Microsoft Excel 2021, LabSolutions LCWorkstation Ver.5, and FlowJo v10. Binding affinity calculations were performed using the MicroCal PEAQ-ITC Analysis Software v1.41.

For manuscripts utilizing custom algorithms or software that are central to the research but not yet described in published literature, software must be made available to editors and reviewers. We strongly encourage code deposition in a community repository (e.g. GitHub). See the Nature Portfolio guidelines for submitting code & software for further information.

Data

Policy information about availability of data

All manuscripts must include a data availability statement. This statement should provide the following information, where applicable:

- Accession codes, unique identifiers, or web links for publicly available datasets
- A description of any restrictions on data availability
- For clinical datasets or third party data, please ensure that the statement adheres to our policy

The main data supporting the findings of this study are available within the article and its supplementary information files. The whole genome resequencing and

transcriptome sequencing data generated in this study have been deposited in the NCBI Sequence Read Archive under accession code PRJNA1374442 [https://www.ncbi.nlm.nih.gov/bioproject/PRJNA1374442]. The other data generated in this study are provided in Source Data file. Source data are provided with this paper.

Research involving human participants, their data, or biological material

Policy information about studies with human participants or human data. See also policy information about sex, gender (identity/presentation), and sexual orientation and race, ethnicity and racism.

Reporting on sex and gender	Not applicable
Reporting on race, ethnicity, or other socially relevant groupings	Not applicable
Population characteristics	Not applicable
Recruitment	Not applicable
Ethics oversight	Not applicable

Note that full information on the approval of the study protocol must also be provided in the manuscript.

Field-specific reporting

Please select the one below that is the best fit for your research. If you are not sure, read the appropriate sections before making your selection.

☒ Life sciences ☐ Behavioural & social sciences ☐ Ecological, evolutionary & environmental sciences

For a reference copy of the document with all sections, see nature.com/documents/nr-reporting-summary-flat.pdf

Life sciences study design

All studies must disclose on these points even when the disclosure is negative.

Sample size	Generally, three biological replicates were used for data analysis, consistent with standard practice in the field and sufficient to ensure stable and reproducible results.
Data exclusions	No data was excluded from the analysis.
Replication	Fermentation strategies using lignin-related aromatics in shake flasks were performed with biological triplicates. Laboratory adaptive evolution of strains for tolerance to p-coumaric acid (p-CA), ferulic acid (FA), and caffeic acid (CaA) was performed with biological triplicates. Endpoint OD_{600} measurements of C. glutamicum after adaptive laboratory evolution were carried out with biological triplicates. Growth curve analysis in 24-well plates was performed with biological duplicates. Whole-genome resequencing, RNA sequencing, RT-qPCR, extracellular polysaccharide quantification, and green fluorescence intensity assays were all performed with biological triplicates. All replication attempts were successful.
Randomization	Most experiments in this study did not involve random allocation, as all samples were processed under identical conditions. In the adaptive laboratory evolution experiment, evolved strains were assigned to the treatment group, the wild-type Corynebacterium glutamicum strain served as the control group, and single colonies were randomly selected for whole-genome sequencing.
Blinding	Not relevant because no group allocation was involved in this study.

Reporting for specific materials, systems and methods

We require information from authors about some types of materials, experimental systems and methods used in many studies. Here, indicate whether each material, system or method listed is relevant to your study. If you are not sure if a list item applies to your research, read the appropriate section before selecting a response.

Materials & experimental systems

n/a	Involved in the study
☒	☐ Antibodies
☒	☐ Eukaryotic cell lines
☒	☐ Palaeontology and archaeology
☒	☐ Animals and other organisms
☒	☐ Clinical data
☒	☐ Dual use research of concern
☒	☐ Plants

Methods

n/a	Involved in the study
☒	☐ ChIP-seq
☐	☒ Flow cytometry
☒	☐ MRI-based neuroimaging

Plants

Seed stocks

Report on the source of all seed stocks or other plant material used. If applicable, state the seed stock centre and catalogue number. If plant specimens were collected from the field, describe the collection location, date and sampling procedures.

Novel plant genotypes

Describe the methods by which all novel plant genotypes were produced. This includes those generated by transgenic approaches, gene editing, chemical/radiation-based mutagenesis and hybridization. For transgenic lines, describe the transformation method, the number of independent lines analyzed and the generation upon which experiments were performed. For gene-edited lines, describe the editor used, the endogenous sequence targeted for editing, the targeting guide RNA sequence (if applicable) and how the editor was applied.

Authentication

Describe any authentication procedures for each seed stock used or novel genotype generated. Describe any experiments used to assess the effect of a mutation and, where applicable, how potential secondary effects (e.g. second site T-DNA insertions, mosiacism, off-target gene editing) were examined.

Flow Cytometry

Plots

Confirm that:

☒ The axis labels state the marker and fluorochrome used (e.g. CD4-FITC).

☒ The axis scales are clearly visible. Include numbers along axes only for bottom left plot of group (a 'group' is an analysis of identical markers).

☐ All plots are contour plots with outliers or pseudocolor plots.

☒ A numerical value for number of cells or percentage (with statistics) is provided.

Methodology

Sample preparation

C. glutamicum strains harboring the fluorescent reporter plasmid were inoculated into 24-well plates, and cells were harvested by centrifugation, washed twice with 1× PBS buffer, and resuspended in equal volume of 1× PBS buffer.

Instrument

FACSCalibur flow cytometer (BD Biosciences)

Software

FlowJo v10 was used to analyze data from Flow cytometry assay

Cell population abundance

The sorting function of the flow cytometer was not used.

Gating strategy

No gating strategy was used as the experiments were only conducted for analysis of fluorescence intensity.

☐ Tick this box to confirm that a figure exemplifying the gating strategy is provided in the Supplementary Information.
