# Supplementary material for: Engineering a robust and autonomous biological funneling for efficient valorization of lignin-related aromatics
Source: Nat Commun. 2026 Jun 17;17:7659. doi: 10.1038/s41467-026-74424-y (PMC13434420; doi:10.1038/s41467-026-74424-y)
Supplement: Supplementary file 2 [file 41467_2026_74424_MOESM11_ESM.pdf]

nature portfolio

Peer Review File

Engineering a robust and autonomous biological funneling for efficient valorization of lignin-related aromatics

Corresponding Author: Professor Qian Wang

This file contains all reviewer reports in order by version, followed by all author rebuttals in order by version.

Attachments originally included by the reviewers as part of their assessment can be found at the end of this file.

Version 0:

Reviewer comments:

Reviewer #1

(Remarks to the Author)
In this work, the authors engineer Corynebacterium glutamicum to convert lignin-derived phenylpropanoids into protocatechuic acid, cis,cis-muconic acid, and β-ketoadipic acid. They employ adaptive laboratory evolution to improve aromatic efflux and stress tolerance, and further repurpose native aromatic-responsive regulators as biosensors to enable dynamic regulation of pathway flux. The reported conversions and titers are impressive, and the study is technically thorough. However, while the implementation is strong, the conceptual framework itself is not new. The use of substrate- and intermediate-responsive regulatory elements to achieve layered, multi-input, autonomous dynamic control of metabolic pathways has been previously established in the metabolic engineering literature. Introducing a new term ("MASTER") for an existing class of regulatory strategies risks normalizing a practice in which established concepts are repeatedly rebranded, rather than built upon. This sets an undesirable precedent for the field and obscures continuity with prior work. The authors are therefore encouraged to frame their approach explicitly within the existing literature on layered and multi-input dynamic metabolic control, rather than coining new terminology, and to cite relevant prior work accordingly. Please refer to – "Layered and multi-input autonomous dynamic control strategies for metabolic engineering, Current Opinion in Biotechnology".
The manuscript would benefit from improved clarity and precision throughout. Specific examples from the Introduction include:
• Lines 46–47: "However, Lignin industrialization for …" – the phrase "lignin industrialization" is unclear and should be revised for clarity.
• Line 63: Consider clarifying "lignin biorefining" or providing context, as the current wording may be confusing.
• Line 84: There appears to be a missing period before "despite."
• Line 83: "Thus, C. glutamicum is among the most promising organisms" – it is unclear what it is promising for; please specify (e.g., for lignin valorization, aromatic compound conversion, etc.).
• Lines 86–88: Two consecutive sentences start with "Although," which disrupts flow. Consider varying the sentence structure or combining clauses to improve readability.
Overall, the manuscript would benefit from careful revision to strengthen language, improve clarity, and enhance readability. The reviewer notes that while the manuscript presents a substantial amount of data in figures, the figures should not be so small that text and labels are difficult to read without zooming in. For example, Fig. 3.
Although performing a T-test with only three replicates has become common in the field, this is statistically unnecessary. T-tests are meaningful primarily when claiming true "significance," which requires sufficient replicates. Using a T-test in this context serves little purpose. The reviewer therefore recommends reporting results as fold-changes, relative comparisons, or qualitative assessments of higher versus lower values, rather than applying T-tests merely for formality. Avoiding such unnecessary overuse will help prevent setting a misleading precedent for future researchers in the field.

Reviewer #2

(Remarks to the Author)
Xu et al report various approaches, including classical pathway over-expression and adaptive laboratory evolution to improve the biotransformation of phenylpropenoic acids into diacid products. The approach employed and manuscript is

extensive, if not entirely revolutionary in terms of methods employed or production titres achieved for the same products in related hosts. However, the master rectory system is elegant and seeks the better match regulatory control with a higher production titles required in biotechnology. A major concern I have is readability, framing of the research the transparency within which this study is place within the wider body of literature.

Major:
1. The manuscript throughout claims the research study is a lignin valorisation strategy whereas in reality this should be referred to as a lignocellulosic valorisation strategy. Indeed, the phenylpropenoic acids utilised are only yielded in small quantities from the hemicellulose fraction of lignocellulosic biomass, where is in the actual lignin fractions the G, H and C type monomers are cross-linked through carbon-carbon and carbon ether bonds. Neither thermochemical or enzymatic deconstruction yields any significant proportion of these phenyl propanoic acids. This should be revision throughout
2. The introduction seems overly focused on championing the chosen host organism C. glutamicum as a host for 'lignin valorisation'. As written it seems to suggest that this is the first time that C. glutamicum has been used for aromatic catabolism into products which is untrue as there is extensive literature from the Wittman laboratory on the same topic. Indeed, higher muconic acid titres have previously reported from the same, in excess of 80 g/L from lignin-derived aromatics (10.1186/s12934-018-0963-2)
3. Further the tone of the introduction is written in quite a colloquial / unprofessional manner flagging the C. glutamicum performs "the star strain Pseudomonas putida" Further no reference is given to all that several other bacterial strains have been reported to display aromatic/lignin monomer catabolic capabilities such as Comanonas sp. and R josti. I suggest that the authors review the extensive literature on these topics specifically from the Bugg, Eltis, Masai, Aristilide labs
4. The manuscript title and throughout reports the development of microbial hosts, whilst such hosts could utility within biorefinery, the host alone is neither the biorefinery or platform in themselves. So, the authors to tone down the claims that this is now lignin biorefinery platform
5. A major challenge with utilising biomass derived feedstocks is the complex variable and a heterogenous composition - therefore did the authors explore the performance of the master system under different compositions for example decreasing ferulic acid and increasing coumaric acid? This would strength the claims around industrial utility
6. It would also be useful to benchmark the performance of the master control system against an unregulated or natively regulated system for example does this complex genetic control circuitry offer advantages above constitutive expression of all genes at once?
7. Discussion I found this part of the manuscript to be very inward looking and was essentially just a summary of the results with only a small scattering of references. This section could do with an extensive rewrite placing the work more fully in the context of the surrounding literature discussing openly and frankly what other titres have achieved for muconate and related products in this host and other hosts, and the need to push production titles as well as expanding microbial metabolism to utilise lower cost feedstocks such as aromatics. This section could also do with a frank rationalisation of the concentration of the phenylpropenoic acids utilised as feedstocks here generally only present in 3 to 4% maximum of lignocellulosic biomass (in their ester linked form) whereas lignin deconstruction releases a different class of aromatic momomers.
8. Lots of strains used with lots of completed nomenclature, provide a table with clear description with names, nodes and components changed description. Similarity figure legends do not provide all the key information including conditions used

Minor
L19 – meaning unclear. What are downstream metabolic capabilities presumably that refers to central metabolism whereas the focus here is on upper catabolic pathways for this assimailt of aromatics into central metabolism.
L24 – here ALE was employed to generate…
L30 – claims that these are their highest reported titles for muconic acid from lignin derived aromatics however Becker et al have reported muconic acid titres in excess of 80 g/L from lignin-derived aromatics (10.1186/s12934-018-0963-2
L46 "lignin" not "Lignin"
L54 "have evolved"
L63 "pivotal breakthrough to the commercialization of lignin" this claim is unsupported. Boregard is the only established lignin-based biorefinery, so how this native catabolic capability a breakthrough? In addition, phenyl propanoic acids are only yielded in small quantities from the hemicellulose fraction of lignocellulosic biomass, where is in the actual leg infractions G, H and C type monomers they are cross-linked through carbon-carbon and carbon ether bonds. Neither thermochemical or enzymatic deconstruction of leading yield and a significant fraction of these phenyl propanoic acids.
L74 "concentrated on merely a singularly dominant bacterial" variation literature reports on other hosts including C. glutamicum and R. josti
L84 "despite" not "Despite"
L143 "Layer 2 acts" not "Layer 2 act"
L146 "to" not "To"
L148 "homologous" not "endogenous" to distinguish between plasmid over expression and native chromosomal copies
L152 "Layer 1" this in canonical referred to as the upper aromatic degradation pathway, terminological consistency strongly advised
L154 "The results showed that" where are these results?
L161 "demonstrating that enhancing phd expression improves Layer 1 efficiency at 161 the cost of aggravating intermediate accumulation." I do not believe this statement to be correct, the PCA titre has not decreased, so nothing has been 'aggravated' – this appears that just the 4-HBA (and VA) to PCA node is the bottleneck
L174 Refers toxic of the aromatics, the SI fig does not clearly show this ie final OD/growth curves +/- the ligand for the w-t and engineered strains – should this refer to Fig3A instead?
Fig 2A – The product of the PhdABC pathway is the aromatic aldehyde, not the acid shown, is Vanillin from ferulic acid is the product not vanillic acid

Fig 2G “KA” should this be “CaA”
L191 “ Spca1 fermentation” what fermentation mode, presumably bolus addition of the aromatics based on the panels b-d
L214 Although post-ALE genome sequencing was performed, and the commonly occurring mutations were shown, Analysis was carried out/communicated as to what these exact commutations were. This should be included in the revised manuscript, and combined with subsequent transcriptomics analysis performed for STALE9 strain
L217 “STALE1-STALE9” define
L218 “achieved” not “achieving”
L239 “We propose that ALE has endowed STALE9 with toxicity resistance, 239 enabling its normal physiological functions recovery under stress.” What evidence is there for toxicity resistance? As earlier comments, it would seem more logical to first analysis the genomic mutation in STALE9.
L320 “derepresses” not “derepress”
Fig 5g ITC y-axis data label and units are incorrect ‘ΔHG’ does not represent a thermodynamic metric, ΔH? Units are measured in ‘kcal/mol’ not ‘Kcal/mol’
The corresponding ligand affinities do not appear to be consistent with the literature being in the 1-10uM range (https://doi.org/10.1093/nar/gkx1055), and indeed the Kd data do not look to be internally consistent, pCA binding curve is looks to be significantly steeper than FA, but the reported Kds are similar. In addition, the CaA does not look to be correctly fitted, the data and fitting parameters were not provided . Finally, the reported molar ratio (1:20 to 1:40) do not appear to be consistent with the known binding stoichiometry, 1:1 or 2:1 of aTFs. As such it would appear that either the protein concentration has been under-estimated or the ligand over-estimated, the protein production reported in the Methods section reports 0.375g/L (~10uM) protein concentration was used but doesn’t include a dialysis step post imidazole elution following His-tag purification, so is likely that a much protein lower concentration is present.
have some serious concerns as to how this ITC was performed and analysed
Fig 6 why in vitro Kd and operating range are so different, mM very higher for regulatory proteins
F9g c-d and lgends not clear what barcharts represent biosensor dynamic range +/- ligand?
L433 define”QF”
L531 “ No previous studies have reported CCM production from 531 lignin-derived phenylpropanoids in C. glutamicum” recently reported here https://doi.org/10.1016/j.ymben.2025.08.004”
L547 “yielding nylon-6,6 analogs with 546 superior moisture barrier properties” add reference
Fig 8c chemical structure contain errors “OH-aromatic” shown rather than HO-aromatic for PCA and bKA
Fig 8e did the authors observe any difference in cell growth under these different strains for example vanAB-mediated demethylation is known to be burdensome upon cell growth, do any differences in cell growth explain the different performance traits observed.
Discussion I found this part of the manuscript to be very inward looking and was essentially just a summary of the results with only a small scattering of references. This section could do with an extensive rewrite placing the work more fully in the context of the surrounding literature discussing openly and frankly what other titles people have achieved for muconate and related products in this host and other hosts, and the need to push production titles as well as expanding microbial metabolism to utilise lower cost feedstocks such as aromatics. This section could also do with a frank rationalisation of the concentration of the phenylpropenoic acids utilised as feedstocks here generally only present in 3 to 4% maximum of lignocellulosic biomass (in their ester linked form) whereas lignin deconstruction releases a different class of aromatic momomers
Supplementary Figure 1 – no concentrations provided for the added aromatics

Reviewer #3

(Remarks to the Author)
Please see the attached document for the detailed review

Reviewer #4

(Remarks to the Author)
I co-reviewed this manuscript with one of the reviewers who provided the listed reports. This is part of the Nature Communications initiative to facilitate training in peer review and to provide appropriate recognition for Early Career Researchers who co-review manuscripts.

Version 1:

Reviewer comments:

Reviewer #1

(Remarks to the Author)
All comments have been addressed satisfactorily, and no further concerns remain.

Reviewer #2

(Remarks to the Author)
Xu el al revision
The authors have made extensive revision to the originally submitted manuscript and significantly tone, rhetoric and

overhyped terminology. They have additionally performed further analysis to i) validate the strain under heterogeneous aromatic compositions, ii) benchmark against a non-inducible strain, and iii) provide ITC data consistent with the literature.

Minor:
Figure one, lower right panel, still includes reference “Master system” terminology which has now been removed from elsewhere in the manuscript.

Reviewer #3

(Remarks to the Author)
The authors have undertaken a thorough revision of the original manuscript submission, including a completely revised Discussion section, as well as many major and minor changes in other sections, in response to the comments and suggestions by different reviewers.

They have also given very detailed responses to the reviewer comments and queries and listed the different changes made in the manuscript.

At this point, I am quite satisfied with their responses as well as the changes made in the revised manuscript. The revised manuscript has vastly improved in quality and clarity and I recommend it for publication without further changes.

I have not checked the manuscript for formatting consistency and similar issues and hope that the editorial staff will take care of these.

Open Access This Peer Review File is licensed under a Creative Commons Attribution 4.0 International License, which permits use, sharing, adaptation, distribution and reproduction in any medium or format, as long as you give appropriate credit to the original author(s) and the source, provide a link to the Creative Commons license, and indicate if changes were made.
In cases where reviewers are anonymous, credit should be given to 'Anonymous Referee' and the source.
The images or other third party material in this Peer Review File are included in the article’s Creative Commons license, unless indicated otherwise in a credit line to the material. If material is not included in the article’s Creative Commons license and your intended use is not permitted by statutory regulation or exceeds the permitted use, you will need to obtain permission directly from the copyright holder.
To view a copy of this license, visit https://creativecommons.org/licenses/by/4.0/

REVIEWER COMMENTS

Reviewer #1: 3

Reviewer #2: 7

Reviewer #3: 31

Reviewer #4: 46

References 47

Reviewer #1:

In this work, the authors engineer *Corynebacterium glutamicum* to convert lignin-derived phenylpropanoids into protocatechuic acid, *cis,cis*-muconic acid, and *β*-ketoadipic acid. They employ adaptive laboratory evolution to improve aromatic efflux and stress tolerance, and further repurpose native aromatic-responsive regulators as biosensors to enable dynamic regulation of pathway flux. The reported conversions and titers are impressive, and the study is technically thorough. However, while the implementation is strong, the conceptual framework itself is not new. The use of substrate- and intermediate-responsive regulatory elements to achieve layered, multi-input, autonomous dynamic control of metabolic pathways has been previously established in the metabolic engineering literature. Introducing a new term ("MASTER") for an existing class of regulatory strategies risks normalizing a practice in which established concepts are repeatedly rebranded, rather than built upon. This sets an undesirable precedent for the field and obscures continuity with prior work. The authors are therefore encouraged to frame their approach explicitly within the existing literature on layered and multi-input dynamic metabolic control, rather than coining new terminology, and to cite relevant prior work accordingly. Please refer to – "Layered and multi-input autonomous dynamic control strategies for metabolic engineering, Current Opinion in Biotechnology".

Response:

- ✓ Thank you for this insightful and constructive comment, and for your recognition of our work. We also appreciate your observation regarding the introduction of new terminology. We apologize for not adequately considering continuity with established concepts in the field. We acknowledge that our strategy should be more clearly framed within the existing literature on layered and multi-input dynamic metabolic control, rather than introducing new terminology, and that the relevant prior studies should be properly cited.
- ✓ In response, we have revised the description of the strategy throughout the manuscript and have added the suggested reference. The corresponding revisions are exemplified below:

  "a Multi-node Artificial Self-regulation Engineered Biorefinery (MASTER) system" has been revised as: "a layered, multi-input autonomous dynamic control system" (Line 103).

  "the MASTER (Multi-node Artificial Self-regulation Engineered Biorefinery) system" has been revised as: "a layered, multi-input autonomous dynamic control

system” (Line 349-350).

“MASTER” has been revised as: “layered and multi-input autonomous dynamic control system” (Line 402).

✓ We are committed to implementing these revisions carefully and believe they will greatly improve the manuscript. We are grateful for the time and expertise you have dedicated to its evaluation. We hope these revisions adequately address your concerns, and we provide detailed, point-by-point responses to the specific comments below.

The manuscript would benefit from improved clarity and precision throughout. Specific examples from the Introduction include:

• Lines 46–47: “However, Lignin industrialization for …” – the phrase “lignin industrialization” is unclear and should be revised for clarity.

Response:

✓ Thank you for pointing out the lack of clarity in this expression. We apologize for the use of imprecise language in the manuscript. We agree that the phrase “lignin industrialization” is ambiguous and may cause confusion. Accordingly, we have replaced “lignin industrialization” with “Efficient valorization of lignin” (Lines 46–47).

✓ In addition, we carefully reviewed the entire manuscript to ensure precision and have revised instances of imprecise or unclear descriptions. We sincerely appreciate the time and effort you have devoted to improving the quality of our manuscript.

• Line 63: Consider clarifying “lignin biorefining” or providing context, as the current wording may be confusing.

Response:

✓ Thank you for the insightful comment, and we apologize for the lack of clarity in our original manuscript. We agree that directly linking biological funneling pathways to “pivotal breakthrough to the commercialization of lignin biorefining” at this stage is premature. Accordingly, we have rewritten the section to focus on the fundamental features and significance of the biological funneling pathway, providing a smoother transition to the following content. The revised text is provided below:

✓ “This funneling strategy provides an inherent solution to lignin heterogeneity by

enabling the convergence of multiple substrates into unified metabolic routes. However, the efficient operation of such pathways requires precise coordination of metabolic flux across multiple enzymatic steps, particularly when processing complex mixtures of phenylpropanoids derived from lignocellulose biomass." (Line 62-67).

• Line 84: There appears to be a missing period before "despite."

Response:

✓ Thank you for pointing out this typographical error. We have corrected the punctuation and reviewed the entire manuscript accordingly. Thank you again for your careful suggestion.

• Line 83: "Thus, *C. glutamicum* is among the most promising organisms" – it is unclear what it is promising for; please specify (e.g., for lignin valorization, aromatic compound conversion, etc.).

Response:

✓ Thank you for this helpful comment, and we apologize for the confusion caused by imprecise language. We have revised the sentence to improve clarity, as follows: "*Corynebacterium glutamicum* has emerged as a promising chassis for lignin valorization" (Lines 82).

• Lines 86–88: Two consecutive sentences start with "Although," which disrupts flow. Consider varying the sentence structure or combining clauses to improve readability.

Response:

✓ Thank you for this thoughtful suggestion. We agree that the repeated use of "Although" at the beginning of consecutive sentences affects the flow. We have revised the sentence structure to improve clarity and readability. Specifically, the consecutive sentences have been rephrased, as follows:

"It possesses inherent biological funneling pathways ... Nevertheless, its potential for coordinated conversion of diverse lignin-derived aromatics remains underexploited. ... Moreover, although several lignin derived aromatics-responsive allosteric transcription factors (aTFs) have been identified, their regulatory mechanisms remain poorly understood." (Lines 84–92).

Overall, the manuscript would benefit from careful revision to strengthen language,

improve clarity, and enhance readability.

Response:

- ✓ Thank you for this constructive comment. We have carefully revised the whole manuscript throughout to improve overall language quality, enhance clarity, and ensure better readability.

The reviewer notes that while the manuscript presents a substantial amount of data in figures, the figures should not be so small that text and labels are difficult to read without zooming in. For example, Fig. 3.

Response:

- ✓ Thank you for this helpful comment. We apologize for the insufficient layout and unclear labeling in the original figure. We have revised Fig. 3 by increasing its size and improving the clarity of the text and labels to ensure readability without the need for excessive zooming.
- ✓ In addition, we have also carefully reviewed the other figures and made similar adjustments where necessary to enhance overall readability.

Although performing a T-test with only three replicates has become common in the field, this is statistically unnecessary. T-tests are meaningful primarily when claiming true "significance," which requires sufficient replicates. Using a T-test in this context serves little purpose. The reviewer therefore recommends reporting results as fold-changes, relative comparisons, or qualitative assessments of higher versus lower values, rather than applying T-tests merely for formality. Avoiding such unnecessary overuse will help prevent setting a misleading precedent for future researchers in the field.

Response:

- ✓ Thank you for this insightful and constructive comment. To clarify and avoid any unnecessary use of statistical significance testing, we have revised the **Materials and Methods** section to remove the reference to T-tests and ensure that data presentation focuses on fold-changes and relative comparisons.

Reviewer #2:

Xu et al report various approaches, including classical pathway over-expression and adaptive laboratory evolution to improve the biotransformation of phenylpropenoic acids into diacid products. The approach employed and manuscript is extensive, if not entirely revolutionary in terms of methods employed or production titres achieved for the same products in related hosts. However, the master rectory system is elegant and seeks the better match regulatory control with a higher production titles required in biotechnology. A major concern I have is readability, framing of the research the transparency within which this study is place within the wider body of literature.

Response:

- ✓ Thank you for your constructive evaluation and encouraging comments on our regulatory system. We appreciate your feedback on the manuscript's readability, framing, and positioning within the broader literature, and agree that addressing these points will substantially strengthen the work.
- ✓ In the revised manuscript, we have undertaken a comprehensive revision to enhance narrative flow and accessibility. We have reframed the **Introduction** and **Discussion** to more explicitly articulate our research rationale and specific contributions.
- ✓ Furthermore, we have expanded the literature review to provide a more transparent background, ensuring our findings are clearly situated within the existing body of knowledge. We believe these revisions significantly improve the clarity of our study. We are grateful for the time and expertise you have dedicated to its evaluation.

Major:

1. The manuscript throughout claims the research study is a lignin valorisation strategy whereas in reality this should be referred to as a lignocellulosic valorisation strategy. Indeed, the phenylpropenoic acids utilised are only yielded in small quantities from the hemicellulose fraction of lignocellulosic biomass, where is in the actual lignin fractions the G, H and C type monomers are cross-linked through carbon-carbon and carbon ether bonds. Neither thermochemical or enzymatic deconstruction yields any significant proportion of these phenyl propanoic acids. This should be revision throughout

Response:

- ✓ Thank you for this insightful comment. We apologize for the imprecise terminology used to describe our study. We agree that the phenylpropanoids

utilized are released in small quantities from the pendant groups linking lignin and hemicellulose of lignocellulosic biomass. Therefore, directly referring to their conversion as 'lignin conversion' is technically inaccurate, and greater precision in terminology is essential to avoid potential misunderstandings.

- ✓ In response, we would like to revise the manuscript to more accurately clarify the origin of the phenylpropanoids used. However, we are concerned that adopting the term "lignocellulose valorization" might introduce further ambiguity, as it encompasses the conversion of cellulose and hemicellulose—fields that are more established and distinct from the aromatic-focused scope of our work. To ensure terminological precision, we consulted recent high-impact literature. Previous studies establish the convention of referring to the bioconversion of lignin-related aromatic compounds, such as *p*-coumaric acid and ferulic acid from alkaline pretreatment liquors, as "lignin conversion," even if they are not direct units of lignin polymer[1-3]. In this broader context, "lignin conversion" is used to describe the upgrading of lignin-rich or lignin-derived streams. Accordingly, we have systematically replaced "lignin-derived aromatics" with "lignin-related aromatics" throughout the manuscript. Additionally, we clarified phenylpropanoids origins in the **Introduction**, analyzed their actual content in the **Discussion**, and presented our outlook for future studies. The specific revisions are as follows：
- ✓ We have replaced "lignin-derived aromatics" with "lignin-related aromatics" to more accurately reflect that phenylpropanoids are not directly derived from the lignin polymers. These changes have been implemented in the **Title**, **Abstract**, **Introduction** and throughout the manuscript.

  The title has been revised to: "Engineering a Robust and Autonomous Biological Funneling for Efficient Valorization of Lignin-Related Aromatics".

  "Phenylpropanoids are ester pendent-linked to lignin and hemicellulose rather than core-integrated to lignin. Often counted as part of total lignin in corn stover, their valorization is crucial for lignin utilization as they are efficiently monomerized via alkaline depolymerization" was added to **Introduction** (Line 55-58).

  "… phenylpropanoids constitute only a minor fraction of the lignocellulosic complex, typically 3%–4% by weight, their biological significance is disproportionately amplified by their susceptibility to release via alkaline depolymerization …." was added to **Discussion** (Line 725-728).

2. The introduction seems overly focused on championing the chosen host organism C.

glutamicum as a host for 'lignin valorisation'. As written it seems to suggest that this is the first time that C. glutamicum has been used for aromatic catabolism into products which is untrue as there is extensive literature from the Wittman laboratory on the same topic. Indeed, higher muconic acid titres have previously reported from the same, in excess of 80 g/L from lignin-derived aromatics (10.1186/s12934-018-0963-2)

Response:

- ✓ We sincerely thank you for this important and insightful comment. We apologize that our previous Introduction did not adequately acknowledge prior advancements in *C. glutamicum*-based aromatic bioconversion, particularly the extensive work conducted by the Wittmann group. We also agree that the original Introduction have placed disproportionate emphasis on highlighting *C. glutamicum* as a host for aromatic compound conversion, which could inadvertently overstate its novelty.
- ✓ In response, we have revised the **Introduction** to more accurately position our study within the existing literature. Specifically, we have incorporated and explicitly acknowledged prior foundational studies, including those from the Wittmann laboratory and others, demonstrating the capability of engineered *C. glutamicum* to convert aromatics into products such as muconic acid at high titers. We have also moderated statements that could overstate novelty or imply a first demonstration. Specific revisions include:

  "In addition, research on *Corynebacterium glutamicum* for lignin valorization has attracted increasing attention" was added (Line 71-73).

  "*C. glutamicum* … serves as a superior microbial chassis for lignin biorefinery applications" have been revised to "*Corynebacterium glutamicum* has emerged as a promising chassis for lignin valorization due to its intrinsic aromatic catabolic capacity, robust metabolism, and industrial applicability." (Line 82-83).

3. Further the tone of the introduction is written in quite a colloquial / unprofessional manner flagging the *C. glutamicum* performs "the star strain *Pseudomonas putida*" Further no reference is given to all that several other bacterial strains have been reported to display aromatic/lignin monomer catabolic capabilities such as *Comanonas sp*. and *R josti*. I suggest that the authors review the extensive literature on these topics specifically from the Bugg, Eltis, Masai, Aristilide labs

Response:

- ✓ Thank you for this valuable comment. We agree that the tone of the original Introduction was not sufficiently formal and the discussion of microbial platforms

for lignin valorization lacked adequate breadth.

- ✓ In response, we have carefully revised the Introduction to adopt a more professional and balanced tone. In addition, we have expanded the literature coverage to better reflect the diversity of microbial hosts capable of aromatic and lignin monomer catabolism. We now explicitly include representative strains such as *Comamonas testosterone* and *Rhodococcus jostii*, and have incorporated relevant studies from the Bugg, Eltis, Masai, and Aristilde research groups to provide a more comprehensive overview of the field. These revisions have been made throughout the **Introduction** to ensure a more accurate and well-supported presentation. Specific revisions include:
- ✓ We have removed colloquial expressions such as "star strain" and avoided comparative statements that may overemphasize one organism over others.

  "Natural and engineered … valorization" has been revised to "A variety of microbial hosts, including *Pseudomonas putida, Rhodococcus jostii*, *Escherichia coli*, *Comamonas testosterone*, *Sphingomonas paucimobilis* SYK-6, and *Cupriavidus necator*, have been used as platforms for lignin-related aromatics conversion" (Line 68-71).

  The relevant studies from the Bugg, Eltis, Masai, and Aristilde labs have been appropriately cited in the main text.

4. The manuscript title and throughout reports the development of microbial hosts, whilst such hosts could utility within biorefinery, the host alone is neither the biorefinery or platform in themselves. So, the authors to tone down the claims that this is now lignin biorefinery platform

Response:

- ✓ Thank you for this important and insightful comment. We apologize for overstating microbial hosts as biorefinery platforms. We fully agree that an engineered microbial host or regulatory system does not constitute a complete biorefinery platform.
- ✓ In response, we have carefully revised the manuscript to moderate the scope of our claims and to more accurately distinguish between the engineered microbial system and the broader concept of a lignin biorefinery. Specifically, we have avoided referring to the engineered strains or the layered and multi-input autonomous system as a "biorefinery platform" and instead described them as an engineered microbial system or regulatory framework. These changes have been

implemented in the **Title**, **Abstract**, **Discussion**, and throughout the manuscript to ensure conceptual clarity and to avoid overstatement. Representative revisions include：

The **Title** has been revised to: "Engineering a Robust and Autonomous Biological Funneling for Efficient Valorization of Lignin-Related Aromatics"

"lignin biorefinery" has been revised to "lignin-related aromatics valorization" (Line113)

"lignin biorefinery" has been revised to "lignin-related aromatic valorization valorization" (Line 599).

"lignin biorefinery microbial factories" has been revised to: "microbial platforms" throughout the whole **Discussion**.

- ✓ We believe these revisions improve the precision and clarity of the manuscript and better align our claims with the scope of the work.

5. A major challenge with utilising biomass derived feedstocks is the complex variable and a heterogenous composition - therefore did the authors explore the performance of the master system under different compositions for example decreasing ferulic acid and increasing coumaric acid? This would strength the claims around industrial utility

Response:

- ✓ We sincerely thank you for this valuable suggestion, which has significantly strengthened the rigor and practical relevance of our study. We agree that evaluating the performance of the autonomous regulatory system under varying substrate compositions is highly informative and strengthens our claim regarding its industrial applicability.
- ✓ In response, we performed additional fermentation experiments using mixed phenylpropanoid substrates with systematically varied ratios of *p*-coumaric acid and ferulic acid and integrated the resulting data into the revised manuscript with detailed analysis. Furthermore, we expanded the Discussion to highlight the complexity and heterogeneity of biomass-derived feedstocks as a key challenge for industrial bioconversion and to clarify how our system addresses this issue in the context of recent advances.
- ✓ The newly generated data and corresponding descriptions have been incorporated into the main text. Results are presented in **Figure7h** (and below). Specifically:

  "Given that biomass-derived feedstocks are inherently complex, variable, and heterogeneous, we next evaluated the compositional adaptability of SASR7.

Specifically, we tested its performance across a range of p-CA/FA/CaA ratios to simulate varying substrate compositions. Remarkably, SASR7 consistently maintained conversion efficiencies above 99.9% under all tested conditions (Fig. 7h), demonstrating its strong robustness and ability to adapt to compositional fluctuations in lignin-derived feedstocks." was added into the main text (Line 521-527).

"A major challenge in utilizing biomass-derived feedstocks lies in their complex, variable, and heterogeneous composition. The robustness of this design is further highlighted by its ability to maintain high performance (conversion efficiencies exceeding 99.9%) across varying substrate compositions, demonstrating strong adaptability to the inherent variability of lignin feedstocks." was added into Discussion (Line 697-702).

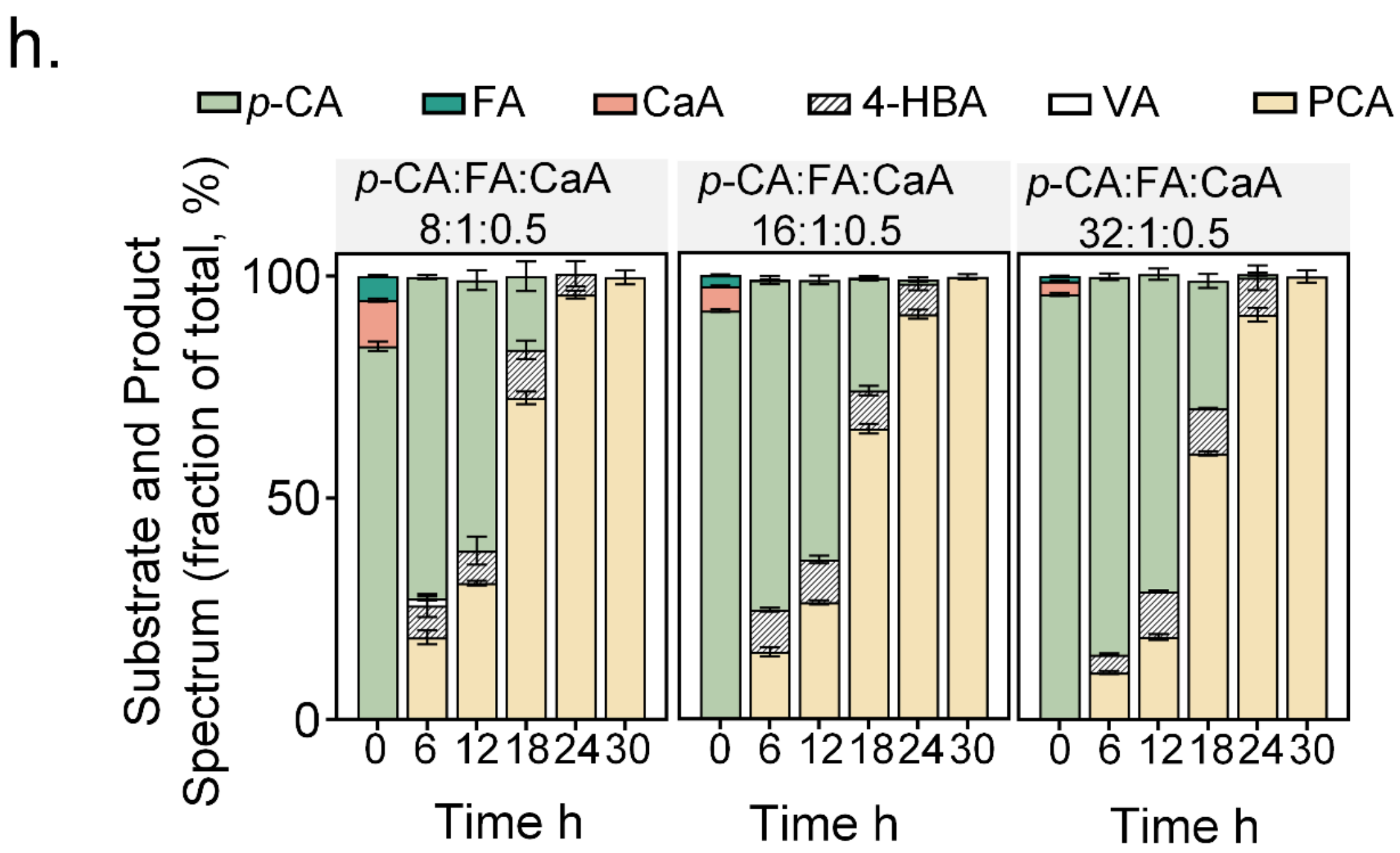

Fig 7h, Fermentation profiles of strain SASA7 under varying phenylpropanoid substrate ratios. Stacked bars show normalized fractions of substrates (*p*-CA, FA, CaA), intermediates (4-HBA, VA), and product (PCA) at indicated time points (all concentrations normalized to total substrate input; sum = 100%). Three feeding conditions (total mM): 63.21 (53.21 *p*-CA, 6.57 FA, 3.42 CaA), 62.69 (57.71 p-CA, 3.42 FA, 1.56 CaA), and 62.00 (59.44 *p*-CA, 1.82 FA, 0.73 CaA). Colors are defined in the legend.

6. It would also be useful to benchmark the performance of the master control system against an unregulated or natively regulated system for example does this complex genetic control circuitry offer advantages above constitutive expression of all genes at once?

Response:

- ✓ We sincerely thank you for this insightful and constructive suggestion. We fully agree that it is important to compare the autonomous regulatory system not only with the native regulated system but also with a constitutive expression of all genes. In our original manuscript, we only compared the native regulatory strain (SHT1) with the layered multi-input regulatory strain (SASR7).
- ✓ To address this shortcoming, we constructed an additional strain based on SHT1, in which all pathway genes are constitutively expressed, designated SHTCE. To ensure a fair comparison and minimize potential confounding factors, we employed a genome integration strategy rather than plasmid-based expression, thereby avoiding plasmid-associated metabolic burden. Specifically, the native promoters were replaced with constitutive promoters of matched strengths, enabling constitutive expression of all relevant genes (schematic illustration of the genome integration strategy for SHTCE is shown in Fig 10a). We then performed fermentation experiments using SHT1, SHTCE, and SASR7. The corresponding results and their descriptions have been incorporated into the revised manuscript, further demonstrating the superior performance of the autonomous regulatory system over both native regulated system and constitutive expression. Due to space limitations in Fig. 7, these data are presented in Supplementary Fig. S10, and the following description has been added:

  "To further benchmark this multiplexed dynamic control strategy against conventional approaches, we constructed strain SHTCE, in which all funneling genes (*phdAEBC*, *vanAB and pobA*) were constitutively overexpressed. Although SHTCE retained the baseline conversion capacity of SHT1, its overall performance remained inferior to SASR7 (Supplementary Fig. 10), demonstrating that static overexpression is insufficient to achieve efficient flux coordination. These results highlight the advantage of the layered and multi-input autonomous dynamic control system in achieving balanced and efficient metabolic conversion." (Line 512-520).

  "In contrast to constitutive expression systems, this design dynamically aligns pathway flux with substrate input and intermediate accumulation, thereby minimizing metabolic burden and preventing the buildup of toxic intermediates." (Line 686-689).

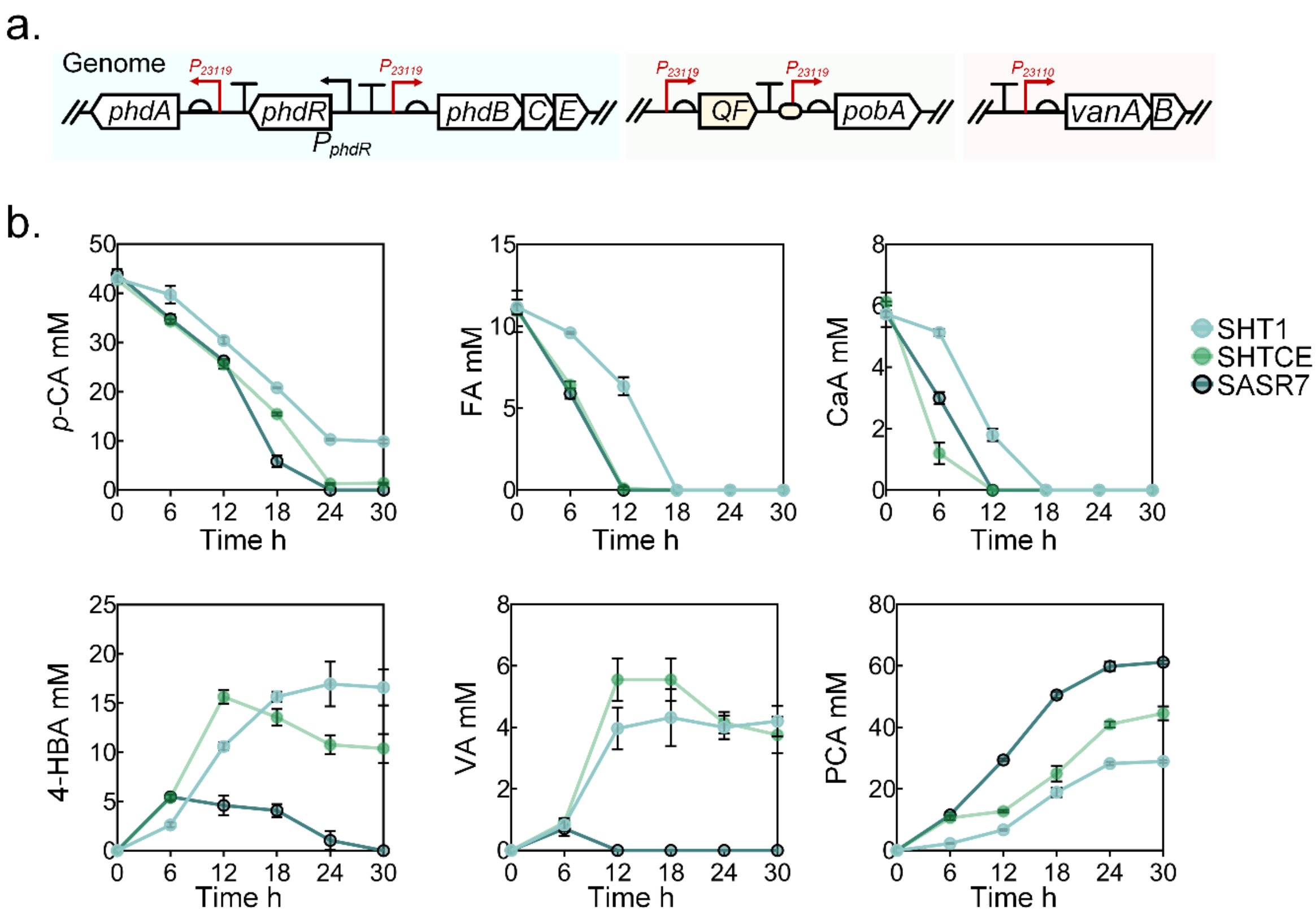

Supplementary Fig. 10 Construction of SHTCE and comparative fermentation performance with SHT1 and SASR7.

(a) Schematic illustration of the genome integration strategy for SHTCE. Native promoters of the target gene clusters were replaced with constitutive promoters of defined strengths to enable constitutive expression of all biological funneling genes (*phdAEBC*, *vanAB*, and, *pobA*). The specific promoters used are indicated in red in the schematic. Regulatory elements, gene organization, and integration loci are shown as depicted.

(b) Time-course fermentation profiles of strains SHT1, SHTCE, and SASR7 cultivated with a mixed phenylpropanoid substrate containing of *p*-coumaric acid (*p*-CA), ferulic acid (FA), and caffeic acid (CaA) at a molar ratio of 4:1:0.5. Residual substrate concentrations (*p*-CA, FA, and CaA), intermediate metabolites 4-hydroxybenzoic acid (4-HBA) and vanillic acid (VA), and the final product protocatechuic acid (PCA) are shown over time. Data points represent mean ± s.d. (n = 3).

7. Discussion I found this part of the manuscript to be very inward looking and was essentially just a summary of the results with only a small scattering of references. This section could do with an extensive rewrite placing the work more fully in the context of the surrounding literature discussing openly and frankly what other titres have achieved for muconate and related products in this host and other hosts, and the need to push production titles as well as expanding microbial metabolism to utilise lower cost feedstocks such as aromatics. This section could also do with a frank rationalisation of the concentration of the phenylpropenoic acids utilised as feedstocks here generally

only present in 3 to 4% maximum of lignocellulosic biomass (in their ester linked form) whereas lignin deconstruction releases a different class of aromatic momomers.

Response:

- ✓ We are deeply grateful for your constructive feedback and the viable directions suggested for our discussion, which have significantly enhanced the quality and depth of the manuscript. We acknowledge that the original text was overly inward-looking, failing to adequately contextualize our work within the current state of the field and its comparative relevance to other studies. Following your suggestions, we have completely rewritten the **Discussion** section (Line 616–736) to provide a more rigorous and comprehensive analysis. Our revisions focus on the following several key aspects:
- ✓ (I) **Performance evaluation and the shift toward low-cost feedstocks:**
  We have expanded our literature review to place our work within the global landscape of muconate and related products production. We also discussed the importance of improving production titers and expanding microbial metabolism to utilize low-cost, sustainable feedstocks include aromatics. For example:
  "Currently, engineered *C. glutamicum* … has achieved remarkable titers from glucose, including 82.7 g L^{-1} PCA and 88.2 g L^{-1} CCM. Notably, it produced 85 g L^{-1} CCM from catechol, the highest reported titer from lignin-related aromatics to date. Given its established research foundation and superior metabolic plasticity, applying *C. glutamicum* to biomanufacturing oriented toward low-cost, renewable substrates—such as lignocellulose-related aromatic mixtures or other inexpensive carbon sources—holds great promise for enhancing the economic viability and environmental sustainability of the entire process. … process optimization remains a clear and viable trajectory for further titer enhancement. This is exemplified by pioneering research where, through the optimization of feeding strategies, the current record for β-KA production from alkaline-pretreated corn stover-derived aromatics was established in *P. putida*" (Line 704-722)
- ✓ (II) **Rationalization of phenylpropenoic acid utilization:**
  We have provided a frank rationalization regarding our focus on ester-linked phenylpropanoids, explicitly acknowledging that these compounds constitute only a small fraction (at most 3–4%) of lignocellulosic biomass. Furthermore, we have elaborated on the strategic rationale for selecting these phenylpropanoids as our target feedstocks and noted that future research should focus on expanding the substrate spectrum of our engineered strains to valorize a broad range of lignin-

derived monomers. For example:

"Although phenylpropanoids constitute only a minor fraction of the lignocellulosic complex, typically 3%–4% by weight, their biological significance is disproportionately amplified by their susceptibility to release via alkaline depolymerization. Unlike the recalcitrant core lignin polymer, these pendant groups are readily dissociable, thereby providing an accessible substrate flux for engineered microbial platforms. However, the industrial lignin valorization necessitates a heightened level of metabolic plasticity." (Line 725-731)

- ✓ (III) **Other discussions:**

  Other discussions regarding the connection and comparison between our work and the existing research landscape—such as laboratory adaptive evolution and biosensor development—have also been integrated. The entirely revised Discussion section can be found in the updated manuscript (Lines 616–736).

8. Lots of strains used with lots of completed nomenclature, provide a table with clear description with names, nodes and components changed description. Similarity figure legends do not provide all the key information including conditions used

Response:

- ✓ Thank you for this constructive comment. In the revised manuscript, we have improved the presentation of strain information and figure legends.
- ✓ We have included a concise table in the main text to summarize the key strains and their corresponding construction details (**Table 1. Selected strains used in this study**). A comprehensive table summarizing all strains, including nomenclature, genetic modifications, and descriptions, has been reorganized in **Supplementary Table 1** with enhanced clarity and accessibility.
- ✓ In addition, all relevant figure legends have been revised to include complete experimental details (e.g., cultivation conditions and substrate concentrations), ensuring that the figures are self-explanatory and reproducible. These changes address the previous lack of clarity and significantly improve the usability of the manuscript.

Strain	Characteristics	Reference
C. glutamicum ATCC 13032	Wild-type strain	Laboratory stock
Spca1	*C. glutamicum* ATCC 13032 with *pcaHG* gene deletion	This study
Spca2	Spca1 carrying pEC-XK99E-P_{trc}-*phdA-phdE-phdB-phdC*	This study
Spca3	Spca1 with phdR gene deletion	This study
Spca4	Spca2 with *phdR* gene deletion	This study
Spca5	Spca4 carrying pEC-XK99E-Ptrc-VanABCgl	This study
Spca6	Spca4 with *vanR* gene deletion	This study
Spca7	Spca6 carrying pEC-XK99E-P_{trc}-*VanAB*^{Cgl}	This study
Spca8	Spca4 carrying pEC-XK99E-P_{trc}-*PobA*^{Cgl}	This study
STALE9	Evolved strain-3 derived from ALE with *p*-CA	This study
SHT1	STALE9 with *pcaHG* gene deletion	This study
SASR1	SHT1 with P_{phdB} replcaced by $P_{bba\text{-}23110}$-O2-B0034	This study
SASR2	SHT1 with P_{phdB} replcaced by $P_{bba\text{-}23106}$-O2-B0034	This study
SASR3	SHT1 with P_{phdB} replcaced by $P_{bba\text{-}23119}$-O2-B0034	This study
SASR4	SASR3 with P_{pobA} replaced by P_{pobA}-QF-*QUAS*-$P_{bba\text{-}23110}$	This study
SASR5	SASR3 with P_{pobA} replaced by P_{pobA}-QF-*QUAS*-$P_{bba\text{-}23106}$	This study
SASR6	SASR3 with P_{pobA} replaced by P_{pobA}-QF-*QUAS*-$P_{bba\text{-}23119}$	This study
SASR7	SASR6 with P_{vanA} replaced by $P_{Bba\text{-}23110}$-O_{van}	This study
SASR8	SASR6 with P_{vanA} replaced by $P_{Bba\text{-}23106}$-O_{van}	This study
SASR9	SASR6 with P_{vanA} replaced by $P_{Bba\text{-}23119}$-O_{van}	This study
SASR7-C1	SASR7 with *catBC* deletion and genomic intergration of $P_{bba\text{-}23119}$-*aroY-ecdB-ecdD*	This study
SASR7-K19	SASR7 with *pcaIJ* deletion and genomic intergration of $P_{Bba\text{-}23119}$-*pcaHG*	This study

Table 1. Selected strains used in this study.

Table S1 shows the complete strain list and corresponding construction details.

Minor

L19 – meaning unclear. What are downstream metabolic capabilities presumably that refers to central metabolism whereas the focus here is on upper catabolic pathways for this assimailt of aromatics into central metabolism.

Response:

✓ Thank you for this insightful comment. We apologize for the lack of linguistic precision in our previous draft. The phrase "downstream metabolic capabilities" was originally intended to describe the upper catabolic pathways that funnel lignin-related aromatics into central metabolic intermediates, rather than central metabolism itself.

✓ To eliminate ambiguity, we have revised the **Abstract**, removing potentially misleading terminology to provide a more precise overview of our work. The first sentence has been updated to: "Microbial conversion of lignin-derived aromatics

is fundamentally constrained by substrate toxicity and metabolic flux imbalance." (Line 19-20).

L24 – here ALE was employed to generate…

Response:

✓ We sincerely thank you for this careful insight. Accordingly, we have revised the sentence to "Adaptive laboratory evolution was employed to generate a robust chassis…" (Line 23-24).

L30 – claims that these are their highest reported titles for muconic acid from lignin derived aromatics however Becker et al have reported muconic acid titres in excess of 80 g/L from lignin-derived aromatics (10.1186/s12934-018-0963-2

Response:

✓ We sincerely thank you for this careful and important comment. We agree that our original statement regarding the "highest reported titer" was not sufficiently precise, the study by Becker et al. reports 85 g L^{-1} muconic acid from catechol. To avoid potential overstatement and ensure accuracy, we have revised our wording to more carefully contextualize our results.

✓ Specifically, we have removed the claim of the "highest reported titer" (Line 30).

L46 "lignin" not "Lignin"

Response:

✓ We sincerely thank you for this careful and attentive comment. We have corrected "Lignin" to "lignin" in the revised manuscript.

L54 "have evolved"

Response:

✓ We sincerely thank you for this careful and helpful suggestion. We have revised the phrasing to "have evolved" to ensure grammatical accuracy and clarity.

L63 "pivotal breakthrough to the commercialization of lignin" this claim is unsupported. Boregard is the only established lignin-based biorefinery, so how this native catabolic capability a breakthrough? In addition, phenyl propanoic acids are only yielded in small quantities from the hemicellulose fraction of lignocellulosic biomass, where is in the actual leg infractions G, H and C type monomers they are cross-linked through carbon-

carbon and carbon ether bonds. Neither thermochemical or enzymatic deconstruction of leading yield and a significant fraction of these phenyl propanoic acids.

Response:

- ✓ We sincerely thank you for this insightful and critical comment, as well as for the careful evaluation of the conceptual framing of our study, which have significantly improved the rigor and clarity of the manuscript. We fully agree that the original statement describing our work as a "pivotal breakthrough to the commercialization of lignin" was overstated and not sufficiently supported. We also appreciate your clarification regarding the limited availability of phenylpropanoic acids from lignocellulosic biomass, particularly given their relatively low yields.
- ✓ In response, we have revised the wording to adopt a more cautious and accurate description of the significance of our work, emphasizing its role as a step toward improving microbial utilization of lignin-related aromatics, rather than implying a direct breakthrough in lignin commercialization. Specific revisions are as follows: The original sentence has been revised to: "This funneling strategy provides an inherent solution to lignin heterogeneity by enabling the convergence of multiple substrates into unified metabolic routes." (Line 62-64).

L74 "concentrated on merely a singularly dominant bacterial" variation literature reports on other hosts including *C. glutamicum* and *R. josti*.

Response:

- ✓ We sincerely thank you for this insightful comment. In response, we have revised the sentence to provide a more balanced and accurate description of the field, and we have incorporated the suggested examples where appropriate. Specific revisions are as follows:
- ✓ The original sentence has been revised to: "A variety of microbial hosts, including *Pseudomonas putida*, *Rhodococcus jostii*, *Escherichia coli*, *Comamonas testosterone*, *Sphingomonas paucimobilis* SYK-6, and *Cupriavidus necator*, have been used as platforms for lignin-related aromatics conversion, capitalizing on their species-specific metabolic features. In addition, research on *Corynebacterium glutamicum* for lignin valorization has attracted increasing attention." (Line 68-73).

L84 "despite" not "Despite"

Response:

- ✓ We have corrected "Despite" to "despite" in the revised manuscript. And we also

checked the entire text for spelling errors.

L143 “Layer 2 acts” not “Layer 2 act”

Response:

- ✓ We have corrected “Layer 2 act “Layer 2 acts” in the revised manuscript. And we also checked the entire text for spelling errors.

L146 “to” not “To”

Response:

- ✓ We have corrected “To” to “to” in the revised manuscript. And we also checked the entire text for spelling errors.

L148 “homologous” not “endogenous” to distinguish between plasmid over expression and native chromosomal copies

Response:

- ✓ We sincerely thank you for this careful and insightful suggestion, and for pointing out the importance of precise terminology in distinguishing gene expression contexts. We agree that “homologous” is much more appropriate than “endogenous” when referring to genes expressed from plasmids as opposed to native chromosomal copies.
- ✓ Accordingly, we have revised the wording throughout the manuscript to ensure accurate usage of this term.

L152 “Layer 1” this in canonical referred to as the upper aromatic degradation pathway, terminological consistency strongly advised

Response:

- ✓ Thank you for this insightful comment. We regret that our earlier imprecise language may have led to any misunderstanding. Our intention was to conceptually divide the upper aromatic degradation pathway—specifically from phenylpropanoids to PCA—into two functional layers, designated as Layer 1 and Layer 2, based on the observed accumulation of intermediates (4-HBA and VA) during our experiments. This layered design reflects the sequential conversion steps and facilitates a clearer understanding of the pathway architecture. To avoid any potential confusion, we have clarified this point in the revised manuscript and explicitly stated that the layered terminology applies specifically to the conversion

process from phenylpropanoids to PCA. We hope this addresses your concern. We have provided the expanded definitions and explanations below:

- ✓ "The biological funneling pathways of *C. glutamicum* are generally defined by upper and lower pathways. To elucidate metabolic limitations in the native biological funneling pathway of *C. glutamicum*, in this study, we first delineated the upper biological funneling pathway, which channels phenylpropanoids toward PCA, into two functional layers based on metabolic roles. The first layer (Layer 1) involves β-oxidation of phenylpropanoids, yielding benzoate derivatives and acetyl-CoA, whereas the second layer (Layer 2) comprises the hydroxylation of *p*-hydroxybenzoic acid (4-HBA) and O-demethylation of vanillic acid (VA)." (Line 130-137).

L154 "The results showed that" where are these results?

Response:

- ✓ We sincerely thank you for this careful observation. We are sorry that the original phrasing "The results showed that" was unclear due to the distance from the corresponding figures.
- ✓ To improve clarity, we have revised the text to directly reference the relevant figures and added: "(Fig. 2g–i)" at the point of description (Line 163). We hope this adjustment will help readers easily locate and interpret the supporting data and figure.

L161 "demonstrating that enhancing phd expression improves Layer 1 efficiency at 161 the cost of aggravating intermediate accumulation." I do not believe this statement to be correct, the PCA titre has not decreased, so nothing has been 'aggravated' – this appears that just the 4-HBA (and VA) to PCA node is the bottleneck

Response:

- ✓ We sincerely thank you for this careful and insightful comment. We agree that the original statement suggesting that enhancing phd expression "aggravates intermediate accumulation" was misleading. As correctly noted, PCA titres do not decrease upon phd overexpression; rather, the accumulation of intermediates 4-HBA and VA reflects the bottleneck at the 4-HBA (and VA) to PCA conversion step. Accordingly, we have revised the text to accurately describe the results:
- ✓ "confirming that while strengthening Layer 1 improves upstream flux, Layer 2 remains the dominant rate-limiting step." (Line 169-170).

L174 Refers toxic of the aromatics, the SI fig does not clearly show this ie final OD/growth curves +/- the ligand for the w-t and engineered strains – should this refer to Fig3A instead?

Response:

- ✓ We sincerely thank you for this careful and professional comment. We agree that Supplementary Figure 1 does not include the final OD_{600} with or without the ligand for the wild-type and engineered strains. Specifically, it only shows the final OD_{600} for the wild type without ligand and for Spca1–8 with their respective ligands. While we assume that the metabolic engineering of Spca1–8 does not substantially affect growth under standard conditions without ligand, this figure cannot serve as direct evidence of growth inhibition due to aromatic toxicity. Rather, it only indicates that Spca1–8 display lower final OD_{600} compared to the wild type when cultured with CaA, *p*-CA, and FA.
- ✓ To address this concern, we have revised the text to refer to Fig. 3A and Supplementary Figure 1, which provide a clearer representation of the growth behavior of wild-type strain in the presence and absence of the aromatic ligands.

Fig 2A – The product of the PhdABC pathway is the aromatic aldehyde, not the acid shown, is Vanillin from ferulic acid is the product not vanillic acid

Response:

- ✓ We sincerely thank you for this careful comment. According to theoretical pathways and previous reports[4-6], the product of the *phdABC* pathway in *C. glutamicum* is vanillic acid, consistent with both our experimental observations and Fig. 2A. We have clarified this point in the figure legend and main text to ensure that the depiction accurately reflects the expected and observed product.

Fig 2G "KA" should this be "CaA"

Response:

- ✓ We sincerely thank you for this careful and attentive comment. We have corrected "KA" to "CaA" in the revised manuscript.

L191 "Spca1 fermentation" what fermentation mode, presumably bolus addition of the aromatics based on the panels b-d

Response:

- ✓ We sincerely thank you for this careful comment. We would like to clarify that the Spca1 fermentations were performed using a fed-batch mode with sequential addition of the aromatic substrates (Fig. 2b–d). Thank you for pointing this out; we'll clarify it to avoid misleading our readers.
- ✓ We have updated the figure legend to clearly specify the fermentation mode and substrate addition strategy (Line 198-202).

L214 Although post-ALE genome sequencing was performed, and the commonly occurring mutations were shown, Analysis was carried out/communicated as to what these exact commutations were. This should be included in the revised manuscript, and combined with subsequent transcriptomics analysis performed for STALE9 strain

Response:

- ✓ We sincerely thank you for this constructive suggestion. We fully agree that, to improve the logical rigor of the manuscript, the mutations identified from genome sequencing should be described in greater detail in this section. In the previous version of the manuscript, the genomic mutation data were presented later in the section entitled "Elucidating the synergistic tolerance mechanisms to lignin-derived phenylpropanoids in evolved *C. glutamicum* and reverse genetics validation," and this organization may have affected the flow and logical coherence of the manuscript.
- ✓ To address this issue, we have revised the manuscript by relocating and expanding the description of mutations identified from post-ALE genome sequencing, and by integrating these results with the transcriptomic analysis. The relevant description is in Result "**Elucidating the synergistic tolerance mechanisms to lignin-derived phenylpropanoids in evolved *C. glutamicum* and reverse genetics validation**" (Line 259-289).
- ✓ To provide a comprehensive overview of all genomic mutations identified through sequencing, a detailed summary of the intersectional mutated genes in the STALE9 strain has been included in **Supplementary Table 4**.

L217 "STALE1-STALE9" define

Response:

- ✓ We sincerely thank you for this helpful suggestion to improve the completeness and readability of the manuscript. We are sorry that the original version lacked a

clear definition and description of strains STALE1–9, which could hinder understanding and disrupt the logical flow. To address this issue, we have revised the main text to include explicit definitions of these strains.

"To characterize evolutionary outcomes, three independent clones were isolated from each ALE lineage, generating strains STALE1–3 (*p*-CA), STALE4–6 (FA), and STALE7–9 (CaA)." (Line 225-227).

In addition, comprehensive details regarding STALE1–STALE9, along with all other strains utilized in this study, have been compiled in Supplementary Table 1.

L218 "achieved" not "achieving"

Response:

- ✓ We sincerely appreciate your careful reading and helpful suggestion. We have corrected "achieving" to "achieved" to ensure grammatical accuracy and improve clarity.

L239 "We propose that ALE has endowed STALE9 with toxicity resistance, 239 enabling its normal physiological functions recovery under stress." What evidence is there for toxicity resistance? As earlier comments, it would seem more logical to first analysis the genomic mutation in STALE9.

Response:

- ✓ We sincerely thank you for this insightful and constructive comment, and for highlighting the importance of clearly substantiating the claim of toxicity resistance. We agree that the original statement was not sufficiently supported by direct evidence.
- ✓ In response, we have revised the text to adopt a more cautious interpretation. In addition, as suggested, we have incorporated the genomic mutation analysis of STALE9 and discussed these mutations in conjunction with the observed phenotype and transcriptomic data, providing a more mechanistic basis for the improved tolerance. The relevant description is in Result "**Elucidating the synergistic tolerance mechanisms to lignin-derived phenylpropanoids in evolved *C. glutamicum* and reverse genetics validation**" (Line 259-303).

L320 "derepresses" not "derepress"

Response:

- ✓ We have corrected "derepress" to "derepresses" to ensure grammatical accuracy

and improve clarity.

Fig 5g ITC y-axis data label and units are incorrect 'ΔHG' does not represent a thermodynamic metric, ΔH? Units are measured in 'kcal/mol' not 'Kcal/mol'
The corresponding ligand affinities do not appear to be consistent with the literature being in the 1-10uM range (https://doi.org/10.1093/nar/gkx1055), and indeed the Kd data do not look to be internally consistent, pCA binding curve is looks to be significantly steeper than FA, but the reported Kds are similar. In addition, the CaA does not look to be correctly fitted, the data and fitting parameters were not provided . Finally, the reported molar ratio (1:20 to 1:40) do not appear to be consistent with the known binding stoichiometry, 1:1 or 2:1 of aTFs. As such it would appear that either the protein concentration has been under-estimated or the ligand over-estimated, the protein production reported in the Methods section reports 0.375g/L (~10uM) protein concentration was used but doesn't include a dialysis step post imidazole elution following His-tag purification, so is likely that a much protein lower concentration is present.
have some serious concerns as to how this ITC was performed and analysed

Response:

- ✓ We sincerely appreciate you bringing these oversights in our experimental workflow to our attention and we are sorry for this shortcoming. Furthermore, we are grateful for your insightful suggestions on how to improve the direction of our research. These highly professional comments have helped us improve the rigor and precision of the manuscript. The discrepancies in binding affinity and molar ratio compared to previous studies are indeed potentially confusing
- ✓ First, we are sorry for the error in the labeling of the thermodynamic parameters: the y-axis label and unit were incorrect.

  Specifically, "ΔHG" is not a standard thermodynamic parameter and should be "ΔH," and the unit should be "kcal/mol" rather than "Kcal/mol." We have corrected these errors in the revised **Figure 5g** in the main text.
- ✓ Second, the binding curve for *p*-CA appears much steeper than that for FA, whereas the reported Kd values are similar, and the data fitting for CaA also appears questionable. In addition, the reported molar ratios (1:20 to 1:40) are inconsistent with the commonly observed stoichiometries of transcription factor–ligand interactions (typically 1:1 or 2:1).

  We agree that these results present certain inconsistencies, and we sincerely

apologize for the oversights in our previous experiments. We speculate that this may have resulted from: (i) inaccurate protein concentration determination, as protein concentration was previously estimated by absorbance using NanoDrop; and (ii) a suboptimal titration range that did not reach saturation, thereby affecting the fitting quality.

- ✓ To address these issues, we have re-examined and optimized the entire workflow, including protein expression and purification, protein concentration determination, the titration experiments, and affinity determination via ITC data fitting to revalidate the binding affinities of PhdR with *p*-CA, FA, and CaA. The updated titration results are now shown in **Fig. 5g**. We also provide the raw data including fitting parameters of newly performed titration experiments in the **The raw data sheet to Reviewer 2** in the attachment.

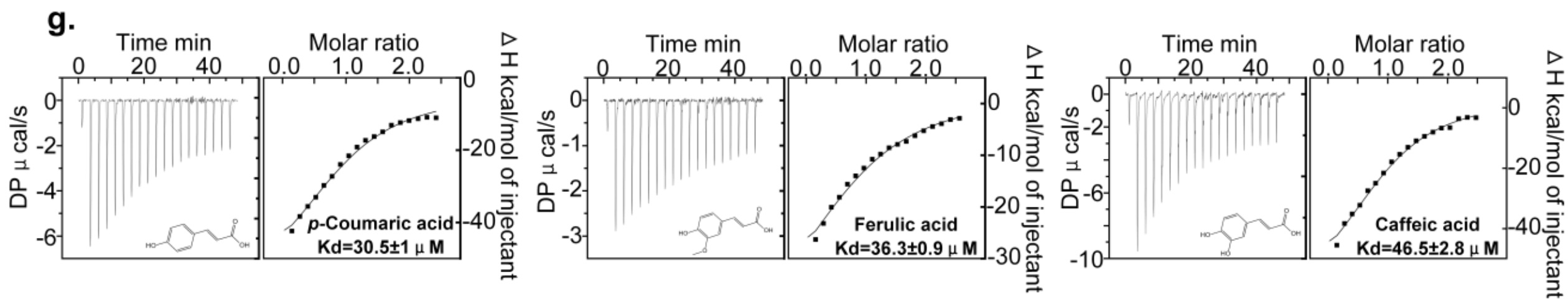

Fig 5g, PhdR affinity measurements for *p*-CA, FA, and CaA. 1.05 g L^{-1} PhdR was used for titration with 0.125 g L^{-1} p-coumaric acid, 0.77 g L^{-1} PhdR was used for titration with 0.115 g L^{-1} ferulic acid, and 1.72 g L^{-1} PhdR was used for titration with 0.23 g L^{-1} caffeic acid. Binding isotherms was fitted using the One Set of Sites model.

- ✓ In addition, we have expanded the **Materials and Methods** section to improve experimental transparency. Specifically, we have added the SDS-PAGE gel image of purified PhdR (Supplementary Fig. 11a), the standard curve for BCA protein concentration determination (Supplementary Fig. 11b), and a detailed description of the ITC titration protocol, including the model used for data fitting (**One Set of Sites**). These additions have been incorporated into Lines 883-901 (protein purification) and Lines 902–918 (ITC assay).

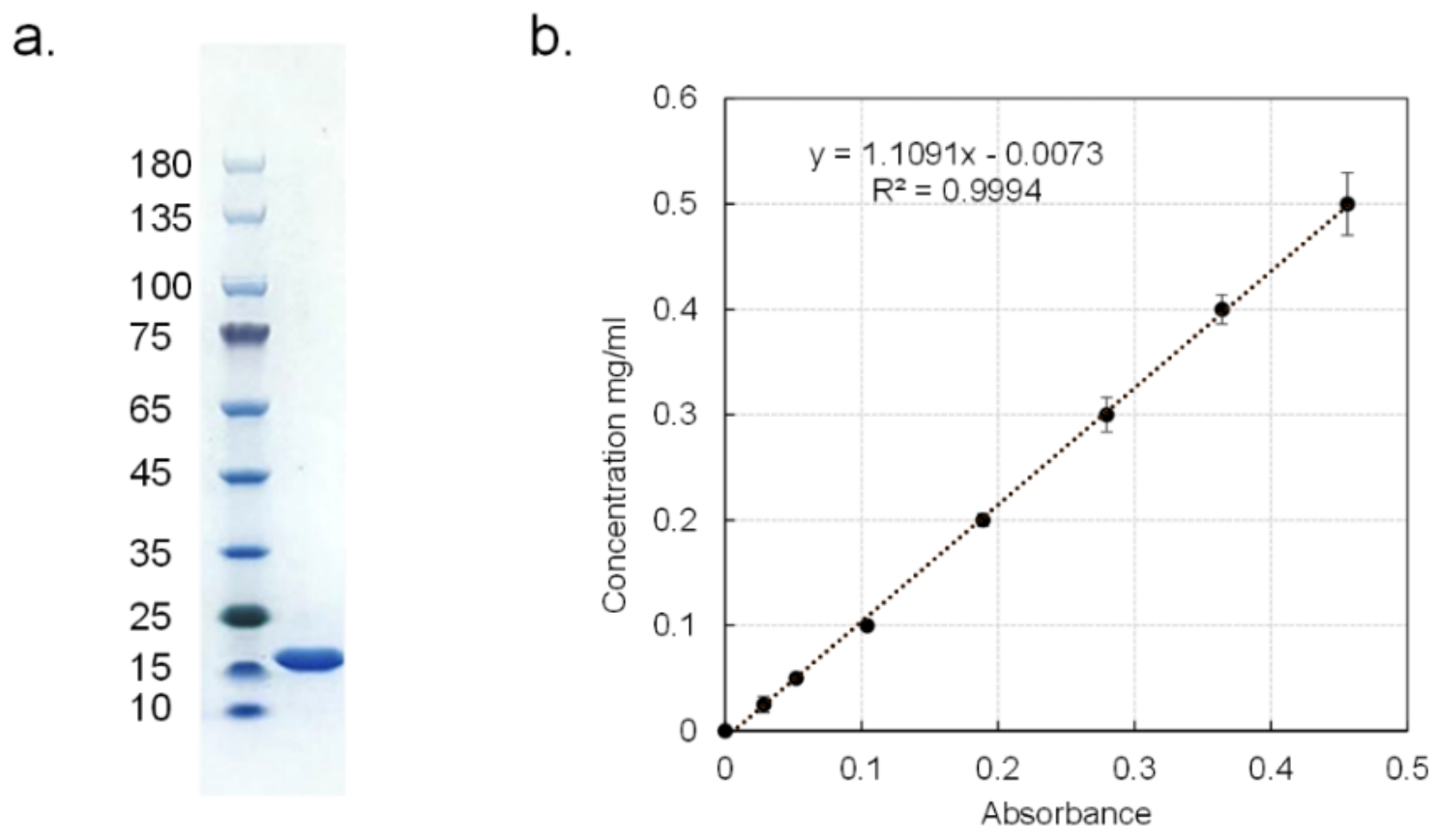

Supplementary Fig. 11 Verification and quantification of PhdR protein purification. a, SDS-PAGE analysis of PhdR after multi-step purification; b, Standard curve of the BCA protein quantification kit, with the absorbance at 562 nm on the x-axis and the protein concentration on the y-axis.

Fig 6 why in vitro Kd and operating range are so different, mM very higher for regulatory proteins

Response:

✓ We sincerely thank you for this insightful and technically important comment, which has helped improve the depth of our manuscript. We agree that the observed millimolar operating range appears relatively high for transcriptional regulators when compared to the in vitro Kd values. We speculate that this discrepancy may arise from several factors:

First, the transport of aromatic compounds from the extracellular environment into the cytoplasm is not fully efficient, meaning that the externally supplied ligand concentration does not directly reflect the intracellular concentration sensed by the regulatory proteins.

Second, differences between in vitro and in vivo conditions—such as protein expression levels, ligand accessibility, cellular context, and potential metabolic turnover of the ligands—can also contribute to an apparent shift in the effective operating range. In addition, similar observations have been reported for related regulatory systems. For example, VanR, a transcriptional regulator in *C. glutamicum* with functional similarity to PhdR, has been shown to require millimolar concentrations of vanillic acid to elicit a measurable response in vivo when using a fluorescent reporter system[7].

✓ To address this, we have added a discussion of this discrepancy in the revised manuscript, including possible mechanistic explanations and relevant literature

references. We hope this addition provides a clearer interpretation of the observed differences between in vitro binding affinity and in vivo operating range. The added discussion is as follows:

- ✓ "our results revealed a significant in vitro-in vivo discrepancy, as the dissociation constant of PdhR measured in vitro was approximately 30–46 μM while the effective response range characterized by in vivo fluorescence assays shifted to the millimolar scale. This phenomenon, commonly observed in biosensor systems, likely arises from limitations in transmembrane transport that constrains intracellular ligand availability, and transcription factor expression levels." (Line 675-680).

F9g c-d and lgends not clear what barcharts represent biosensor dynamic range +/- ligand?

Response:

- ✓ Thank you for pointing out the lack of clarity in Fig. 6 c–e and the corresponding legends. In the revised manuscript, we have clarified that the bar charts represent the biosensor dynamic range, defined as the relative fluorescence change in the presence (+ ligand) versus absence (– ligand) of the target compound. The values correspond to fold change between ligand-induced and uninduced conditions.
- ✓ Specifically, we have revised the legends of **Figure 6** (Line 450-462) to explicitly describe the experimental conditions for +/– ligand and what each bar represents.
- ✓ We hope these revisions improve the interpretability of the figure and eliminate potential ambiguity.

L433 define"QF"

Response:

- ✓ Thank you for this constructive suggestion. We agree that the origin and rationale of the QF-based regulatory module should be clearly described to improve clarity and reproducibility. In response, we have added a concise description in the revised manuscript to explicitly state the source and functional role of the QF/QUAS system. Specifically, description have been added as:
- ✓ "we implemented a QF-mediated transcriptional amplification circuit based on the orthogonal QF/QUAS system, where QF, a transcriptional activator derived from *Neurospora crassa*, enables signal amplification by driving downstream gene expression" (Line 434-437).

L531 “No previous studies have reported CCM production from 531 lignin-derived phenylpropanoids in C. glutamicum” recently reported here https://doi.org/10.1016/j.ymben.2025.08.004”

Response:

✓ Thank you for highlighting this recent study. We note that this work was published during the preparation of our manuscript. We have now incorporated this reference into the revised manuscript and updated the corresponding statement to accurately reflect the current literature. Specifically, we now clarify that CCM production from lignin-related phenylpropanoids in *C. glutamicum* has been recently reported. We further position our work in this updated context by emphasizing its distinct features, including layered and multi-input autonomous dynamic control strategy. Description have been revised as:

✓ “While *C. glutamicum* has been engineered for CCM production from simple aromatics like catechol, recent efforts using lignin-related phenylpropanoids have faced challenges with low titers and insufficient regulatory control” (Line 559-562).

L547 “yielding nylon-6,6 analogs with 546 superior moisture barrier properties” add reference

Response:

✓ Thank you for this helpful suggestion. We agree that a reference is needed to support this statement. Accordingly, we have added an appropriate citation in the revised manuscript (Line 576) to substantiate the reported properties of nylon-6,6 analogs.

Fig 8c chemical structure contain errors “OH-aromatic” shown rather than HO-aromatic for PCA and bKA

Response:

✓ Thank you for highlighting the inaccuracies in Fig. 8c. The hydroxyl group on the aromatic ring has been corrected to “HO-aromatic” for PCA and β-KA in the revised figure.

Fig 8e did the authors observe any difference in cell growth under these different strains for example vanAB-mediated demethylation is known to be burdensome upon cell growth, do any differences in cell growth explain the different performance traits

observed.

Response:

- ✓ Thank you for this constructive comment. We initially observed that plasmid-based overexpression of VanAB imposed a significant growth burden on the host strain, which was accompanied by a relatively slow metabolic rate for vanillic acid compared to the degradation of 4-HBA mediated by PobA (Fig. 1e and 1f). To mitigate this issue, we subsequently integrated a single copy of the autonomously regulated *vanAB* cassette into the genome. This genome-integrated configuration not only eliminated the metabolic burden associated with plasmid maintenance but also ensured a moderate expression level. As a result, the previously observed growth inhibition was largely relieved. Consequently, no significant growth differences were detected among the strains tested in the fermentation experiments shown in Figures 7 and 8.
- ✓ Regarding the different performance traits observed, we concur with your observation. As shown in Figures 8d, 8e, and 8f, the final biomass (OD_{600}) reached during the production of the three target compounds in 5-L bioreactors exhibited noticeable variations: 48.6 for PCA, 45.9 for CCM, and 40.8 for β-KA. These differences in biomass, potentially coupled with the varying metabolic complexity of the respective pathways, likely contributed to the observed disparities in product titers. We appreciate this insightful point and will prioritize these factors in our future efforts to further optimize production levels.

Supplementary Figure 1 – no concentrations provided for the added aromatics

Response:

- ✓ Thank you for pointing this out. In the revised legend of Supplementary Figure 1, we have added the specific concentrations of all aromatic compounds used in the experiments, ensuring clarity and enabling reproducibility. Legend have been revised as:
- ✓ "Total substrate inputs supplied in five sequential pulses over 56 h for respective strains: CaA was supplied in strains Spca1–Spca4, with total inputs of 45.45, 46.35, 47.68 and 47.22 mM, respectively. (light green bars). Total *p*-CA inputs were 48.65, 48.22, 48.00, 47.98 and 48.27 mM for strains Spca1–Spca4 and Spca8, respectively (dark brown bars). Total FA inputs were 43.48, 43.20, 43.35, 43.72, 43.30, 43.72 and 42.82 mM for strains Spca1–Spca7, respectively, (pink bars)."

Reviewer #3:

General Comments:

Microbial bioconversion of lignin is an important part of the overall effort to valorize lignocellulosic biomass, as evident from the surge of recent literature reports on this topic. However, this is not an easy task due to the recalcitrant nature of lignin as well as the toxic nature of the aromatics obtained from depolymerized lignin. Most of the metabolic engineering efforts to valorize lignin have predominantly focused on Pseudomonas putida as the host strain, although there also quite a few reports on the use of engineered *Corynebacterium glutamicum* for this purpose, notably from Christopher Wittman's group (e.g. see Becker et al. MCF 2018; Weiland et al. Met Eng. 2023 etc.). So, the authors of this manuscript are not doing something novel by employing *C. glutamicum* as the host strain. However, the authors have demonstrated certain unique approaches to rational metabolic engineering of *C. glutamicum* and managed to engineer strains which can consume all three major lignin-related aromatics (caffeate, *p*-coumarate and ferulate) and demonstrate production of reasonably high titres of protocatechuate (PCA) and muconic acid (MA) at near 100% conversion.

To begin with, they successfully evolved the native strains by adaptive laboratory evolution (ALE), in order to tolerate high concentrations of the toxic substrates. After identifying critical genetic changes in the genome sequence of the evolved strains, they have reverseengineered the wild-type strains to take up higher concentrations of the substrates and efficiently channelled them towards the desired metabolites. Furthermore, they identified and engineered the allosteric transcription factors (aTFs) which bind to the substrates, as well as the cognate promoter systems, which has enabled the development tunable expression systems (aromatic response regulators) for controlling the metabolic fluxes. Using this strategy, they have efficiently de-bottlenecked the pathways to avoid accumulation of inhibitory intermediates and channelled all the substrates towards accumulation of the key intermediate, viz. PCA. PCA can be produced as the main product by knocking out the downstream genes as well as channelled towards the production of MA by overexpressing the *aroY* gene.

Using the engineered strains, the authors developed fed-batch processes to obtain high titres of the products in lab-scale bioreactors. These kind of titres have been reported in literature using other strains as well as by using sugars as substrates along with lignin. In their study, the authors have used glucose as a co-substrate along with lignin-related aromatics. However, it is not very clear if the utilization of glucose (presumably only

for biomass production) is completely de-coupled from the utilization of aromatics (which are presumably used only for product formation). Glucose can be potentially channelled toward formation of PCA through the shikimate pathway, which is very active in *C. glutamicum* (a very potent producer of amino acids). A 13C metabolic flux analysis would have revealed this decoupling, as well as ascertained many other aspects of pathway flux distribution.

Finally, the authors have tried to produce PCA using aromatics from depolymerised lignin. But the titres have been more than 25-fold lower than what they achieved with synthetic aromatics. This result is similar to what many other workers have reported when using depolymerized lignin and continues to be the main hurdle in the quest for developing a meaningful lignin-valorization process having commercial potential. We are still very far from industrial-scale lignin valorization. Looking at the overall scientific quality and the results obtained using the approaches developed in the manuscript, I would like to recommend it for publication, subject to minor revisions and corrections pointed out in the specific comments below.

Response:

- ✓ We sincerely thank you for this comprehensive and insightful assessment of our work. We greatly appreciate the recognition of the scientific quality of the study and the positive evaluation of our metabolic engineering strategies.
- ✓ We fully acknowledge our point that the use of *Corynebacterium glutamicum* as a host for aromatic compound conversion has been previously reported. In this study, our focus is on the development of a layered, multi-input autonomous regulatory system combined with systematic pathway optimization, enabling efficient and near-complete conversion of multiple lignin-related aromatics. We believe this integrated strategy represents a meaningful advancement beyond prior studies.
- ✓ Regarding your comments on the potential contribution of glucose to product formation and the lack of detailed flux analysis, we agree that these are important considerations. In the present study, glucose was primarily supplied to support cell growth and energy requirements, while aromatic substrates served as source for products. To address this point more rigorously, we performed additional experiments. We have also clarified this aspect in the revised manuscript and discussed the potential contribution of central metabolism accordingly. We also acknowledge that more detailed analyses, such as ^{13}C metabolic flux analysis, would provide deeper insights and consider this an important direction for future work.

- ✓ Finally, we agree that the lower titers obtained from depolymerized lignin remain a significant challenge for the field. We have revised the Discussion section to better contextualize our results within current limitations and to highlight the broader implications and remaining challenges in lignin valorization.

Specific Comments:

1. Please provide a list of abbreviations in the main manuscript. Else, it is very difficult to keep track of the many abbreviations.

Response:

- ✓ Thank you for this helpful suggestion. A comprehensive list of abbreviations has now been added to the main manuscript to improve clarity and facilitate readability, given the number of abbreviations used throughout the text (located after the **Methods** section, Page 45, Line 972).

Abbreviations in the main manuscript

**Abbreviation**	**Full Term**
FA	Ferulic acid
p-CA	*p*-Coumaric acid
SA	Sinapic acid
CaA	Caffeic acid
PCA	Protocatechuic acid
4-HBA	*p*-Hydroxybenzoate
VA	Vanillic acid
CCM	*cis,cis-Muconic acid*
β-KA	β-Ketoadipic acid
aTFs	Transcription factors
BA	Benzoic Acid
12-DB	Catechol
13-DB	Resorcinol
14-DB	Hydroquinone
GA	Gentisic Acid
ALE	Adaptive laboratory evolution
EPS	Exopolysaccharide
SNV	Single-nucleotide variant
ITC	Isothermal titration calorimetry

2. Please provide sufficient literature references to the use of engineered *C. glutamicum*

for lignin valorization and for production of PCA and MA. For example, the article by Kugure et al., Metabolic Engineering 2018 has not been cited. They have used engineered *C. glutamicum* to produce PCA at nearly 83 g/L, much higher than what is claimed by this manuscript. Nor is there any discussion on the work done by Wittman's group using engineered C. glutamicum.

Response:

- ✓ We acknowledge that the omission of these references constrained the overall depth of our study, and we appreciate the opportunity to rectify this. We have now incorporated and discussed relevant literature on engineered *Corynebacterium glutamicum* for lignin valorization and aromatic compound production, including Kugure et al. and representative work from Wittmann's group.
- ✓ In the revised manuscript, we explicitly position our work within this context. We provide a comprehensive discussion of two related research areas: First, the current state of biosynthetic studies using engineered *Corynebacterium glutamicum* to produce PCA and related products, whether derived from lignin-related aromatics or other carbon sources such as glucose. Second, the current advances in lignin valorization for the production of high-value chemicals, with outstanding contributions from Wittmann's group. Based on this contextual discussion, we clarify that our study focuses on the development of a systematic optimization framework utilizing autonomous biological funneling for processing lignin-related aromatics. This represents a complementary strategy to existing approaches and addresses limitations in adaptability and regulatory flexibility. The literature mentioned has been cited in the manuscript, and the specific additional descriptions are as follows:

  "*Corynebacterium glutamicum* has emerged as a promising chassis for lignin valorization … and has been engineered to produce value-added chemicals from lignin-related substrates" (**Introduction**, Line82-85).

  "Currently, engineered *C. glutamicum* have demonstrated exceptional performance in the de novo synthesis of aromatic compounds. This microbial platform has achieved remarkable titers from glucose, including 82.7 g L^{-1} PCA and 88.2 g L^{-1} CCM. Notably, it produced 85 g L^{-1} CCM from catechol, the highest reported titer from lignin-related aromatics to date." (**Discussion**, Line 704-708).

3. Line 109-110: "…PCA and CCM producing strains achieved unprecedented titers of 53.40 g/L and 51.90 g/L respectively…". This (unprecedented titers) is not true, as

amply demonstrated by other literature reports. The titres reported in this manuscriptare good, but I request the authors not to overstate their case and report the better or competing values from literature

Response:

- ✓ Thank you for this valuable comment. We apologize that our previous phrasing was overly definitive and lacked the necessary scientific rigor. We fully appreciate your suggestion to report better or competing values from the literature. For example, Kogure et al. reported that engineered *Corynebacterium glutamicum* could produce up to 83 g L^{-1} of PCA from glucose. The highest titers of CCM have also been reported by the Wittmann group, who achieved up to 85 g L^{-1} CCM using catechol as the substrate. In our previous statement, we described our PCA and CCM titers of 53.4 g L^{-1} and 51.9 g/L as "representing the highest reported titers from lignin-related phenylpropanoids." We intended to restrict this claim specifically to the context of lignin-derived phenylpropanoid substrates.
- ✓ Nonetheless, we recognize your point that even when limiting the claim to lignin-related phenylpropanoid substrates, the previous wording could be misleading. We have therefore revised the manuscript to adopt a more precise and measured description, emphasizing the significance of our work, rather than overstating product titers. In the revised Introduction, we have replaced it with a more accurate description and now explicitly cite relevant literature reporting higher or comparable titers in Discussion. Specific revision are as follows:

  "… achieved unprecedented titers of …" has been revised to: "… achieved highly competitive titers …" (Line 109).

  "Currently, engineered *C. glutamicum* have demonstrated exceptional performance in the de novo synthesis of aromatic compounds. This microbial platform has achieved remarkable titers from glucose, including 82.7 g L^{-1} PCA and 88.2 g L^{-1} CCM. Notably, it produced 85 g L^{-1} CCM from catechol, the highest reported titer from lignin-related aromatics to date." (**Discussion**, Line704-708).

4. Line 109-110: "…achieveing a "normalized" conversion of over 99% to high-value chemicals, including PCA, CCM…". One cannot 'blindly' accept the 99% conversion. There is no evidence that there was no production of PCS from glucose through the shikimate pathway. There were no control experiments (using aromatics as the sole carbon source or growing the strains only on glucose) to prove otherwise.

Response:

- ✓ Thank you for this important comment. We fully agree that reporting "over 99% conversion" in the original manuscript without ruling out potential PCA production from glucose via native pathways was insufficiently rigorous. In direct response, we performed the key control experiment as suggested, culturing the production strain with glucose as the sole carbon source (without feeding any aromatics).
- ✓ The results clearly demonstrate that the intrinsic production of PCA from glucose in our engineered strain is negligible and fell below the reliable range of the calibration curve during 30h fermentation. And only less than 5 mg L^{-1} (0.032 mM) PCA was produced **after 48h**. This confirms that the high conversion rate we reported is indeed attributable to the efficient bioconversion of the supplied aromatic compounds, not background synthesis from glucose. Based on this additional data, we have revised the manuscript in **Results**, we now explicitly state that the reported conversion is a net value and reference the control experiment results. In addition, we have provided a thorough discussion in **Discussion** regarding the contribution of glucose to the synthesis of PCA and other products in this manuscript, as well as the interaction between glucose central carbon metabolism and phenylpropanoid metabolism, with relevant literature cited. Specific additional descriptions are as follows:

  "Additionally, control experiments using glucose as the sole carbon source showed negligible PCA production, confirming that the observed product formation originated from lignin-related aromatics rather than background synthesis from glucose (Supplementary Fig. 9a)." (Line 492-495).

  "Control experiments with glucose as the sole carbon source produced negligible CCM (Supplementary Fig. 9b), thereby confirming that the reported 99.98% conversion is attributable to the supplied phenylpropanoid substrates." (Line 568-571).

  "No β-KA was detected when glucose was used as the sole carbon source (Supplementary Fig. 9c), confirming complete dependence on phenylpropanoid substrates." (Line 587-589).

  "our experimental results confirm that the bioconversion of phenylpropanoids is effectively decoupled from glucose-supported cell growth. That aligns with recent ^{13}C-labeling studies which asserted that growth and phenylpropanoid-to-CCM conversion in engineered *C. glutamicum* operate through a coordinated division of labor" (Line 694-697).

  Due to space limitations, these experimental data have been included in

Supplementary Figure 9, and the method is described in **Materials and Methods**.

✓ We sincerely appreciate your suggestion, which has strengthened our work by adding this essential evidence and making our conclusions more robust. We sincerely appreciate your suggestion, which has strengthened our work by adding this essential evidence and making our conclusions more robust.

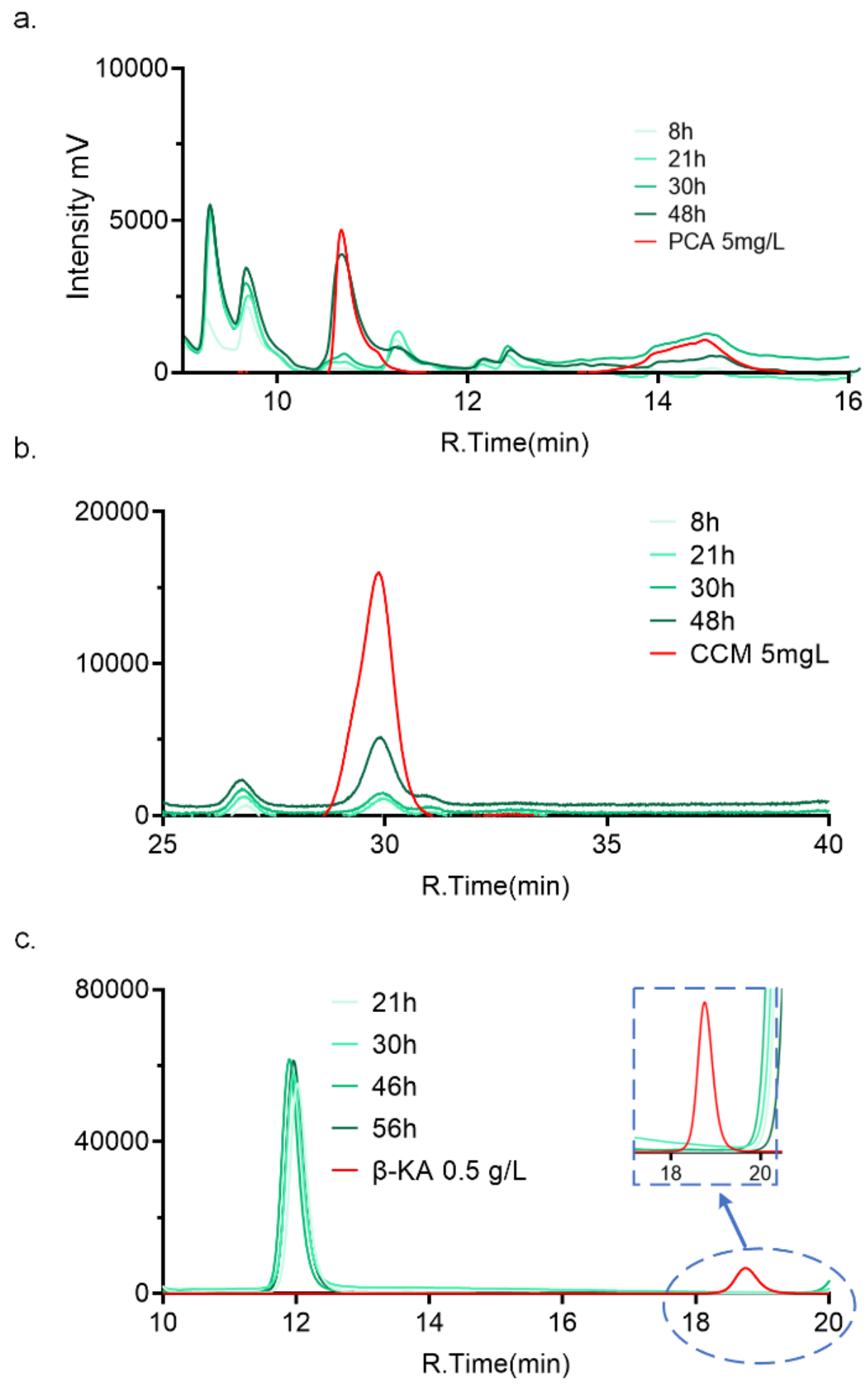

Supplementary Figure 9. HPLC chromatograms of fermentation samples using 10 g L^{-1} glucose as the sole carbon source.

(a) HPLC analysis of PCA in strain SASR7 fermented solely on glucose. The red line represents the 5 mg L^{-1} PCA standard, with a retention time of approximately 10.6 min.

(b) HPLC analysis of CCM in strain SASR7-C1 fermented solely on glucose. The red line represents the 5 mg L^{-1} CCM standard, with a retention time of approximately 30 min.

(c) HPLC analysis of β-KA in strain SASR7-K19 fermented solely on glucose. β-KA was derivatized to levulinic acid according to previously reported methods (see Materials and Methods). The red line represents the 5 mg L^{-1} levulinic acid standard, with a retention time of approximately 18.6 min.

5. Line 138-141: "…During fed-batch fermentation using three phenylpropanoids CaA,

p-CA and FA as substrates, strain Spca1 accumulated PCA at concentrations of 20.52 mM, 12.31 mM, and 7.79 mM, corresponding to molar yields of 38.49%, 29.38%, and 19.18% (mol/mol), respectively…" Was glucose used as a co-substrate in these fermentations? If not, why was it used later in the with experiments engineered highproducers?

Response:

- ✓ Thank you for raising this important point regarding the fermentation details. In all fermentations conducted in this study for the conversion of phenylpropanoids to target products, the engineered production strains—including Spca1 and its derivatives—have blocked pathways from phenylpropanoids to central carbon metabolism. For example, in Spca1, the *pcaHG* gene encoding protocatechuate-3,4-dioxygenase was deleted. Consequently, these strains cannot grow when phenylpropanoids are provided as the sole carbon source (the pathway from phenylpropanoids to central carbon metabolism is illustrated in the figure below). To support cell growth, glucose was therefore added as a co-substrate in all fermentations. Specifically, 10 g L^{-1} glucose was supplemented in all shake-flask fermentations (we modified the conventional CGXII medium by replacing the original 40 g L^{-1} glucose with 10 g L^{-1} glucose).

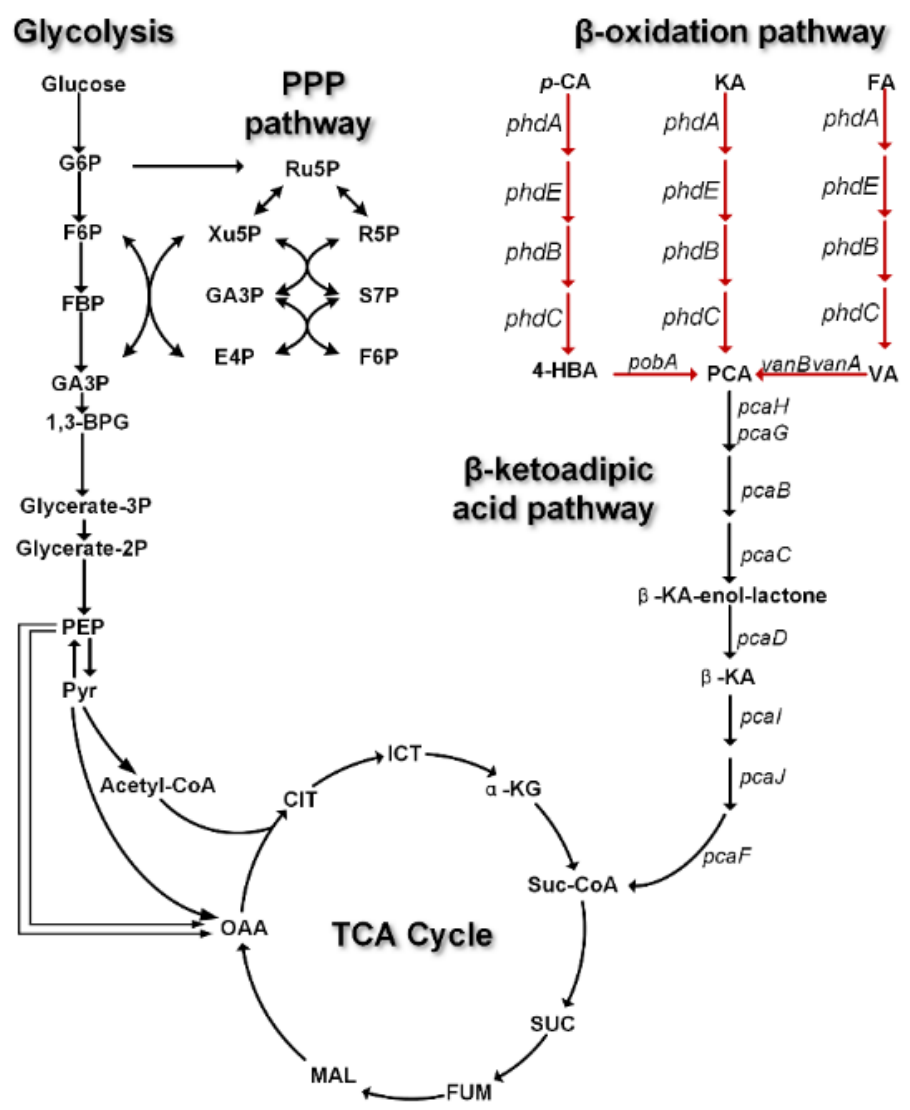

- ✓ We recognize that the original description in **Materials and Methods** may have been insufficiently clear, which could have caused confusion or misinterpretation. To improve readability and ensure accurate communication, we have carefully revised and supplemented the section "**Fermentation strategy using lignin-derived aromatics.**" (Line 768-774). We hope these modifications will facilitate

understanding and prevent any misinterpretation. We sincerely thank you for their careful reading and insightful comments, which have helped us enhance the clarity our manuscript.

6. Lines 134-135: "…To validate *C. glutamicum*'s native biological funneling of ligninderived phenylpropanoids…". Please replace the phrases 'lignin-derived phenylpropanoids' and 'lignin-derived aromatics' mentioned elsewhere in the manuscript with the phrase 'lignin-related aromatics' throughout the manuscript. The term 'lignin-derived aromatics' is misleading since the experiments have been conducted with synthetic aromatic compounds which are not derived from lignin but are similar to the ones which are derived from lignin.

Response:

- ✓ Thank you for this important clarification. We regret the use of misleading language in our earlier draft and are grateful for the chance to clarify our meaning more accurately. We fully agree that the terms "lignin-derived phenylpropanoids" and "lignin-derived aromatics" was misleading, as the experiments were conducted with synthetic aromatic compounds that are structurally similar to, but not directly derived from, lignin.
- ✓ In response, we have carefully replaced these terms throughout the manuscript with "lignin-related aromatics" to accurately reflect the nature of the substrates used. Corresponding changes have been made throughout the main text, including the **Abstract**, **Introduction**, **Results**, and **Discussion**.

7. Figure 3e: Are the strain symbols for Spe1 and SHT1 interchanged? One would expect SHT1 to exhibit higher substrate tolerance and give better titres? Please check and correct.

Response:

- ✓ Thank you for pointing this out. We are sorry for our previous fault in labelling strain symbols. Upon careful review, we realized that the strain symbols for Spca1 and SHT1 were indeed interchanged in Figure 3e. We have corrected the figure and have updated the figure legend accordingly. We apologize for any confusion caused and appreciate your careful reading.

8. Lines 423-424: "…we established a key design rule: increasing O2 operator copy

number progressively reduced dynamic range, providing a tunable output control strategy…" How does reducing the dynamic range provide a more tunable output? One would expect it to be the other way around?

Response:

- ✓ Thank you for this insightful and precise comment. We fully agree that the original statement was inappropriate. As a biosensor performance metric, a larger dynamic range is generally preferred. Our intention was to convey that increasing O2 operator copy number leads to different expression strength ranges—not a reduction in dynamic range—which can be useful when weaker expression is desired for a given ligand concentration. We apologize for the imprecise language and have revised the manuscript accordingly. The original sentence has been corrected to:
- ✓ "…Notably, increasing O2 operator copy number leads to decreased expression strength ranges, providing a tunable output control strategy…" (Line 424-426).

9. Lines 514-516: "…The initial total feed contained 49.38 mM lignin-derived phenylpropanoids (p-CA:FA:CaA=4:1:0.5) and approximately 10 g/L glucose." In the Materials and Methods section, it is mentioned as 14 g/L glucose. Why was glucose fed? For biomass generation? How does glucose consumption affect phenylpropanoid uptake? Are glucose utilization and phenylpropanoid utilization completely decoupled towards biomass and product formation? One sees a drastic increase in PCA yield and titres in these experiments – could these be due to theglucose being channelled to PCA through the shikimate pathway? Conversely, wouldn't the phenylpropanoids be also utilized for biomass formation? If this is the case, the bioconversion to product would not be close to 100%.

Response:

- ✓ Thank you for raising this critical point regarding glucose supplementation and its potential influence on phenylpropanoid bioconversion. First, we have rectified the statement 'approximately 10 g L^{-1}' to the precise concentration of 14 g L^{-1}. The discrepancy between 10 and 14 was due to differences between the initially estimated target concentration and the actual final concentration measured after addition, arising from inaccuracies in the preparation of the glucose stock solution. Subsequently, we will address each aspect of the comment in detail below. Given the constructive of these questions, we have also expanded **Discussion** to provide a thorough treatment of the interplay between glucose and phenylpropanoid

metabolism. We sincerely appreciate your insightful comments, which have helped to strengthen the manuscript.

Reason for glucose addition: 14 g L^{-1} glucose was added to support biomass generation. In all phenylpropanoid-to-product fermentations, the engineered strains (including SASR7, SASR7-C1, and SASR7-K19) have blocked pathways from phenylpropanoids to central carbon metabolism. For example, in SASR7 and SASR7-C1, the *pcaHG* gene encoding protocatechuate-3,4-dioxygenase was deleted (*pcaIJ* gene for SASR7-K19), rendering the strain unable to grow on phenylpropanoids as the sole carbon source. Therefore, glucose supplementation was necessary to sustain cell growth.

Interaction between glucose and phenylpropanoid utilization: Our experiments demonstrate that the contribution of glucose to the production of PCA, CCM, and β-KA is negligible (as shown in figure below). Regarding the effect of glucose on phenylpropanoid uptake, in wild-type *C. glutamicum*, transcription of the phenylpropanoid degradation pathway (phd gene cluster: *phdA, phdE, phdB, phdC*) is subject to carbon catabolite repression via the global regulator GlxR (analogous to CRP in *E. coli*). GlxR binds to promoter regions within the *phd* cluster, maintaining minimal transcription in the presence of glucose. Expression is only activated upon glucose depletion[4]. This regulatory behavior accounts for the low dynamic range observed in the wild-type PhdR-P_{phdB} biosensor.

In contrast, in our engineered strains (SASR1–9, and the CCM and β-KA production strains), the native *phd* promoters were replaced with synthetic constitutive promoters combined with operator elements. This design allows dynamic regulation of the genes while removing GlxR binding sites, rendering phenylpropanoid metabolism independent of glucose. Therefore, glucose inhibits phenylpropanoid metabolism in wild-type *C. glutamicum*, but in our engineered strains, phenylpropanoid metabolism is unaffected by glucose.

Decoupling of glucose and phenylpropanoid utilization: Experiments with glucose as the sole carbon source show that PCA production is minimal, accounting for only 0.049% of the total PCA detected in phenylpropanoid-fed fermentations. Moreover, this small amount of PCA appears after 48 h, whereas all phenylpropanoid-to-PCA fermentations in this study are completed within 30 h. In addition, the phenylpropanoid-to-central carbon metabolism pathway has been blocked in SASR7, meaning phenylpropanoids are theoretically not used for biomass formation. Together, these results indicate that glucose utilization and

phenylpropanoid utilization can be regarded as being decoupled in our system, with glucose supporting biomass and phenylpropanoids being channeled exclusively to product formation.

Potential contribution of glucose to PCA via the shikimate pathway: In principle, glucose can be converted to PCA via the shikimate pathway, and our experiments confirm that trace PCA can be detected from glucose. However, this occurs only after 48 h, and the amount is extremely low. Previous studies have shown that significant PCA production from glucose in *C. glutamicum* requires overexpression of the shikimate pathway and pathway from shikimate to PCA, along with substantial glucose supplementation, conditions that were not implemented in our experiments. Therefore, the contribution of glucose to PCA formation in our work in our study is negligible.

✓ "Notably, our experimental results confirm that the bioconversion of phenylpropanoids is effectively decoupled from glucose-supported cell growth. That aligns with recent ^{13}C-labeling studies which asserted that growth and phenylpropanoid-to-CCM conversion in engineered *C. glutamicum* operate through a coordinated division of labor." was added to **Discussion** (Line 693-697).

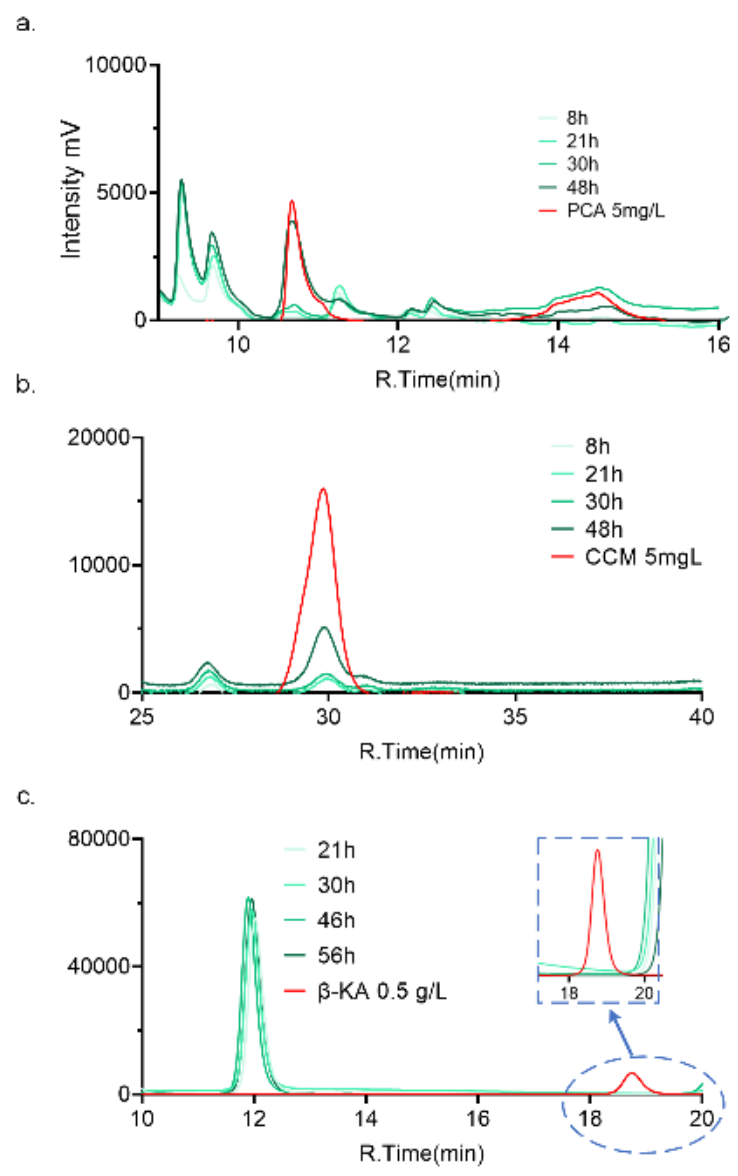

Supplementary Figure 9. HPLC chromatograms of fermentation samples using 10 g L^{-1} glucose as the sole carbon source.

(a) HPLC analysis of PCA in strain SASR7 fermented solely on glucose. The red line represents the 5 mg L^{-1} PCA standard, with a retention time of approximately 10.6 min.

(b) HPLC analysis of CCM in strain SASR7-C1 fermented solely on glucose. The red line represents the 5 mg L^{-1} CCM standard, with a retention time of approximately 30 min.

(c) HPLC analysis of β-KA in strain SASR7-K19 fermented solely on glucose. β-KA was derivatized to levulinic acid according to previously reported methods (see Materials and Methods). The red line represents the 5 mg L^{-1} levulinic acid standard, with a retention time of approximately 18.6 min.

10. Lines 516-517: "Phenylpropanoid consumption was monitored in real-time to guide intermittent feeding …" How was this monitored in real-time? Online sensor or offline analysis? If online, what sensor was used? If offline, what were the time intervals for measurement and how did that affect the intermittent feeding?

Response:

- ✓ Thank you for this important question and for seeking clarification regarding the monitoring strategy. In this study, phenylpropanoid consumption was not monitored using an online sensor, but rather through manual sampling followed by offline HPLC analysis. Samples were taken at regular intervals (typically every 3-6 h, specify if possible), and substrate concentrations were quantified to assess consumption trends. Based on these measurements, intermittent feeding was manually adjusted to maintain substrate availability within an appropriate range. We acknowledge that the use of the term "real-time" in the original manuscript may have been misleading. We have revised the text to more accurately describe this process as:

  "Substrate consumption was monitored to guide intermittent feeding, maintaining a 1:1 molar ratio between phenylpropanoids and glucose." (Line 549-550).

  We have also added the feeding strategy in the revised **Materials and Methods** (Line 780-781) to improve transparency and reproducibility.

  We appreciate your comment, which has helped us improve the precision of our description.

11. Lines 568-571: "This process achieved a PCA titer of 12.38 mM from the depolymerized lignin stream… corresponding to a molar yield of 99.44% from corn stover derived phenylpropanoids…". This PCA production is 30-fold lower than with synthetic substrates. This gives an idea of the limitations (inhibitory effects) of working with real lignin. Was glucose used as co-substrate in this experiment? If not, why not?

Response:

- ✓ Thank you for this insightful comment regarding PCA production from corn stover derived phenylpropanoids This is a point well-taken, as it addresses a critical

challenge: the significant discrepancy in product yields when using real alkaline-pretreated agricultural waste compared to purified commercial substrates. We will faithfully acknowledge and analyze this limitation in **Discussion** and provide an outlook for our future work.

- ✓ First, in these experiments, 10 g L^{-1} glucose was added as an energy source to support strain growth. And PCA production was all from corn stover derived phenylpropanoids.

  Second, the PCA titer (~12.38 mM) from the depolymerized lignin stream was substantially lower than that observed with synthetic phenylpropanoid substrates. This difference reflects the fact that phenylpropanoids are released in limited quantities from the pendant groups linking lignin and hemicellulose. These phenylpropanoids constitute only a minor fraction of the lignocellulosic complex, typically representing 3% – 4% of the total biomass by weight. However, their biological significance is amplified by their susceptibility to release via alkaline depolymerization. Unlike the recalcitrant core lignin polymer, these pendant groups are readily dissociable, thereby providing an accessible substrate flux for engineered microbial platforms.
- ✓ We have clarified this point in the **Discussion** of revised manuscript to highlight the challenges associated with real lignin streams. Specific additional descriptions are as follows:

  "Although phenylpropanoids constitute only a minor fraction of the lignocellulosic complex, typically 3%–4% by weight, their biological significance is strongly amplified by their susceptibility to release via alkaline depolymerization. Unlike the recalcitrant core lignin polymer, these pendant groups are readily dissociable, thereby providing an accessible substrate flux for engineered microbial platforms. However, the industrial lignin valorization necessitates a heightened level of metabolic plasticity. A strategic imperative for achieving this goal involves further expanding the substrate repertoire of the autonomous biological funneling in *C. glutamicum* …." (Line 725-733).

12. All fermentation-related experiments (batch and fed-batch) with lignin-related aromatics must be described together in the Material and Methods section

Response:

- ✓ Thank you for this valuable suggestion to improve the clarity and reproducibility of our manuscript. We fully agree that consolidating all fermentation details in the

Materials and Methods section is essential.

- ✓ In the revised manuscript, we have revised the "**Fermentation strategy using lignin-related aromatics**" subsection within the **Materials and Methods** to provide a comprehensive and unified description (Line 775-782). The updated subsection now includes detail information of all fermentation-related experiments.

Reviewer #4:

I co-reviewed this manuscript with one of the reviewers who provided the listed reports. This is part of the Nature Communications initiative to facilitate training in peer review and to provide appropriate recognition for Early Career Researchers who co-review manuscripts.

References

1. Werner, A.Z. et al. Lignin conversion to β-ketoadipic acid by Pseudomonas putida via metabolic engineering and bioprocess development. *Science Advances* **9**, eadj0053.
2. Martim, D.B. et al. Resolving the metabolism of monolignols and other lignin-related aromatic compounds in Xanthomonas citri. *Nature Communications* **15**, 7994 (2024).
3. Liu, H. et al. Engineering Pseudomonas putida for lignin bioconversion into cis-cis muconic acid. *Chemical Engineering Journal* **495**, 153375 (2024).
4. Weiland, F. et al. Metabolic engineering of Corynebacterium glutamicum for increased cis, cis-muconate production from plant-derived p-hydroxycinnamates via deregulated pathway flux and increased CoA intermediate availability. *Metabolic Engineering* **92**, 262-283 (2025).
5. Weiland, F., Kohlstedt, M. & Wittmann, C. Guiding stars to the field of dreams: Metabolically engineered pathways and microbial platforms for a sustainable lignin-based industry. *Metabolic Engineering* **71**, 13-41 (2022).
6. Kallscheuer, N. et al. Identification of the phd gene cluster responsible for phenylpropanoid utilization in Corynebacterium glutamicum. *Applied Microbiology and Biotechnology* **100**, 1871-1881 (2016).
7. Morabbi Heravi, K., Lange, J., Watzlawick, H., Kalinowski, J. & Altenbuchner, J. Transcriptional Regulation of the Vanillate Utilization Genes (vanABK Operon) of Corynebacterium glutamicum by VanR, a PadR-Like Repressor. *Journal of Bacteriology* **197**, 959-972 (2015).

Reviewer #2 (Remarks to the Author):

Xu el al revision

The authors have made extensive revision to the originally submitted manuscript and significantly tone, rhetoric and overhyped terminology. They have additionally performed further analysis to i) validate the strain under heterogeneous aromatic compositions, ii) benchmark against a non-inducible strain, and iii) provide ITC data consistent with the literature.

Minor:

Figure one, lower right panel, still includes reference “Master system” terminology which has now been removed from elsewhere in the manuscript.

- ✓ Thank you for your careful reading and review, we will revise the lower right panel of Figure 1 to remove the 'Master system' terminology for consistency with the rest of the manuscript.

ROUND 1 REVIEWER 3 ATTACHMENT:

General Comments:

Microbial bioconversion of lignin is an important part of the overall effort to valorize lignocellulosic biomass, as evident from the surge of recent literature reports on this topic. However, this is not an easy task due to the recalcitrant nature of lignin as well as the toxic nature of the aromatics obtained from depolymerized lignin. Most of the metabolic engineering efforts to valorize lignin have predominantly focused on *Pseudomonas putida* as the host strain, although there also quite a few reports on the use of engineered *Corynebacterium glutamicum* for this purpose, notably from Christopher Wittman's group (e.g. see Becker et al. MCF 2018; Weiland et al. Met Eng. 2023 etc.). So, the authors of this manuscript are not doing something novel by employing *C. glutamicum* as the host strain. However, the authors have demonstrated certain unique approaches to rational metabolic engineering of *C. glutamicum* and managed to engineer strains which can consume all three major lignin-related aromatics (caffeate, p-coumarate and ferulate) and demonstrate production of reasonably high titres of protocatechuate (PCA) and muconic acid (MA) at near 100% conversion.

To begin with, they successfully evolved the native strains by adaptive laboratory evolution (ALE), in order to tolerate high concentrations of the toxic substrates. After identifying critical genetic changes in the genome sequence of the evolved strains, they have reverse-engineered the wild-type strains to take up higher concentrations of the substrates and efficiently channelled them towards the desired metabolites. Furthermore, they identified and engineered the allosteric transcription factors (aTFs) which bind to the substrates, as well as the cognate promoter systems, which has enabled the development tunable expression systems (aromatic response regulators) for controlling the metabolic fluxes. Using this strategy, they have efficiently de-bottlenecked the pathways to avoid accumulation of inhibitory intermediates and channelled all the substrates towards accumulation of the key intermediate, viz. PCA. PCA can be produced as the main product by knocking out the downstream genes as well as channelled towards the production of MA by overexpressing the *aroY* gene.

Using the engineered strains, the authors developed fed-batch processes to obtain high titres of the products in lab-scale bioreactors. These kind of titres have been reported in literature using other strains as well as by using sugars as substrates along with lignin. In their study, the authors have used glucose as a co-substrate along with lignin-related aromatics. However, it is not very clear if the utilization of glucose (presumably only for biomass production) is completely de-coupled from the utilization of aromatics (which are presumably used only for product formation). Glucose can be potentially channelled toward formation of PCA through the shikimate pathway, which is very active in *C. glutamicum* (a very potent producer of amino acids). A 13C metabolic flux analysis would have revealed this decoupling, as well as ascertained many other aspects of pathway flux distribution.

Finally, the authors have tried to produce PCA using aromatics from depolymerised lignin. But the titres have been more than 25-fold lower than what they achieved with synthetic aromatics. This result is similar to what many other workers have reported when using depolymerized lignin and continues to be the main hurdle in the quest for developing a meaningful lignin-valorization process having commercial potential. We are still very far from industrial-scale lignin valorization. Looking at the overall scientific quality and the results obtained using the approaches developed in the manuscript, I would like to recommend it for publication, subject to minor revisions and corrections pointed out in the specific comments below.

Specific Comments:

1. Please provide a list of abbreviations in the main manuscript. Else, it is very difficult to keep track of the many abbreviations.
2. Please provide sufficient literature references to the use of engineered *C. glutamicum* for lignin valorization and for production of PCA and MA. For example, the article by Kugure et al., *Metabolic Engineering* 2018 has not been cited. They have used engineered *C. glutamicum* to produce PCA at nearly 83 g/L, much higher than what is claimed by this manuscript. Nor is there any discussion on the work done by Wittman's group using engineered *C. glutamicum*.
3. Line 109-110: "…*PCA and CCM producing strains achieved unprecedented titers of 53.40 g/L and 51.90 g/L respectively*…". This (unprecedented titers) is not true, as amply demonstrated by other literature reports. The titres reported in this manuscript

are good, but I request the authors not to overstate their case and report the better or competing values from literature

4. Line 109-110: "…*achieveing a "normalized" conversion of over 99% to high-value chemicals, including PCA, CCM*…". One cannot 'blindly' accept the 99% conversion. There is no evidence that there was no production of PCS from glucose through the shikimate pathway. There were no control experiments (using aromatics as the sole carbon source or growing the strains only on glucose) to prove otherwise.
5. Line 138-141: "…*During fed-batch fermentation using three phenylpropanoids CaA, p-CA and FA as substrates, strain Spca1 accumulated PCA at concentrations of 20.52 mM, 12.31 mM, and 7.79 mM, corresponding to molar yields of 38.49%, 29.38%, and 19.18% (mol/mol), respectively*…" Was glucose used as a co-substrate in these fermentations? If not, why was it used later in the with experiments engineered high-producers?
6. Lines 134-135: "…*To validate C. glutamicum's native biological funneling of lignin-derived phenylpropanoids*…". Please replace the phrases 'lignin-derived phenylpropanoids' and 'lignin-derived aromatics' mentioned elsewhere in the manuscript with the phrase 'lignin-related aromatics' throughout the manuscript. The term 'lignin-derived aromatics' is misleading since the experiments have been conducted with synthetic aromatic compounds which are not derived from lignin but are similar to the ones which are derived from lignin.
7. Figure 3e: Are the strain symbols for Spe1 and SHT1 interchanged? One would expect SHT1 to exhibit higher substrate tolerance and give better titres? Please check and correct.
8. Lines 423-424: "…*we established a key design rule: increasing O2 operator copy number progressively reduced dynamic range, providing a tunable output control strategy*…" How does reducing the dynamic range provide a more tunable output? One would expect it to be the other way around?
9. Lines 514-516: "…*The initial total feed contained 49.38 mM lignin-derived phenylpropanoids (p-CA:FA:CaA=4:1:0.5) and approximately 10 g/L glucose*." In the Materials and Methods section, it is mentioned as 14 g/L glucose. Why was glucose fed? For biomass generation? How does glucose consumption affect phenylpropanoid uptake? Are glucose utilization and phenylpropanoid utilization completely decoupled towards biomass and product formation? One sees a drastic increase in PCA yield and titres in these experiments – could these be due to the

glucose being channelled to PCA through the shikimate pathway? Conversely, wouldn't the phenylpropanoids be also utilized for biomass formation? If this is the case, the bioconversion to product would not be close to 100%.

10. Lines 516-517: "*Phenylpropanoid consumption was monitored in real-time to guide intermittent feeding*,…" How was this monitored in real-time? Online sensor or off-line analysis? If online, what sensor was used? If offline, what were the time intervals for measurement and how did that affect the intermittent feeding?
11. Lines 568-571: "*This process achieved a PCA titer of 12.38 mM from the depolymerized lignin stream… corresponding to a molar yield of 99.44% from corn stover derived phenylpropanoids*…". This PCA production is 30-fold lower than with synthetic substrates. This gives an idea of the limitations (inhibitory effects) of working with real lignin. Was glucose used as co-substrate in this experiment? If not, why not?
12. All fermentation-related experiments (batch and fed-batch) with lignin-related aromatics must be described together in the Material and Methods section
