# Supplementary material for: Engineering a robust and autonomous biological funneling for efficient valorization of lignin-related aromatics
Source: Nat Commun. 2026 Jun 17;17:7659. doi: 10.1038/s41467-026-74424-y (PMC13434420; doi:10.1038/s41467-026-74424-y)
Supplement: Supplementary file 3 [file 41467_2026_74424_MOESM1_ESM.pdf]

Engineering a robust and autonomous biological funneling for efficient valorization of lignin-related aromatics

Xu *et al.*

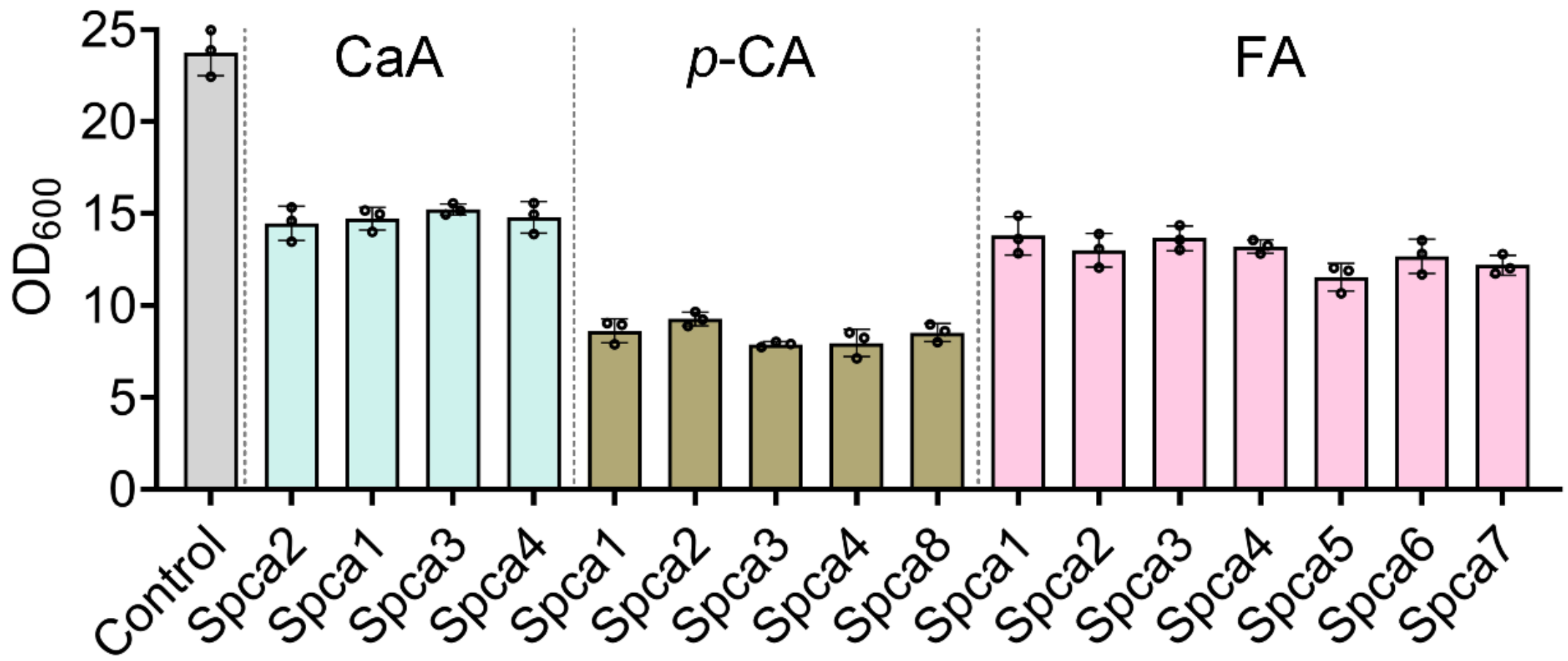

Supplementary Figure 1. Final OD_{600} values for wild type *C. glutamicum* in defined CGXII media with Spca1-Spca8 cultured in in defined CGXII media supplied with CaA, *p*-CA, and FA, respectively. Total substrate inputs supplied in five sequential pulses over 56 h for respective strains: CaA was supplied in strains Spca1–Spca4, with total inputs of 45.45, 46.35, 47.68 and 47.22 mM, respectively. (light green bars). Total *p*-CA inputs were 48.65, 48.22, 48.00, 47.98 and 48.27 mM for strains Spca1–Spca4 and Spca8, respectively (dark brown bars). Total FA inputs were 43.48, 43.20, 43.35, 43.72, 43.30, 43.72 and 42.82 mM for strains Spca1–Spca7, respectively, (pink bars). Abbreviations: FA, ferulic acid; *p*-CA, *p*-coumaric acid; CaA, caffeic acid. Values are presented as mean ± s.d. (n = 3 independent biological replicates). Source data are provided as a Source Data file.

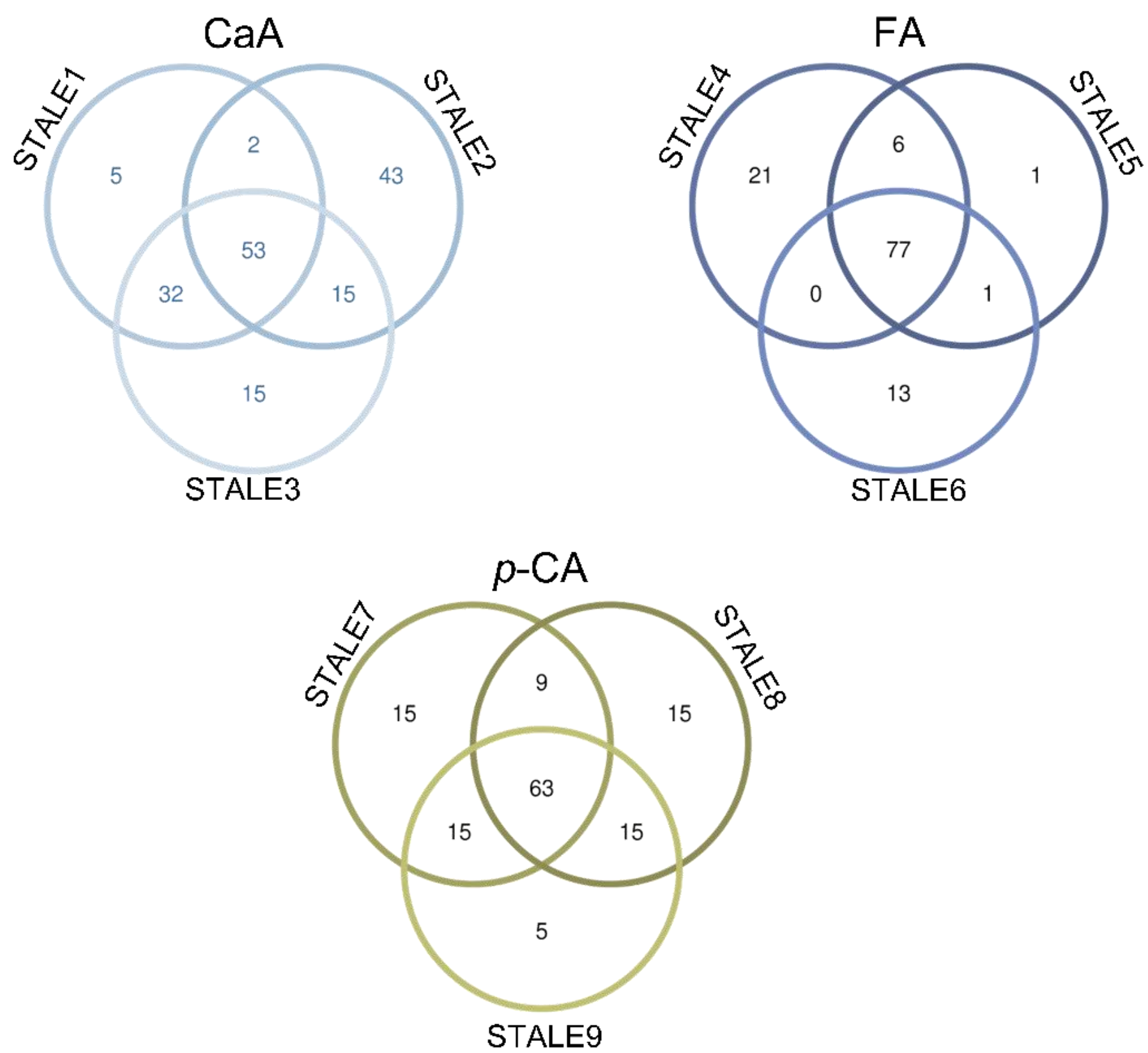

Supplementary Figure 2. Mutation profiles following ALE of CaA, FA, and *p*-CA. Venn diagrams of mutation sites (SNVs and InDels) in STALE1-STALE3, STALE4-STALE6, and STALE7-STALE9 strains. Abbreviations: FA, ferulic acid; *p*-CA, *p*-coumaric acid; CaA, caffeic acid.

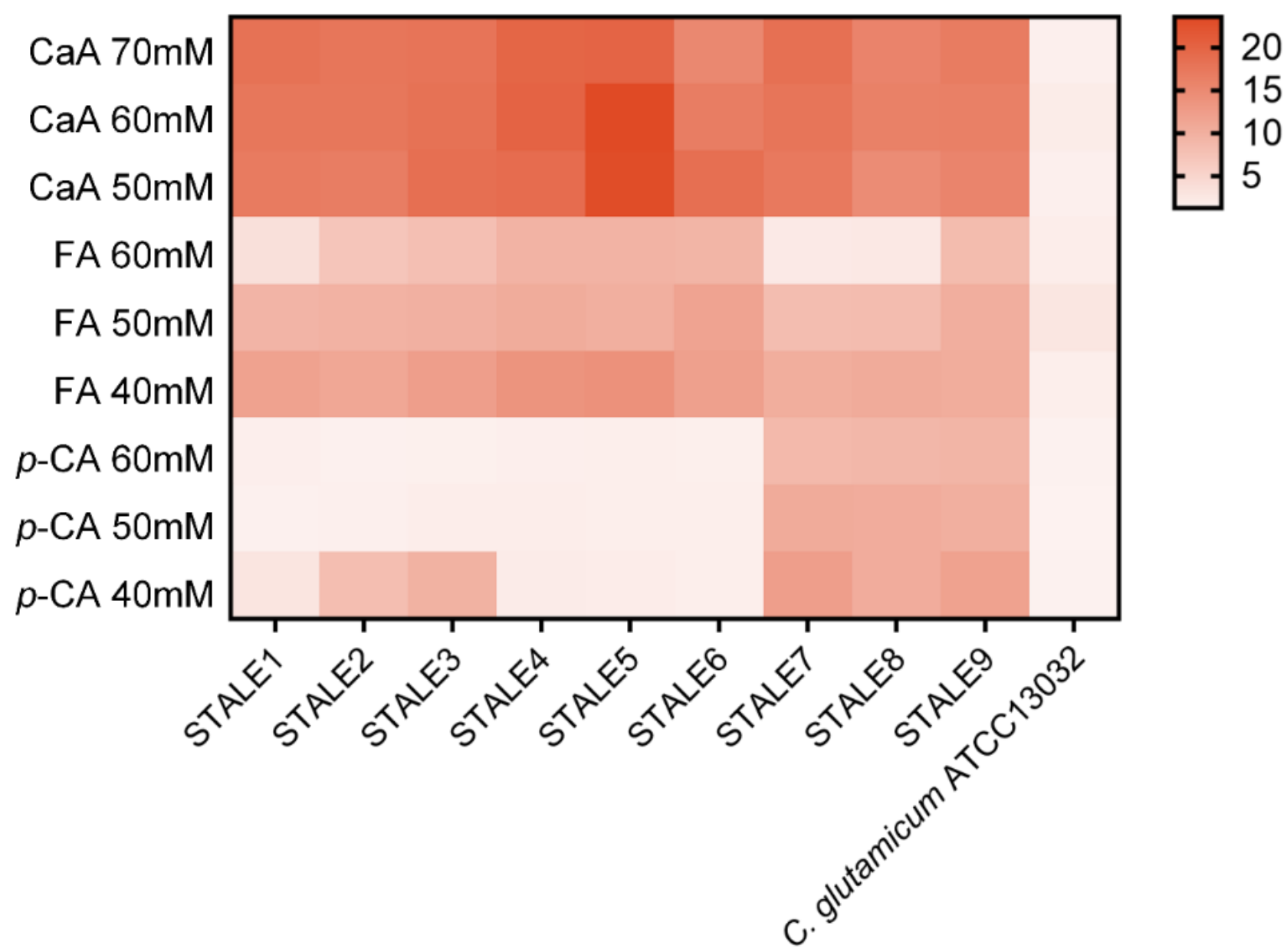

Supplementary Figure 3. Cross-tolerance to all three substrates in STALE1-STALE9. Abbreviations: FA, ferulic acid; *p*-CA, *p*-coumaric acid; CaA, caffeic acid. Values are presented as mean ± s.d. (n = 3 independent biological replicates). Source data are provided as a Source Data file.

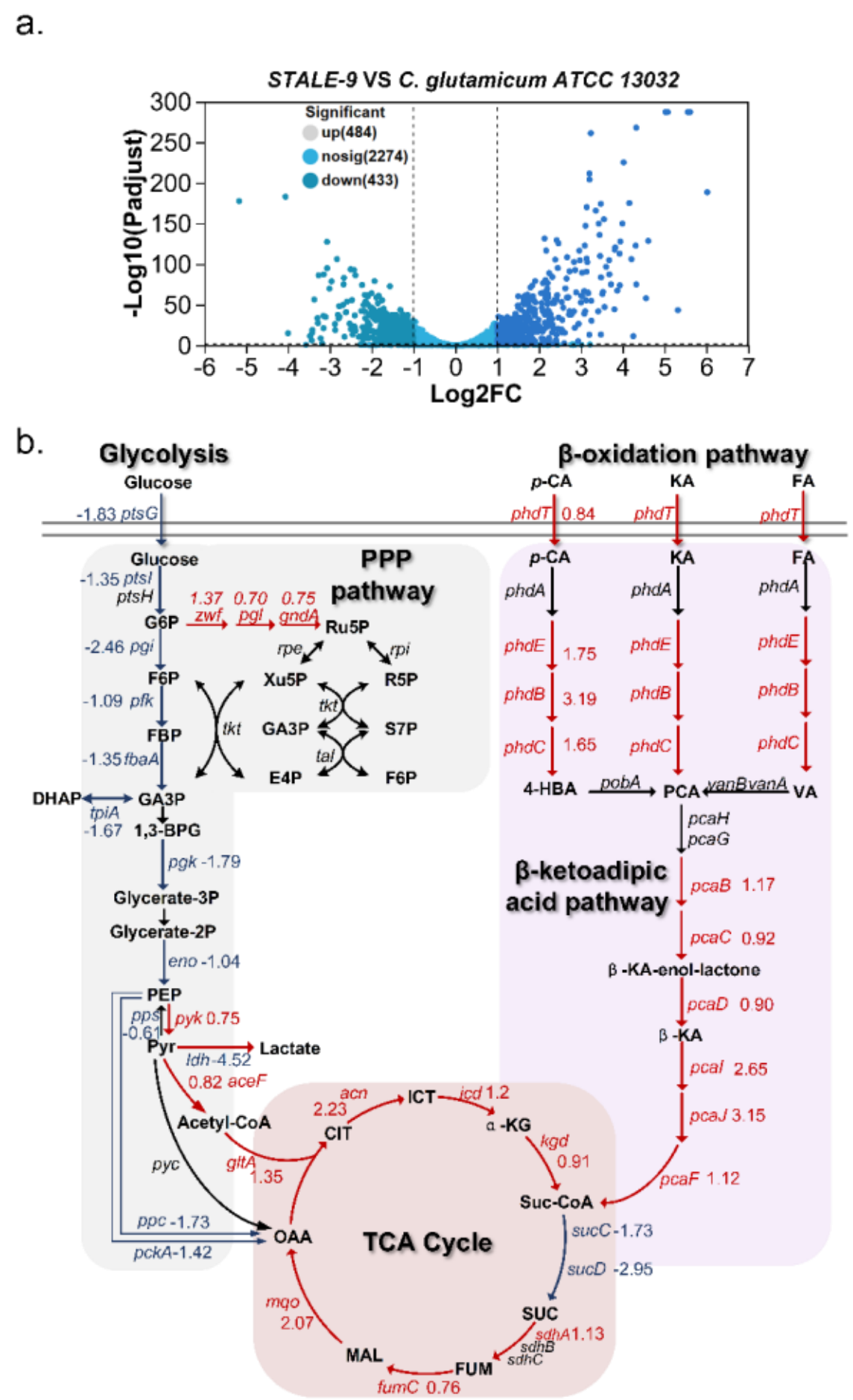

Supplementary Figure 4. Transcriptome analysis of the evolved strain STALE9 compared to the wild type *C. glutamicum* under *p*-CA stress. a. Transcriptome analysis of the evolved strain STALE9 with supplementation of 16mM *p*-CA revealed 917 differentially expressed genes compared to the wild type, with 484 up-regulated genes and 433 down-regulated genes. **b.** Transcriptional changes in central carbon metabolism and phenylpropanoid metabolic pathway genes of wild-type *C. glutamicum* with supplementation of 16mM *p*-CA. G6P: Glucose-6-phosphate; F6P: Fructose-6-phosphate; GA3P: Glyceraldehyde-3-phosphate; PEP: Phosphoenolpyruvate; OAA: Oxaloacetate; Acetyl CoA: Acetoacetyl coenzyme A; CIT: Citrate; ICT: Isocitrate; αKG: Alpha-ketoglutarate; Suc-CoA: Succinyl coenzyme A; SUC: Succinic acid; FUM: Fumarate; Mal: Malate; Xu5P: Xylulose-5-phosphate; R5P: Ribose-5-phosphate; TCA: Tricarboxylic acid cycle; β-KA-enol-lactone: β-ketoadipic enol lactone; β-KA: β-ketoadipic acid; *ptsG* encodes glucose transporters; *ptsI* encodes phosphoenolpyruvate-protein phosphotransferase; *pgi* encodes phosphoglucose isomerase; *pfk* encodes phosphofructokinase 1; *fbaA* encodes class II fructose-bisphosphate aldolase; *pgk* encodes phosphoglycerate kinase; *eno* encodes phosphopyruvate hydratase; *pyk* encodes pyruvate kinase; *gltA* encodes citrate synthase; *pckA*

encodes phosphoenolpyruvate carboxykinase; *acn* encodes aconitate hydratase; *icd* encodes NADP-dependent isocitrate dehydrogenase; *kgd* encodes α-ketoglutarate dehydrogenase; *sucC* encodes succinate-CoA ligase subunit beta; *sucD* encodes succinate-CoA ligase subunit alpha; *fumC* encodes class II fumarate hydratase; *mqo* encodes malate dehydrogenase; *phdT* encodes MHS family transporter; *phdA* encodes acyl-CoA ligase; *phdE* encodes enoyl-CoA hydratase; *phdB* encodes 3-hydroxyacyl-CoA dehydrogenase; *phdC* encodes 3-oxoacyl-CoA ketohydrolase; *pobA* encodes 4-hydroxybenzoate-3-hydroxylase; *vanAB* encodes vanillate O-demethylase; *pcaHG* encodes protocatechuate-3,4-dioxygenase; *pcaB* encodes β-carboxy-*cis,cis*-muconate lactonizing enzyme; *pcaC* encodes γ-carboxymuconolactone decarboxylase; *pcaD* encodes β-ketoadipate enol-lactone hydrolase; *pcaIJ* encodes β-ketoadipate succinyl-CoA transferase; *pcaF* encodes β-ketoadipyl-CoA thiolase.

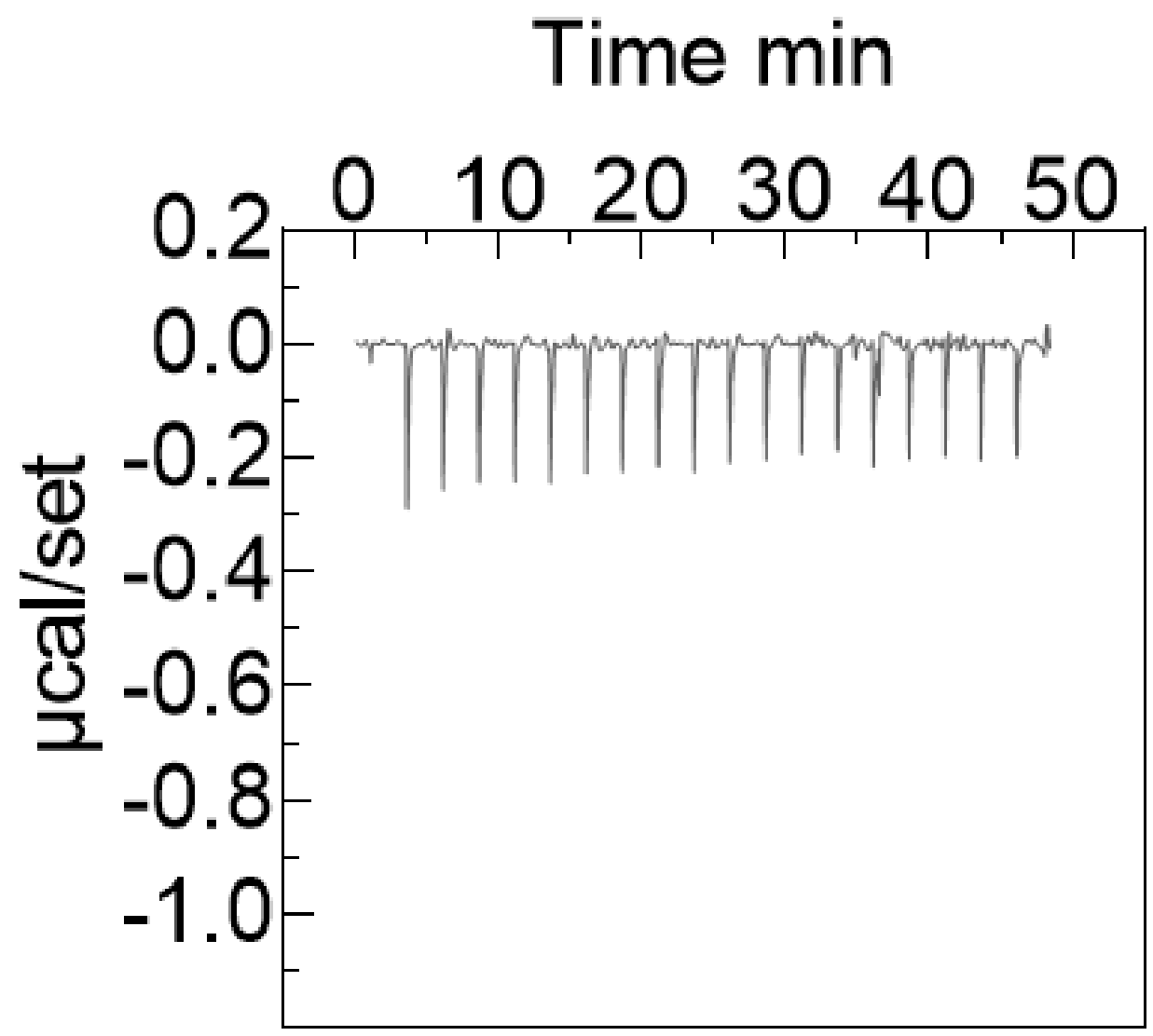

Supplementary Figure 5. Control experiment of isothermal titration calorimetry of PhdR with ligands, in which protein desalting buffer was titrated with PhdR. Source data are provided as a Source Data file.

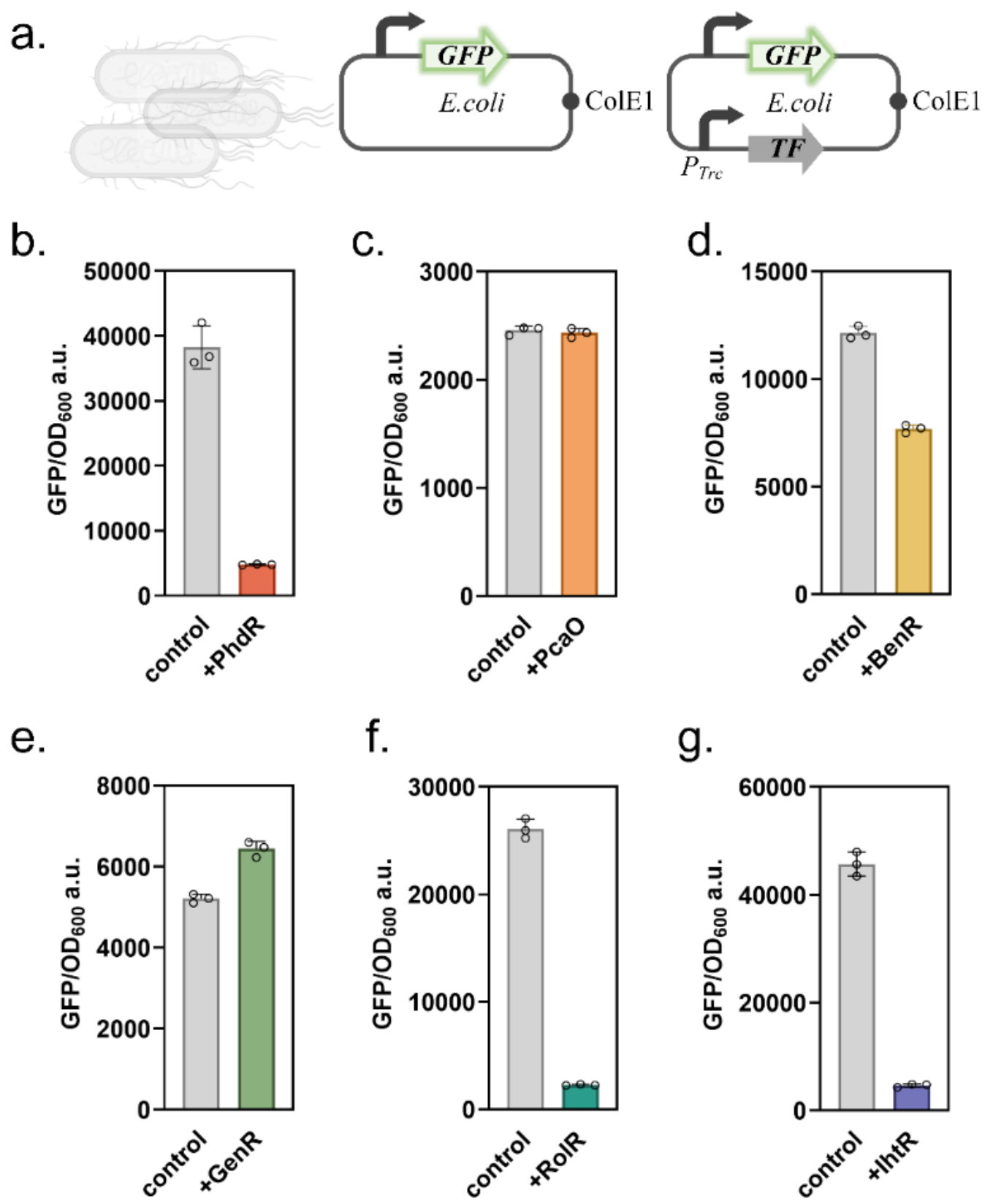

Supplementary Figure 6. Functional validation of aTFs. a. Schematic representation of plasmids with and without aTF expression in *E. coli.* Comparison of fluorescence intensity with and without phdR **(b)**, PcaO **(c)**, BenR **(d)**, GenR **(e)**, RolR **(f)**, and IhtR **(g)** expression in *E. coli*. Values are presented as mean ± s.d. (n = 3 independent biological replicates). Source data are provided as a Source Data file.

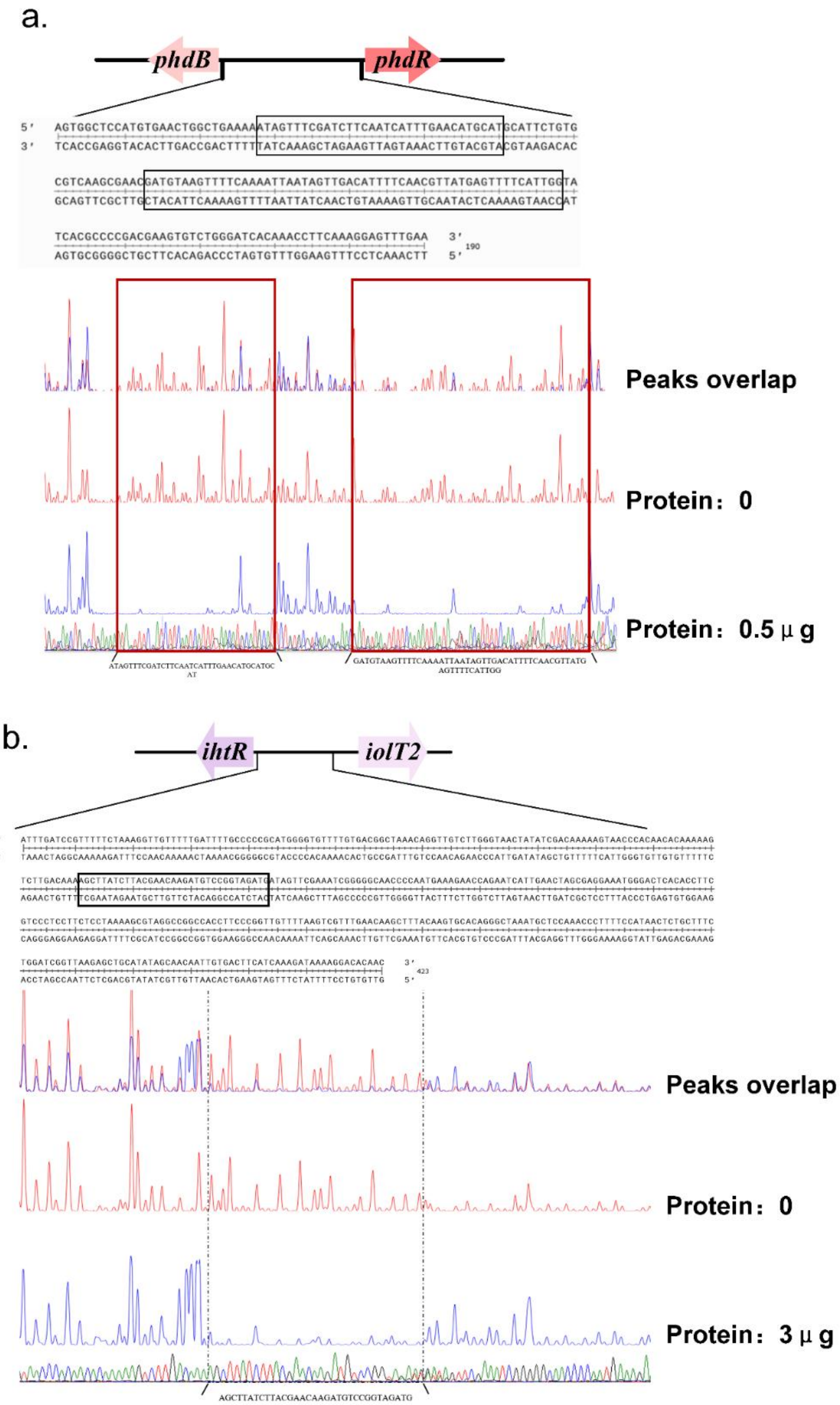

Supplementary Figure 7. Identification of PhdR and IhtR binding sites on promoters.
DNase I footprinting of PhdR **(a)**, and IhtR **(b)**.

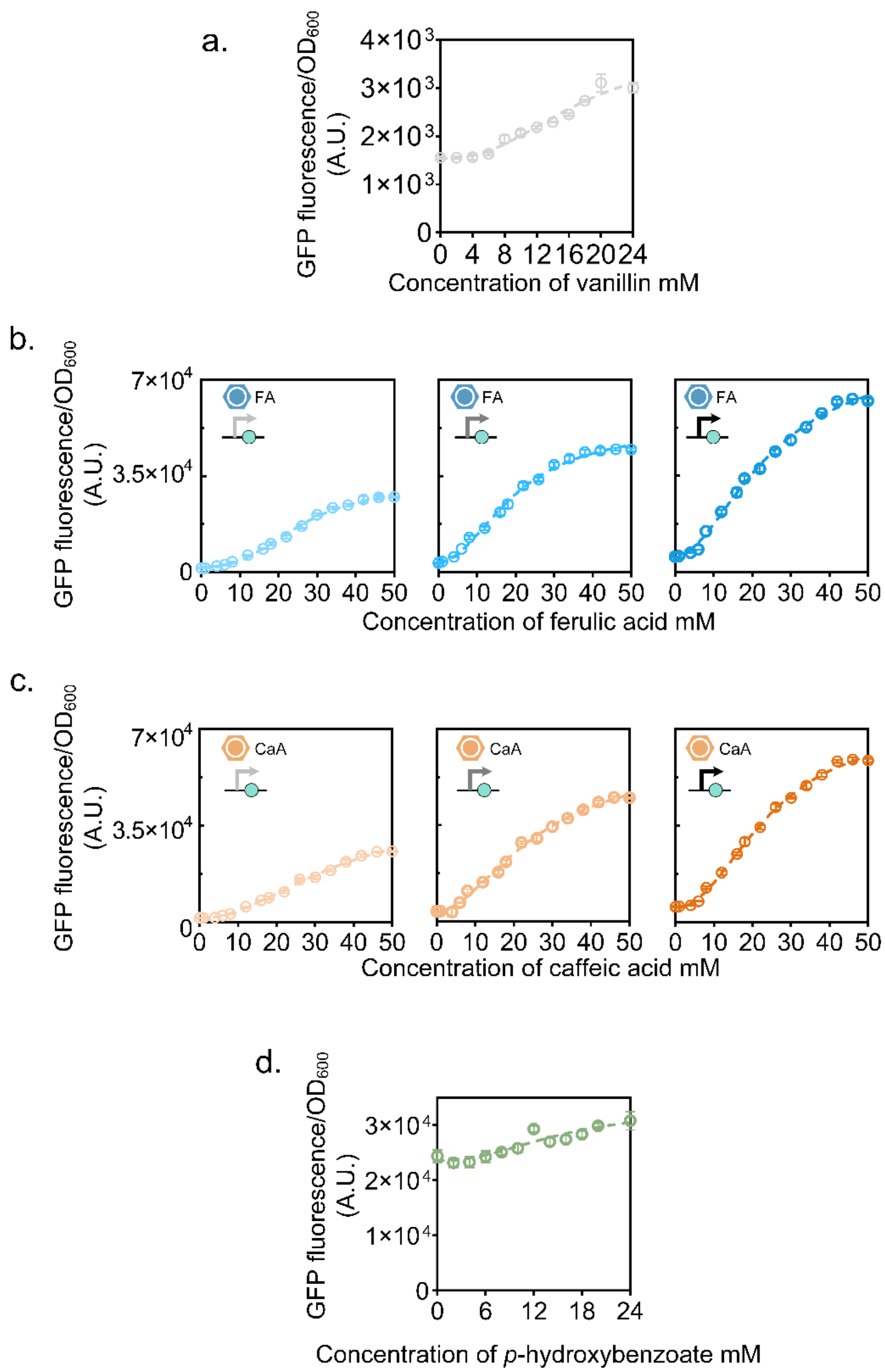

Supplementary Figure 8. Dose-response profiles of native biosensors and the optimized hybrid promoters. a. Initial dose-response curve of the VanR-VA-P_{vanA}. Dose-response curves of the optimal three *phd* hybrid promoters ($P_{Bba\text{-}23110}$-O2, $P_{Bba\text{-}23106}$-O2, $P_{Bba\text{-}23119}$-O2) supplemented with FA **(b)** and CaA **(c)**. **d.** Dose-response Curve of engineered hybrid promoters of PcaO-4-HBA-P_{pobA}. Abbreviations: FA, ferulic acid; CaA, caffeic acid. Values are presented as mean ± s.d. (n = 3 independent biological replicates). Source data are provided as a Source Data file.

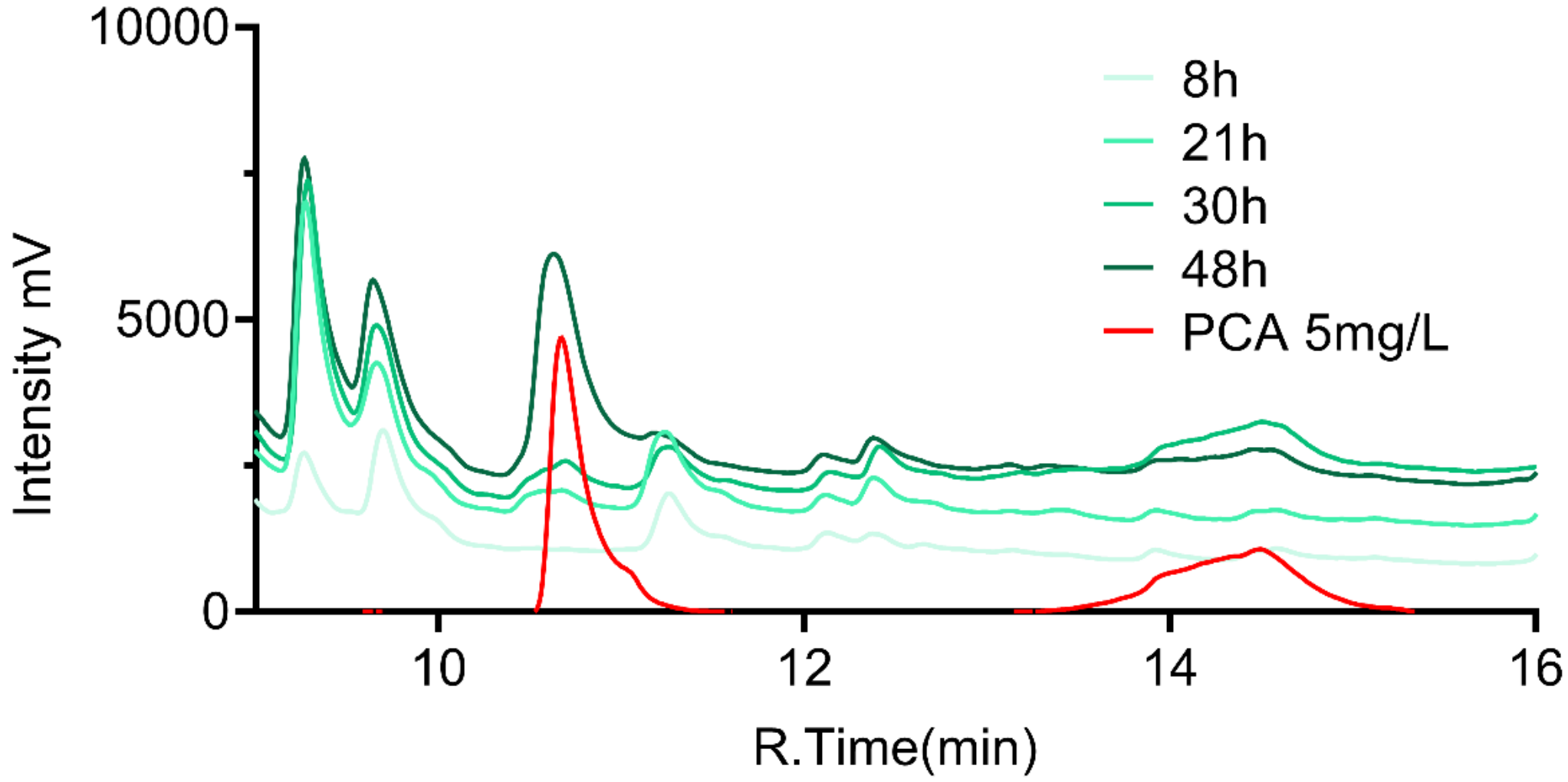

Supplementary Fig. 9. HPLC analysis of protocatechuic acid (PCA) produced by strain SASR7 grown on 10 g/L glucose as the sole carbon source. The red line represents the 5 mg/L PCA standard, with a retention time of approximately 10.6 min. Source data are provided as a Source Data file.

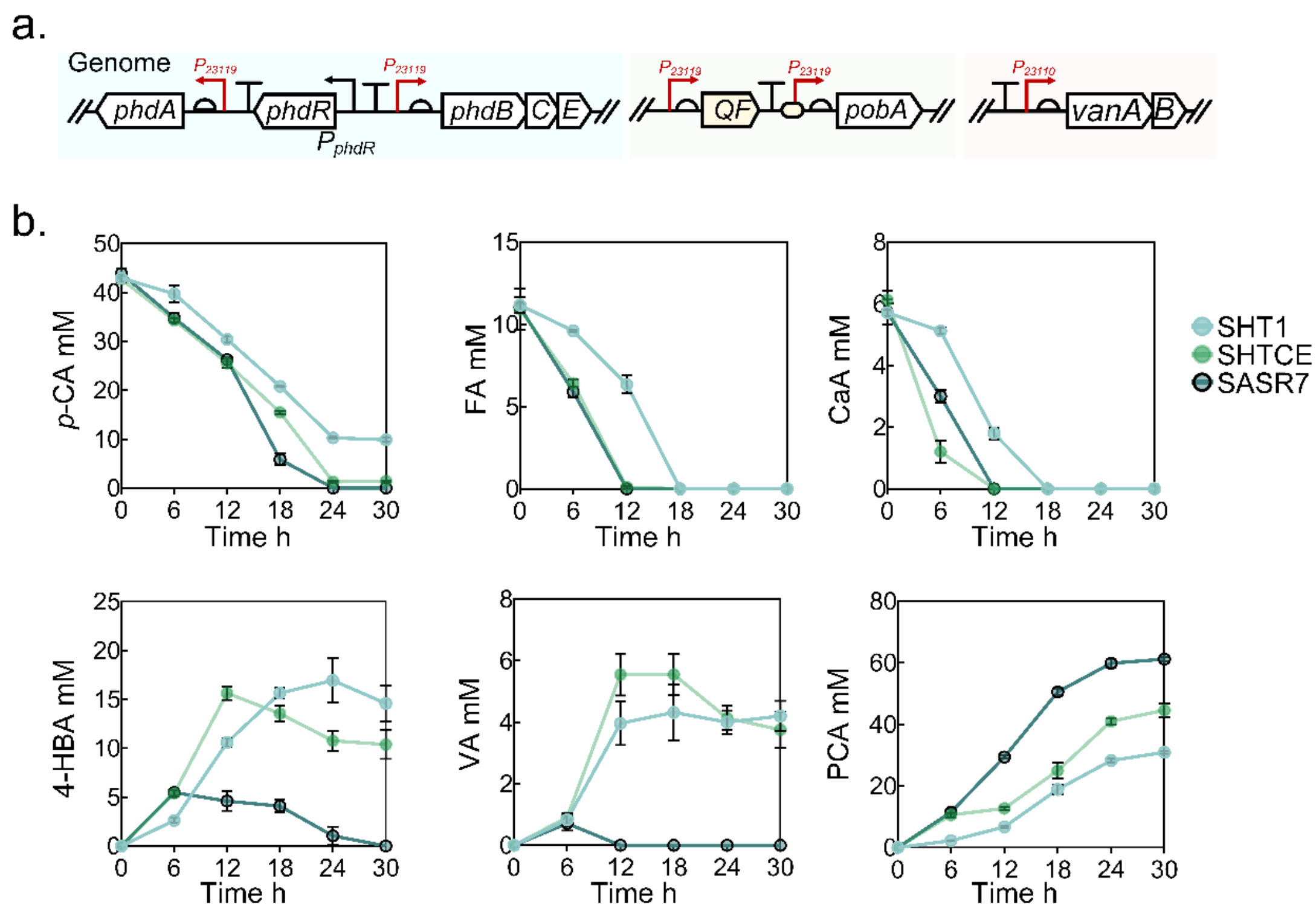

Supplementary Fig. 10. Construction of SHTCE and comparative fermentation performance with SHT1 and SASR7. a. Schematic illustration of the genome integration strategy for SHTCE. Native promoters of the target gene clusters were replaced with constitutive promoters of defined strengths to enable constitutive expression of all biological funneling genes (*phdAEBC*, *vanAB*, and *pobA*). The specific promoters used are indicated in red in the schematic. Regulatory elements, gene organization, and integration loci are shown as depicted. **b.** Time-course fermentation profiles of strains SHT1, SHTCE, and SASR7 cultivated with a mixed phenylpropanoid substrate containing of *p*-CA, FA, and CaA at a molar ratio of 4:1:0.5. Residual substrate concentrations (p-CA, FA, and CaA), intermediate metabolites 4-HBA and VA, and the final product PCA are shown over time. Abbreviations: FA, ferulic acid; *p*-CA, *p*-coumaric acid; CaA, caffeic acid; PCA, protocatechuic acid; 4-HBA, *p*-hydroxybenzoate; VA, vanillic acid. Values are presented as mean ± s.d. (n = 3 independent biological replicates). Source data are provided as a Source Data file.

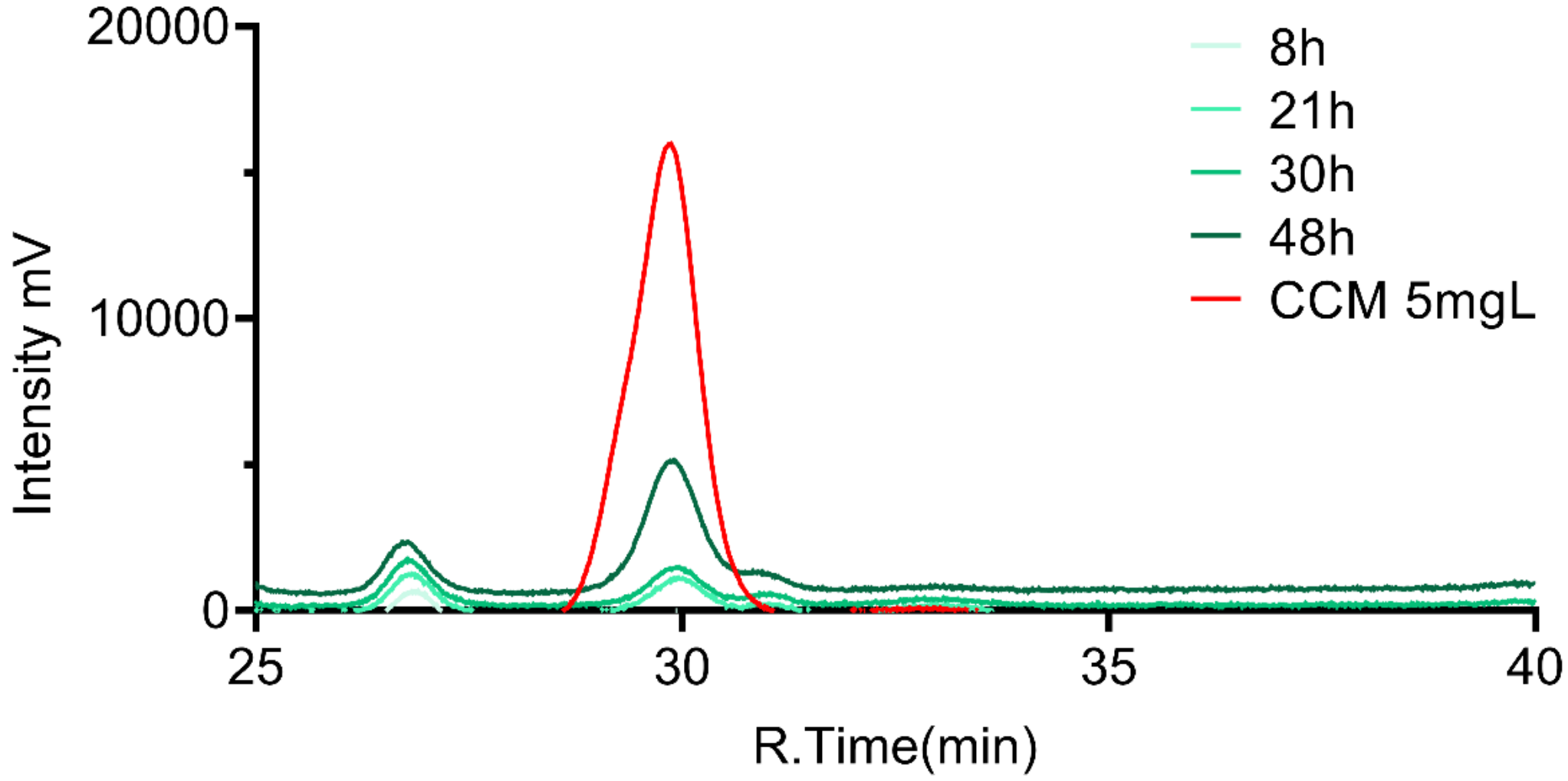

Supplementary Fig. 11. HPLC analysis of *cis,cis*-muconic acid (CCM) produced by strain SASR7-C1 grown on 10 g/L glucose as the sole carbon source. The red line represents the 5 mg/L CCM standard, with a retention time of approximately 30 min. Source data are provided as a Source Data file.

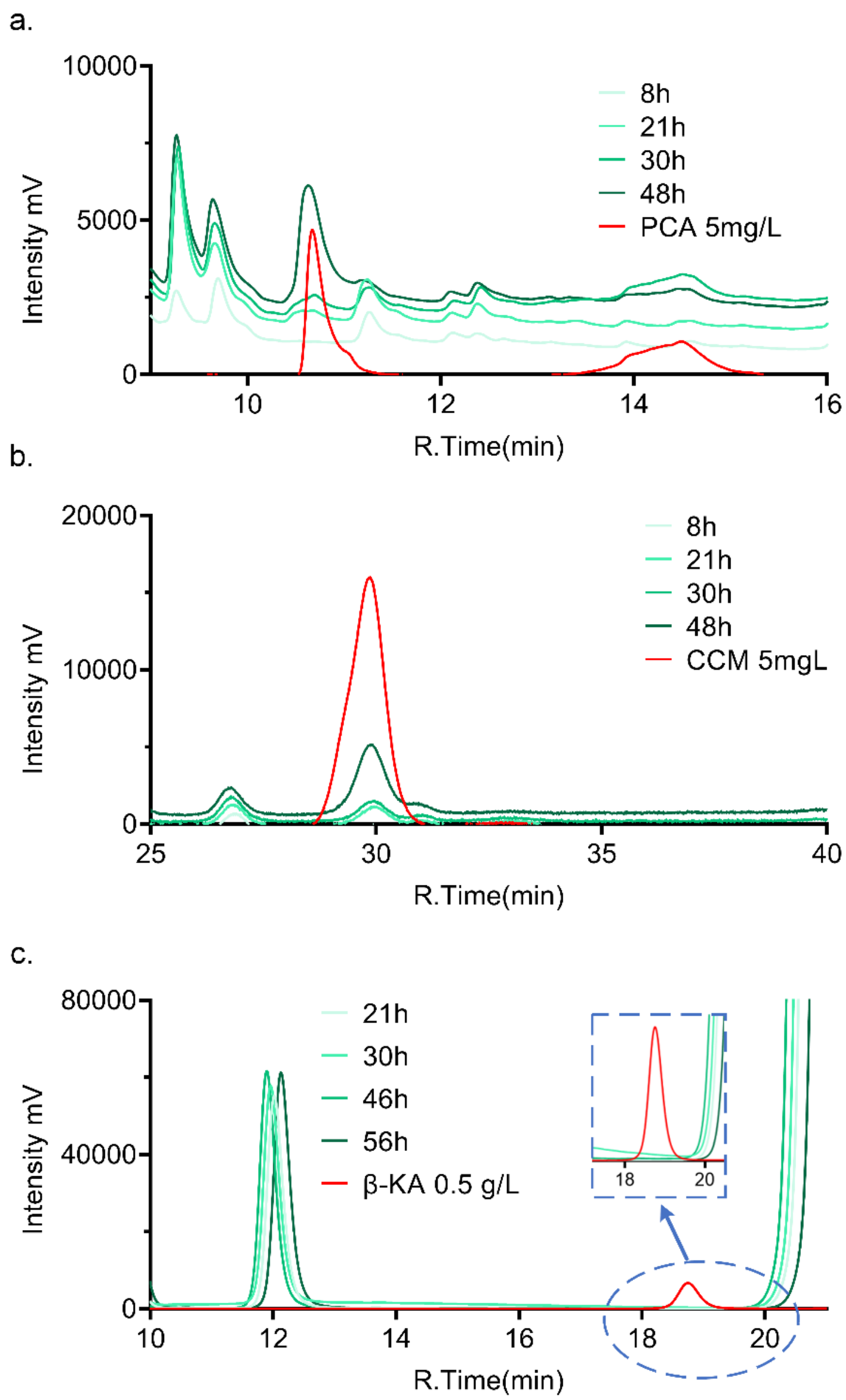

Supplementary Fig. 12. HPLC analysis of β-Ketoadipic acid (β-KA) produced by strain SASR7-K19 grown on 10 g/L glucose as the sole carbon source. β-KA was derivatized to levulinic acid according to previously reported methods (see Methods). The red line represents the 5 mg/L levulinic acid standard, with a retention time of approximately 18.6 min.

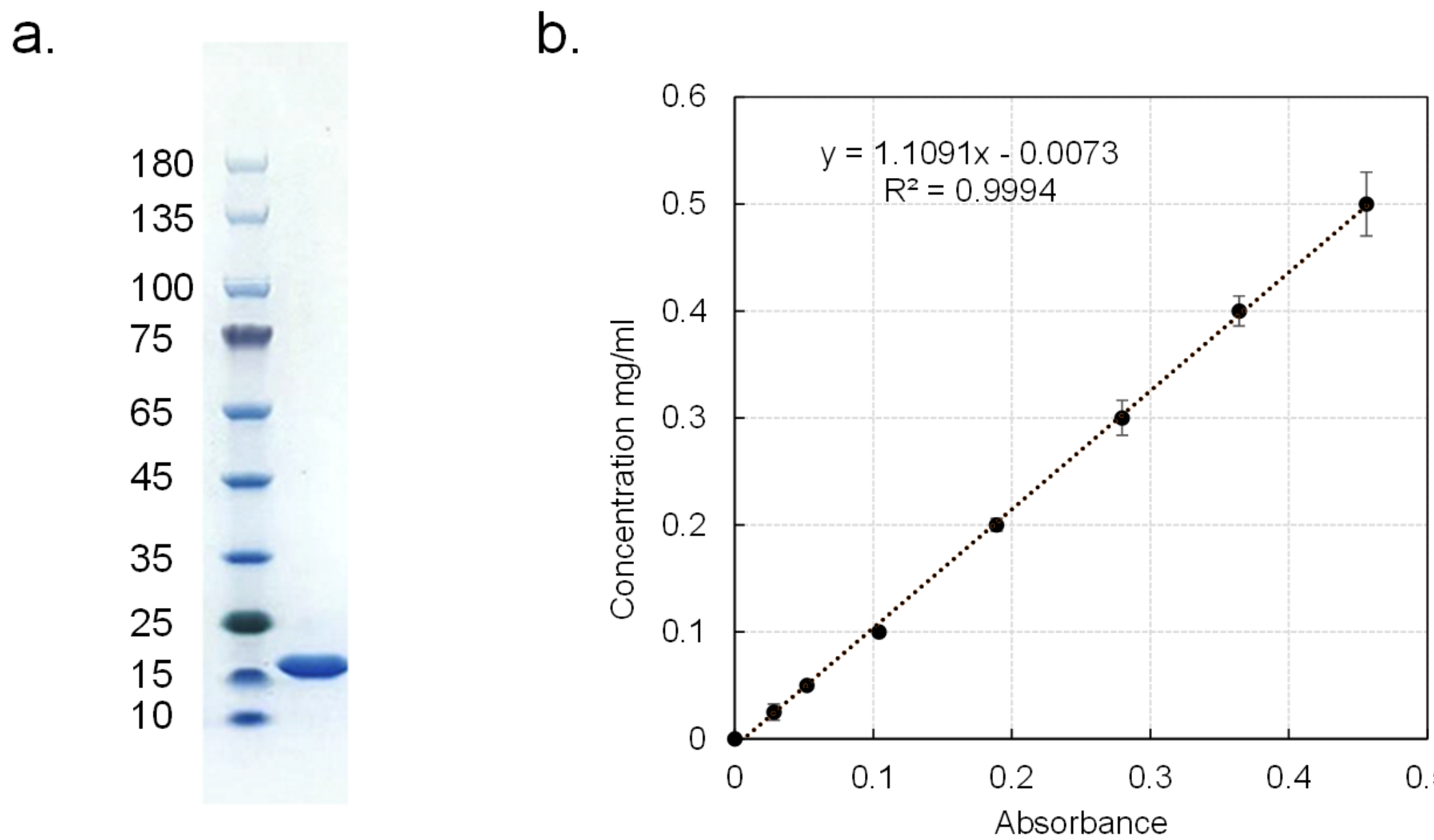

Supplementary Fig. 13. Verification and quantification of PhdR protein purification. a. SDS-PAGE analysis of PhdR after multi-step purification; **b.** Standard curve of the BCA protein quantification kit, with the absorbance at 562 nm on the x-axis and the protein concentration on the y-axis. Values are presented as mean ± s.d. (n = 3 independent biological replicates).

Supplementary Table 1. Sequences of heterologous enzyme.

Gene	Suorce	Amino acid sequence
praI^{Paj}	*Paenibacillus sp. JJ-1b*	MRTQVGIIGAGPAGLLLSHLLYLQGIESIIIENRTREEIEGTIRAGVLEQGTVDLMNQMGVGARMMKEGHFHEGFELRFNGRGHRINVHELTGGKYVTVYAQHEVIKDLVAARLQTGGQIHFNVGDVSLHDVDTSSPKIRFRPNKDGELQEIECDFIAGCDGFRGPSRPAIPQSVRKEYQKVYPFSWLGILVEAPPSAHELIYANHERGFALVSTRSPQIQRLYLQVDAQDHIDNWSDDRIWSELHARLETRDGFKLLEGPIFQKGIVSMRSFVCDPMQHGRLFLAGDAAHIVPPTGAKGLNLAAADVQVLARGLEAYYKAGKMEILNRCTEICLRRIWKAERFSWFMTTMLHRDQGHTPFERGIQLAELDYVTSSRAASTSLAENYIGLPMEF*
pobA^{Ppu}	*Pseudomonas putida KT2440*	MKTQVAIIGAGPSGLLLGQLLHKAGIDNIIVERQTAEYVLGRIRAGVLEQGTVDLLREAGVAERMDREGLVHEGVELLVGGRRQRLDLKALTGGKTVMVYGQTEVTRDLMQAREASGAPIIYSAANVQPHELKGEKPYLTFEKDGRVQRIDCDYIAGCDGFHGISRQSIPEGVLKQYERVYPFGWLGLLSDTPPVNHELIYAHHERGFALCSQRSQTRSRYYLQVPLQDRVEEWSDERFWDELKARLPAEVAADLVTGPALEKSIAPLRSLVVEPMQYGHLFLVGDAAHIVPPTGAKGLNLASDVNYLYRILVKVYHEGRVDLLAQYSPLALRRVWKGERFSWFMTQLLHDFGSHKDAWDQKMQEADREYFLTSPAGLVNIAENYVGLPFEEVA*
vanAB^{Ppu}	*Pseudomonas putida KT2440*	*vanA:*<br>MYPKNTWYVACTPDEIATKPLGRQICGEKIVFYRARENQVAAVEDFCPHRGAPLSLGYVEDGNLVCGYHGLVMGCDGKTVSMPGQRVRGFPCNKTFAAVERYGFIWVWPGDQAQADPALIPHLEWAVSDEWAYGGGLFHIGCDYRLMIDNLMDLTHETYVHASSIGQKEIDEAPPVTTVTGDEVVTARHMENIMAPPFWRMALRGNGLADDVPVDRWQICRFTPPSHVLIEVGVAHAGKGGYHAEAQHKASSIVVDFITPESDTSIWYFWGMARNFAAHDQTLTDNIREGQGKIFSEDLEMLERQQQNLLAHPERNLLKLNIDAGGVQSRKVLERIIAQERAPQPQLIATSANPA*
		vanB:<br>MIDAVVVSRNDEAQGICSFELAAADGSLLPAFSAGAHIDVHLPDGLVRQYSLCNHPEERHRYLIGVLNDPASRGGSRSLHEQVQAGARLRISAPRNLFPLAEGAQRSLLFAGGIGITPILCMAEQLSDSGQAFELHYCARSSERAAFVERIRSAPFADRLFVHFDEQPETALDIAQVLGNPQDDVHLYVCGPGGFMQHVLDSAKGLGWQEANLHREYFAAAPVDASNDGSFAVQVGSTGQVFEVPADRTVVQVLEENGIEIAMSCEQGICGTCLTRVLQGTPDHRDLFLTEEEQALNDQFTPCCSRSKTPLLVLDI*
vanAB^{Acb}	*Acinetobacter sp ADP1*	*vanA:*<br>MDVIIHKIHQLTPSIRAFELISANGSDLPAFDAGSHIDVHLKNGLTRQYSLSNCCSEQHRYVIGVLHDENSRGGSRCIHQDYREGDHLNIGTPRNLFEIHSKTQKAVLFAGGIGITPILSMAYRLKHQQIPFELHYFVRSHEMIAFYGNLTEHFPEQIHFHIQNQSETQCEMSKVLEEVAPDRHLYVCGPAGFMQFVMDSAQQAGWSDEQLHQEHFVAPQIDQSQNEAFTIEVLGSDRKIEVSAHQTATQALLEHGFDVPVSCEQGICGTCITRVVSGTPDHRDVFMTDEEHALNDQFTPCCSRAKSKILVIDLA*
		vanB:<br>MFIKNAWYVACRPEEIQDKPLGRTICGEKIVFYRGKENKVAAVEDFCPHRGAPLSLGYVEDGHLVCGYHGLVMGCEGKTIAMPAQRVGGFPCNKAYAVVEKYGFIWVWPGEKSLANEADLPTLEWADHPEWSYGGGLFHIQCDYRLMIDNLMDLTHETYVHSSSIGQKEIDEALPVTKVDGDHVVTERYMENIIAPPFWQMALRGNHLADDVPVDRWQRCHFFAPSNVHIEVGVAHAGHGGYNAPKDKKVASVVVDFITPETETSHWYFWGMARNFQPENQQLTDQIREGQGKIFTEDLEMLEQQQQNILRNPQRQLLMLNIDAGGVQSRKIIDRLLAKENSPSPQDTQRKFPNIRVI*
vanAB^{Cef}	*Corynebacterium efficiens*	*vanA:*<br>MCGGDTRKFYPLFYCRSAHAGLNCDSQHQLSMSLDSSLDSHSSRLTFPQKAEAPMTTTSAASSTASSTTTAKILPHPLNAWYVAAWDHEVTSKGIISRTIANKPLALYRTQDGRAVALADACWHRLAPLSKGKLTGRDGIQCPYHGLVYNSAGRCMSMPAQETLNPSAAVASFPVVEQYRYIWVWLGDPTLADPTTVPDMHQMTHPEWTGDGKTIAAACNYQLVLDNLMDLTHEEFVHSSSIGQDELSEADFEVTHTEDSVTVSRWMRDIDPPPFWQKNMNDKFPGYEGKVDRWQIIHYYYPSTICIDVGVAKAGTGAPEGDRSQGVNGYVMNTITPETDRSSHYFWSFQRNYRLDSQLITTQLRDGVHGVFGEDEEMLTAQQEAIDANPDYEFYNLNIDAGGMWVRRILEKALEAENRLHIPTTSAPVRKES*
		vanB:<br>MGSPHPGKGPGGGEPPAHPHHIRTGQEGVLMDTGSATNLLWQNAEVIATTEIADRIRRITLRPEHPHKVRPGEHLKVKVDINGQQTERSYSIVDADPEGAEVSLTVFHTPNSRGGSTFMHSLVPGQRITVTGPQQDFPLRIGAPRYVLVAGGIGITAIRGMAGLLRRLGADYTIHYAARSPEAMAYREELIAEHGDRLHLYLDSESVTLDIPALIDTIDSGTELYMCGPIRLMDAIRRAWRERELDPTNLRFETFGNSGWFLPESFRVTIPRLGVSTEISENESILEALEKIGVEMMSDCRRGECGLCQVKVLDREGQIDHRDVFFSDRQKKASEKLCACVSRVASTSAAPVPGNSLTELITPDNDSTAPGGRNIHLTIDVP*
aroY	*Enterobacter cloacae* ATCC 13047	MQNPINDLRSAIALLQRHPGHYIETDHPVDPNAELAGVYRHIGAGGTVKRPTRTGPAMMFNSVKGYPGSRILVGMHASRERAALLLGCVPSKLAQHVGQAVKNPVAPVVVPASQAPCQEQVFYADDPDFDLRKLLPAPTNTPIDAGPFFCLGLVLASDPEDTSLTDVTIHRLCVQERDELSMFLAAGRHIEVFRKKAEAAGKPLPVTINMGLDPAIYIGACFEAPTTPFGYNELGVAGALRQQPVELVQGVAVKEKAIARAEIIIEGELLPGVRVREDQHTNTGHAMPEFPGYCGEANPSLPVIKVKAVTMRNHAILQTLVGPGEEHTTLAGLPTEASIRNAVEEAIPGFLQNVYAHTAGGGKFLGILQVKKRQPSDEGRQGQAALIALATYSELKNIILVDEDVDIFDSDDILWAMTTRMQGDVSITTLPGIRGHQLDPSQSPDYSTSIRGNGISCKTIFDCTVPWALKARFERAPFMEVDPTPWAPELFSDKK*
ecdB		MRLIVGMTGATGAPLGVALLQALRDMPEVETHLVMSKWAKTTIELETPYTAQDVAALADVVHSPADQAATISSGSFRTDGMIVIPCSMKTLAGIRAGYAEGLVGRAADVVLKEGRKLVLVPRETPLSTIHLENMLALSRMGVAMVPPMPAYYNHPQTADDITQHIVTRVLDQFGLEHKKARRWNGLQAAKHFSQENNDGI*
ecdD		MICPRCADEQIEVMATSPVKGIWTVYQCQHCLYTWRDTEPLRRTSREHYPEAFRMTQKDIDEAPQVPTIPPLL*

Supplementary Table 2. Sequences of key mutations used to validate the tolerance mechanisms after ALE in this study.

Gene name	Sequence
*wzz**	ATGGAATTAAGGGAATACGCGACAATCCTGATGAAGAATTGGGTGTTGATCGTTATCGCATCCATTCTTGGAATTGCAGCGGGCGCTGGTTTTTCCCTTCTAGCTACACCGGAGTATC<br>AGTCACGTACTCAGTTGTATGTATCGGTGCGGTCGGGGGCTGGGACAACCTCTGACATGGTTCAGGGTGCTAACTTTTCGCGTCAGATTGTGAATAGTTATGTTGATGTCATTAAGAC<br>GGGTGTTGTTCTAGAGCCGGTTGTTGATGAGCTTGGTCTGGAGTTGACGGCTAACCAGTTGAGTTCTCATATCAGTGCGGCTTCTCCTGCTGATACTGCGTTGATCAACATCACTGCT<br>TCTAGTCCTTCTCCTCAGCAGGCGGCTGAGATCGCCAATGCAGTGGGGGAGAGTTTCAAGAATGTGATTCAGACTGAATTGGAACCAGACTCCGGCGATGGCATGAGCCCGATTAA<br>CCTGACTACTACTCAGGTGGCTCTGGAACCATCTTCTCCGGTCAGTCCTAATGTGATGATGAATATCCTCCTCGGCCTACTTGTAGGTCTTGCAATAGGTGTTGGTATTGCAGTGCTT<br>CGCGCGGCTTTGGATACTCGTATTCATTCCTTGCGCGATATTGAAGAGGTTACTGATAAGCCACTTTTGGGCGGAATCATTGCGGATTCTGAAGTTGAGAAGCACCCGTTGATCATTA<br>AGCACAAGCCGCATAGTCCTATTGCGGAGTCCTTTCGTGCGTTACGTACTAACCTGCAGTTCCTTAACGTCGGCGGCTCATCTTCAGTATTTGTTATCTCCTCTGCTAATCCTGGTGA<br>GGGTAAGTCAACCACTTCTGTAAACCTAGCTTTGGCGCTTGCAGAGGCCGGCTCCCGTGTAGCGCTGATCGAAGCTGATCTTCGCTTGCCACGAGTGAGCAAGTACCTCGGAGTTG<br>AGGGCAACGCAGGTCTGACTGACATTCTCATTGGCAAGGCCGAGGTTAATGATGTGTTGCAGCGTTGGGGTAGGACTCAGTTGTACTATCTCCCGGCAGGGCGCATTCCGCCGAAC<br>CCGAGTGAGTTGCTTGGTTCAGCTGAGATGGA**C**AAGGTCATTGCGGAGCTTGAGGAAAGCTTTGATTATGTGATCATCGATGCCCCTCCGGCGTTGGCGGTTACCGATGCTGCAGT<br>TATTGGTCATGGCAAGGCTGGCATCCTGATTGCGGTCTCCGCAGGTTCTACAAAGAAGCCTGAGTTGGAAGCTACGCTGTCCACGCTTGAGAATGCGGATGCCAATGTTGTTGGCG<br>TTGTCGCTACGATGCTCCCGCCTAAGTCTGTGGCTGGTTATGGCTACGGAAATTACGGCTACGGCGACACCTCCAAAATCAATGCCCCTAAGCCCGACAACACCGAACTAACCACCA<br>CCGATGCTTCCAAGGCCAACAATGAGCAATAG
*CGL_RS1820**	ATGTCAAATATTTTTTATGAAGGGATCCTGCAGAATCTAATTCAAGATGGGTGGGATGTGCACCTTGTTTCAAATCCAGGCCCAGTTGGGAAGAAATTACTGAATGGTAAAGCTCATAC<br>TATCGAGATGTCTAGAGAGATTTCAATAGGTACCGATGTTAAATCATTATTTAATTGGGTGCTATTGCTTAGGAAAATTCGTCCAAGAGTGCTGATAGTTGGAACTCCGAAAGCTTCAT<br>TGCTGGGAGTCGTGGCAGC~~**ACGAATTGCTCGAGTGCC**~~ACGAATTGTCTATGTGGCACATGGGCTGCGCTCTGAAACTGTTTTAGGCTTAAAAAAGAAGATTCTAGTGTTTTTGGAAT<br>ATTTGACCCAGTTGTTTGCACATCAAACTTTGGCTGTAAGTCATTCTCTGAAGAAAGCAATTGAAGATGCGCACCCTCGTTTTAAAGGAAGAGTGCAAGTCTTAGGTTATGGCAGTATG<br>AATGGAGTTGAGCTTGATCGCTTTAGAGTTCCATCCCTTGAAGAGAAATTATCTGCTCGTAATGCTTTAAACCTGCCTAGTAAATCTGTCATTGTTGGTTTTGTCGGCAGAATAAATAAA<br>GATAAGGGAGGAGATCTTCTCGCTGCTCTTACAAAACATGAGGCTTTTACCCGATTGCGACTGCATCTCTTAATTATTGGTGAATTGGAAGACGATGACTTGCGAGAAGCATTCATTA<br>AATTAGTTAATGAAGGGCAGGTTACGATTACAGGATGGATTGATTTCCCTGAAGAACCATTAGCTGCAGTTGATGTTTTGCTTCACCCAACTCAGCGAGAAGGTTTAGGTATGTCTTTG<br>CTGGAAGCTCAGGCTATGGGAGTGCCTGTATTGACGAATGCTGTGACTGGAACAGTTGATGCAGTAACAAGTGGAGAAGGTGGCTTTTTTGCCGATGACGATTCTGTTGAGTCCTGG<br>GTTTCTAAGATTGATTTATTAGTTTCCGATCCTAAGTTAAGAGACCGGATGGGACGTGCTGGTCGCCAGTTTGTGTCAGCTCGTTTCAATCGTGATGATGTCGCAGCTCGTTTCAGTC<br>ATTTCGTGGAACAATTCAAAAAATAG
*lexA** (upstream)	TTCAAAACTCCCGCCTCAATTGTTTGCCCACATCTTAACTTTTAGCCACGACACGTTCGAACACCAATACCGTTCACATGTTCGACTAAGTTTCTATGTTCGATTTATAGCACCCCGTTC<br>GAACGTTGTCTAGAGAAGATTTAAAAATCTCGAACACTCGTACCATTTCCGCAGGAAAACCTGTATGGTTGGAAACTAGAAATACTGACAGAGGTTCGAATAGCTCAGCATTCAGCTT<br>CAGAAGTTCACACTGGATGAACCCTCATCAAGAGTGAACATGGAAGCGAACAGAGTTTTTAGTGCTTTAACGGGCA**A**ACAATGAGAGGAAGGGAACGGAAG
atsR-1*<br>*(upstream 1)*	AGCATAGAACTAAAACAGTATGCTTGTCCTGCTAAATGTGAATTTTTAAGAGATTCATCCCACACAATTACGAAAAAACCAGATTGCCCACAAATTAGTCTTCGAACTTTAATCAACACC<br>ACACCCAAAGCTAAACTTGCGCTCGTTGTG**T**AAAATTGCATTCAGGCTGAAAAATTCCTAAAGGGACTCCGTCCGAATAATTGGAAAGCCCAGAAGAACAGTCAACTCCTAGAGTAAA<br>GGATAATC
atsR-2*<br>*(upstream 2)*	AGCATAGAACTAAAACAGTATGCTTGTCCTGCTAAATGTGAATTTTTAAGAGATTCATCCCACACAATTACGAAAAAACCAGATTGCCCACAAATTAGTCTTCGAACTTTAATCAACACC<br>ACACCCAAAGCTAAACTTGCGCTCGTTGTGTAAAATTGCATTCAGGCTGAAAAATTCCTAAAGGGACTCCGTCCGAATAATTGGAAAGCCCAGAAGAACAGTCAACTCCTAGA**G**TAAA<br>GGATAATC
atsR-3*	GTGTCGGGGCTTAGAGAAACAAAAAAGGCTGCTACGCGGACAGCATTGTCACGTGCTGCTGCGGAGATCGCCTTGATGGAAGGCCCTGAGGCGTTCACGGTTGCGGCCATTGCAG<br>CTGCAGCCGGGGTCTCCCCCAGGACTTTTCATAACTACTTCCCTTCTCGGGAAGACGCATTGGTGCAATTTGTGGTCATTCGCGTTCAGGAACTCACGGATCAGCTATACGAGTTTC<br>CTACAAGCGTG**CCCCC**ACGAGATGCCATTGAACAATTGGTGATAAACCAATTGCGGGATGGCGATGACGCTATGGATTCTTTCAGCGCAATGTTTCGAATCGGTGAGATCCTTGAAA<br>ATCTTGACCCCATCAAGTGTGTTATCGATAAGGAGCGACTCATTGCTCCGTTGCTTGAGTTCATGGTTGAACGTGACAAAGACCTTGACAAGTTCGATGCGGCAACTCTGATCCATTT<br>GCATGCAGCGGCAATTGCAACCTCGCTGCATACGTTTTACCAAGCTTCCGAGCCCCGGGACATAGAAGATGGAGTCGCATTGATCCGTCGGGCATGCGCCTGGATTAAGAAATAA

Supplementary Table 3. Operators of biosensors constructed and characterized in this study.

Allosteric transcription factor	Operator sequences	Reference
PhdR	ATAGTTTCGATCTTCAATCATTTGAACATGCATGCAT	This study
	GATGTAAGTTTTCAAAATTAATAGTTGACATTTTCAACGTTATGAG	
IhtR	AGCTTATCTTACGAACAAGATGTCCGGTAGATG	This study
PcaO	GTTCGCAAGGCGAAC	1
	AACCCCTGACCTTCGGGGTT	
BenR	GTCACACACCGCTT	2
	CCTAGGAATTCCCCTAAT	
	TTTAGGGCTATCCCTAGT	
GenR	ATTCCGCTATGTGGAATCTAAGTATTCCCCTCAGGAAACGGGGG	3
	ATAATCACTCTTTCCAGATAGCGGAATCCCTT	
	ATCACACGCACCAGAATCGGGAGG	
RolR	AAAATCTGAACCCTTGTTCATTTATGAAT	4
VanR	AACTAACTAAGAGTTTAGGTATTTGGTT	5

Supplementary references

1. Zhao, K.X., Huang, Y., Chen, X., Wang, N.X. & Liu, S.J. PcaO positively regulates pcaHG of the beta-ketoadipate pathway in *Corynebacterium glutamicum*. *J Bacteriol* **192**, 1565–1572 (2010).
2. Brinkrolf, K., Brune, I. & Tauch, A. Transcriptional regulation of catabolic pathways for aromatic compounds in *Corynebacterium glutamicum*. *Genetics and Molecular Research: GMR* **5**, 773–789 (2006).
3. Chao, H. & Zhou, N.Y. GenR, an IclR-type regulator, activates and represses the transcription of gen genes involved in 3-hydroxybenzoate and gentisate catabolism in *Corynebacterium glutamicum*. *J Bacteriol* **195**, 1598–1609 (2013).
4. Li, T. et al. The TetR-type transcriptional repressor RolR from *Corynebacterium glutamicum* regulates resorcinol catabolism by binding to a unique operator, rolO. *Appl Environ Microbiol* **78**, 6009–6016 (2012).
5. Morabbi Heravi, K., Lange, J., Watzlawick, H., Kalinowski, J. & Altenbuchner, J. Transcriptional regulation of the vanillate utilization genes (vanABK Operon) of *Corynebacterium glutamicum* by VanR, a PadR-like repressor. *J Bacteriol* **197**, 959–972 (2015).
