# Supplementary material for: Engineering a robust and autonomous biological funneling for efficient valorization of lignin-related aromatics
Source: Nat Commun. 2026 Jun 17;17:7659. doi: 10.1038/s41467-026-74424-y (PMC13434420; doi:10.1038/s41467-026-74424-y)
Supplement: Supplementary file 4 [file 41467_2026_74424_MOESM2_ESM.pdf]

Description of Additional Supplementary Files

File Name: Supplementary Data 1
Description: Summary of intersectional mutated genes in STALE1-STALE9.

File Name: Supplementary Data 2
Description: All bacterial strains used.

File Name: Supplementary Data 3
Description: All engineered plasmids and their information.

File Name: Supplementary Data 4
Description: Primers used to construct plasmids in this study.

File Name: Supplementary Data 5
Description: Raw data and fitting parameters for Isothermal titration calorimetry of *p*-CA with PhdR.

File Name: Supplementary Data 6
Description: Raw data and fitting parameters for Isothermal titration calorimetry of FA with PhdR.

File Name: Supplementary Data 7
Description: Raw data and fitting parameters for Isothermal titration calorimetry of CaA with PhdR.
